# Hydrosilylation of Esters via a Titanocene(III) Borohydride–PMHS System: Scope, Limitations, and Mechanistic Insights

**DOI:** 10.1021/acsomega.5c09007

**Published:** 2026-01-25

**Authors:** Godfred Fianu, Jenna Azar, Emmanuel Bulted, Elizabeth Jones, Robert A. Flowers

**Affiliations:** † Department of Chemistry, 1688 Moravian University, Bethlehem, Pennsylvania 18018, United States; ‡ Department of Chemistry, 1687 Lehigh University, Bethlehem, Pennsylvania 18015, United States

## Abstract

The hydrosilylation of aromatic and aliphatic esters using catalytic amounts of titanocene­(III) borohydride and poly­(methylhydrosiloxane) (PMHS) as the terminal reductant is described. Alcohol yields range from 43% to 99%, with select esters requiring the addition of isopropanol to promote reactivity. This modification is proposed to facilitate in situ formation of a more reactive titanocene­(III) hydride species, which appears necessary for efficient ester reduction. This ester reduction protocol is mild, cost-effective, operationally simple and has potential utility in broader reductive transformations.

## Graphical abstract

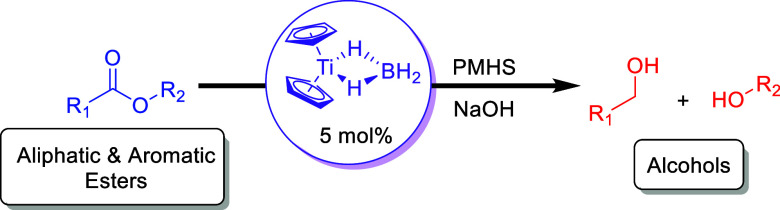

## Introduction

**T**itanocene­(III) complexes are highly versatile reagents widely employed in mediating various catalytic transformations. Their accessibility from readily available titanocene­(IV) precatalysts and ability to cycle between +3 and +4 oxidation states make them particularly effective catalysts for single-electron transfer (SET) processes. These complexes have been extensively utilized to open cyclic ethers such as epoxides and oxetanes, generating titanoxy radicals that undergo sequential single-electron transformations.^1–5^ These intermediates play a pivotal role in the total synthesis of biologically active compounds, including antitumoral alkaloids, lignans, terpenes, and antibiotics. Furthermore, titanocene­(III) complexes have demonstrated efficacy in mediating diverse coupling reactions, such as Wurtz-type homocoupling of terpenic allylic halides, Reformatsky additions, Pinacol couplings, McMurry-type couplings, Umpolung reactions, and Barbier-type reactions.^6–9^

The reduction of carbonyl compounds to alcohols has also been effectively facilitated by titanocene­(III) complexes. Oltra and Cuerva reported the reduction of aromatic ketones to alcohols using a titanocene­(III) aqua complex.^10–12^ Schwartz and co-workers also developed a system in which aromatic and aliphatic ketones were reduced to alcohols in aprotic media using stoichiometric amounts of titanocene­(III) borohydride (Scheme 1A).^13^ Buchwald and co-workers introduced an elegant carbonyl hydrosilylation method catalyzed by low-valent titanocene­(III) hydride catalysts with poly­(methylhydrosiloxane) (PMHS) as the stoichiometric reductant. This approach relied on generating the active titanocene hydride species either from a premade titanocene precatalyst or in situ under stringent low-temperature conditions from reagents requiring careful handling (Scheme 1B).^14–19^

**Scheme 1:**
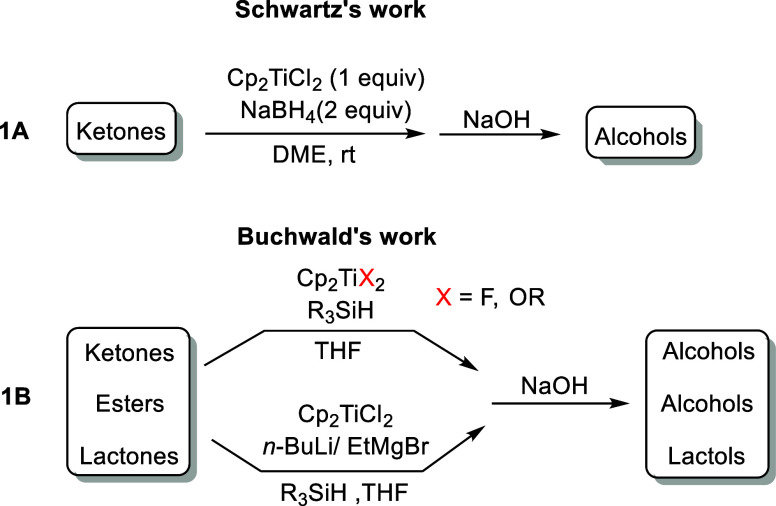
Previous Cp_2_Ti^(III)^-Catalyzed Carbonyl Reductions

Inspired by these foundational studies, Flowers and co-workers recently developed a more straightforward and efficient protocol for the reduction of ketones and aldehydes to alcohols.^20^ This method employs catalytic amounts of titanocene­(III) borohydride and PMHS, a cheap and readily available hydride source, as the stoichiometric reductant. Titanocene­(III) borohydride is easily generated under mild conditions at room temperature without the use of highly reactive organolithium or Grignard reagents. This offers a safer, more operationally convenient, and cost-effective alternative for catalytic hydrosilylation. Preliminary mechanistic studies indicate that a reactive titanocene­(III) hydride complex, which is formed in situ via σ-bond metathesis between a titanocene­(III) alkoxide and PMHS, mediates the reaction while maintaining its oxidation state throughout the process (Scheme 2A).^20^

**Scheme 2:**
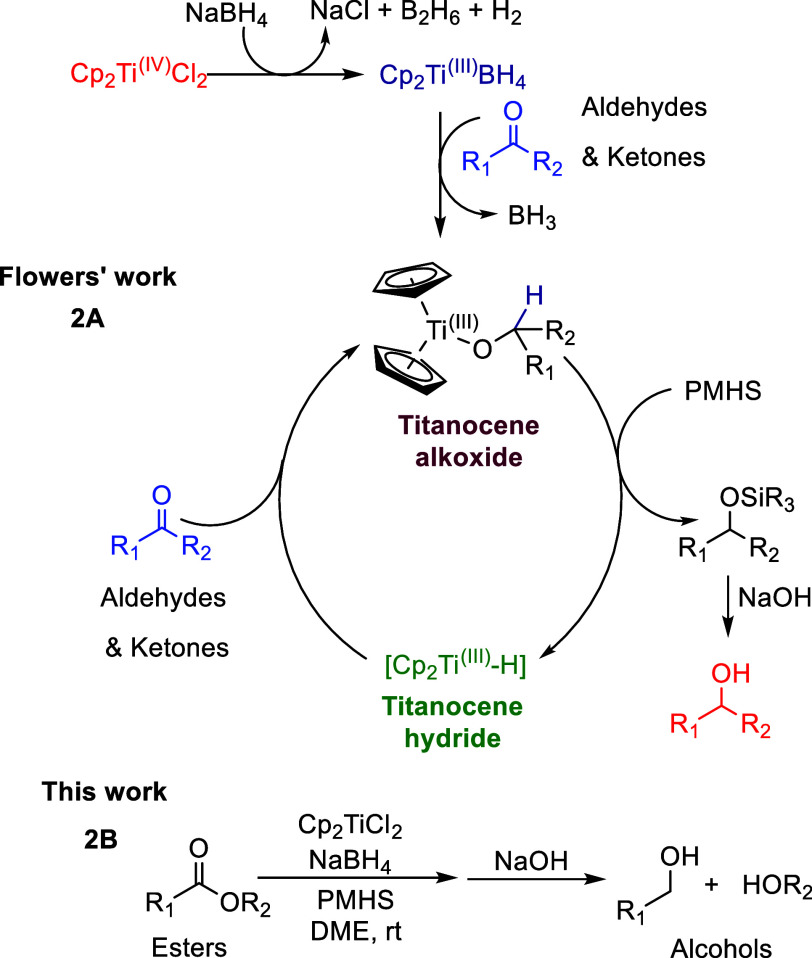
Cp_2_Ti^(III)^BH_4_-Catalyzed Hydrosilylations

The work presented herein investigates the applicability and efficiency of the titanocene­(III) borohydride-PMHS reduction protocol for the hydrosilylation of esters to produce alcohols (Scheme 2B). Ester reduction is a synthetically valuable yet more challenging transformation and is frequently used in multistep syntheses of complex molecules with biological and synthetic importance.^21–29^ This study also evaluates the limitations of the titanocene­(III) borohydride–PMHS system for ester reduction and examines structural factors influencing reactivity.

## Results and Discussion

A series of optimizing and control experiments were performed, and the results are summarized in Table 1. Methyl 3-phenylpropionante (**1a**) was cleanly reduced to 3-phenyl-1-propanol (**2a**) under the optimized reduction protocol outlined by Flowers (Table 1, entry 1).^20^ The hydrosilylation of **1a** to **2a** was also successful at 1 mol % catalyst loading (Table 1, entry 3) but consistent reduction across various esters necessitated the use of 5 mol % catalyst loading. The subsequent control experiment carried out showed that the titanocene catalyst is necessary to facilitate the hydrosilylation of **1a** to **2a** (Table 1, entry 5). Based on the mechanism proposed by Flowers, an active titanocene hydride is formed in situ after the initial carbonyl reduction by titanocene borohydride (Scheme 2A).^20^ We therefore hypothesized that the active titanocene­(III) hydride could be generated in situ via σ-bond metathesis between a titanocene­(III) alkoxide and PMHS. To form the titanocene hydride, titanocene borohydride was first treated with isopropanol (Table 1, entries 2 and 4) to form the titanocene alkoxide complex. PMHS was then added to generate the titanocene hydride complex. To this solution was added **1a**, which was successfully converted to **2a**.

**Table 1:**
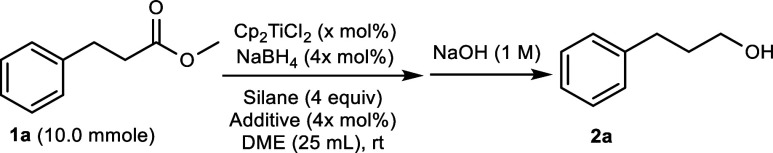
Optimizing Conditions and Control Experiments

entry	Cp_2_TiCl_2_ (mol %)	NaBH_4_ (mol %)	silane	additive	conv (%)^a^
1	5	20	PMHS	-	100
2	5	20	PMHS	*i*-PrOH	100
3	1	4	PMHS	-	100
4	1	4	PMHS	*i*-PrOH	100
5	-	20	PMHS	-	0
6	5	20	Et_2_SiH_2_	-	100^b^
7	5	20	PhSiH_3_	-	100^c^
8	5	20	Et_3_SiH	-	100
9	5	20	(EtO)_3_SiH	-	100
10	5	20	Me_2_PhSiH	-	100^b^

a Reactions were left to stir overnight and conversion of **1** **a** to **2a** was monitored by GCMS.

b Silane byproduct observed after workup.

c 1.5 equiv of PhSiH_3_ used.

Hydrosilylation of **1a** to ultimately form **2a** was also successful when PMHS was replaced with other silanes (Table 1, entries 6 to 10). However, silane polymer byproducts were present when either Et_2_SiH_2_ or Me_2_PhSiH was used as the hydride source (Table 1, entries 6 and 10). PMHS was used for the rest of the studies due to its low cost and effectiveness. Dimethoxyethane (DME) is a suitable polar aprotic solvent for making the active titanocene­(III) borohydride catalyst because NaBH_4_ is more soluble in DME than in THF. Attempts to run the reaction in THF did not lead to the formation of the active catalyst. No additional solvents were investigated, as DME uniquely enabled formation of the active titanocene­(III) borohydride catalyst under mild conditions and provided reproducible reactivity across the substrate scope.

To further investigate the breadth of this reduction protocol, a variety of esters with different steric and electronic properties were tested. The esters were further categorized as whether they possessed or lacked direct aromatic conjugation. The reactions carried out in this study were stirred overnight to ensure reaction completion. The results obtained from the hydrosilylation of the esters lacking direct aromatic conjugation and esters possessing direct aromatic conjugation are summarized in Tables 2 and 3 respectively.

**Table 2:**
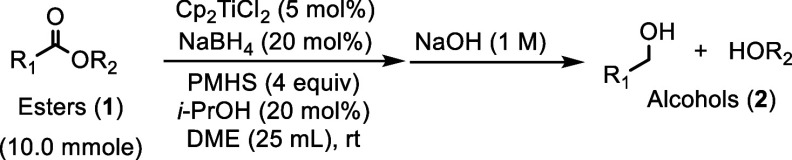

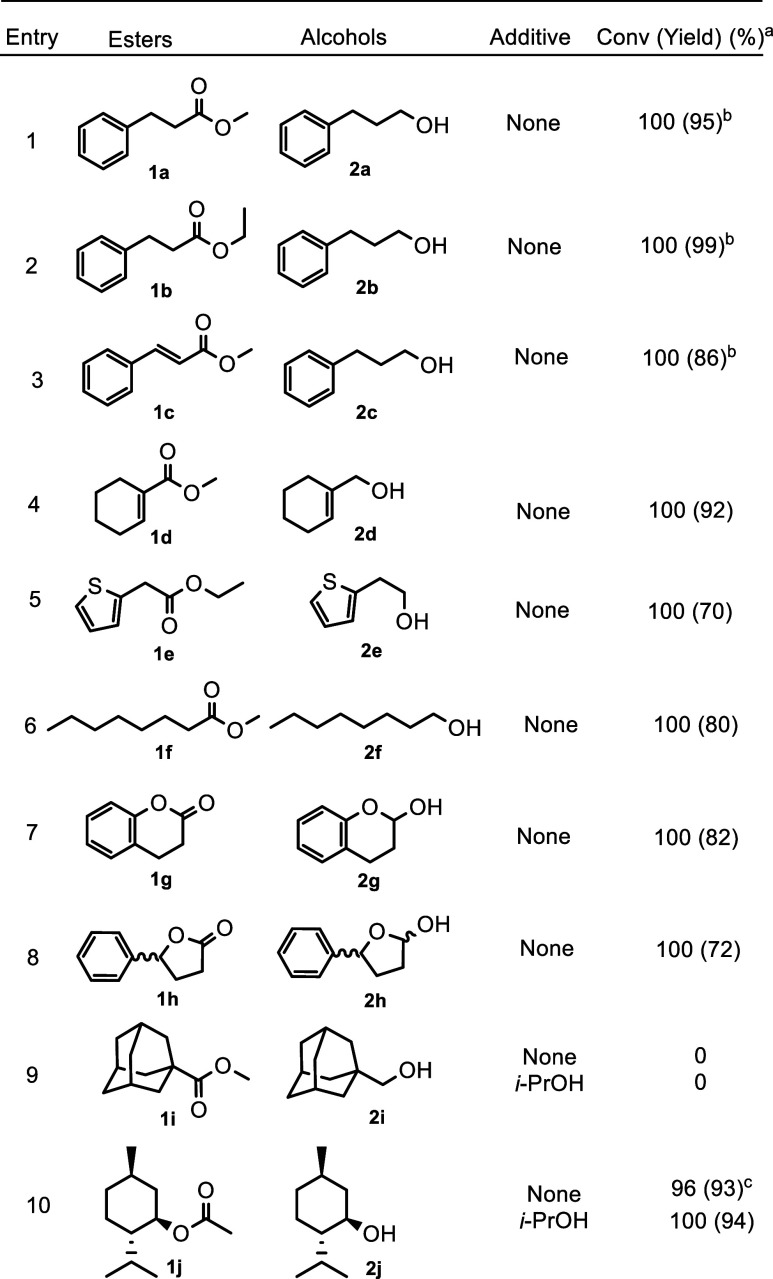
Esters Lacking Direct Aromatic Conjugation

a Reactions were left to stir overnight and were monitored by GCMS, with isolated yields in parentheses.

b 2.5 equiv of PMHS used.

c Conversion determined by NMR analysis.

**Table 3:**
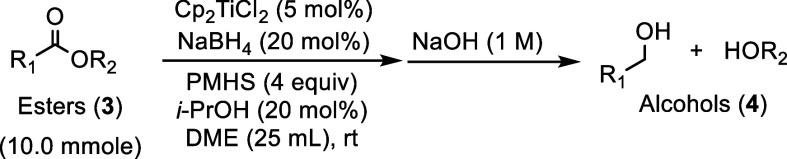

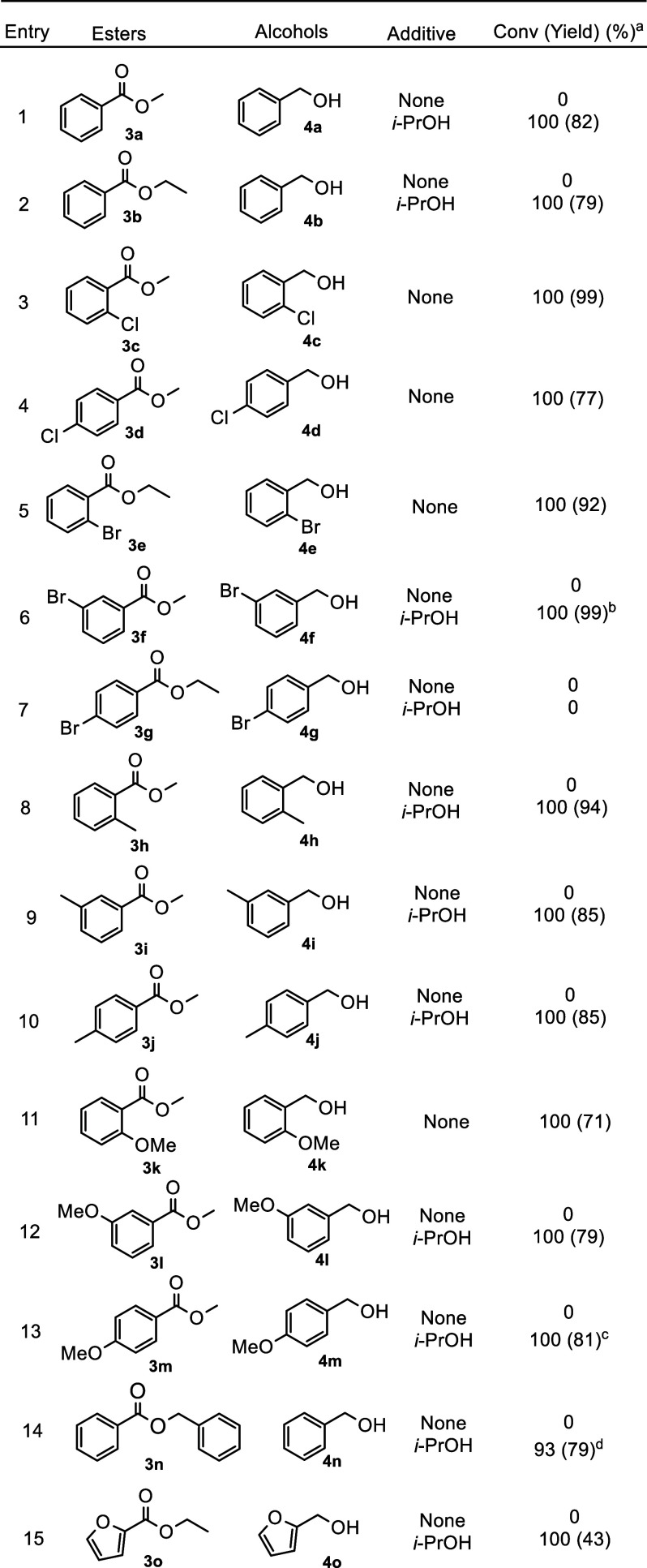
Esters Possessing Direct Aromatic Conjugation

a Reactions were left to stir overnight and were monitored by GCMS, with isolated yields in parentheses.

b 2.5 equiv of PMHS used.

c Reaction was done with 20 mol % of catalyst.

d Conversion determined by NMR analysis.

Esters lacking direct aromatic conjugation were generally reduced in moderate to high yields (Table 2). While the reduction protocol was effective in most cases, the cross-linking of the siloxane polymer with subsequent gel formation during workup lead to multiple filtrations in some cases, which impacted the isolated yields of the desired alcohol products **2a**, **2b** and **2c**.^30^ Based on our understanding of ester reductions by other hydride sources such as LiAlH_4_, we suspected that at least 2 equiv of PMHS is necessary to fully reduce the esters to the alcohol. As a result, selected reactions were repeated using reduced PMHS loadings, which led to improved isolated yields for certain substrates (Table 2, entries 1–3). However, lower PMHS equivalents were not general across the substrate scope. More challenging esters required higher PMHS loadings to achieve complete conversion. Consequently, 4 equiv of PMHS was selected as a standardized condition to ensure consistent and reproducible ester reduction across structurally diverse substrates. Further reduction of the PMHS loading below two equivalents resulted in incomplete conversion of **1a** and formation of multiple unidentified byproducts. Such conditions were therefore not pursued further.

The α,β-unsaturated ester, methyl cinnamate (**1c**), underwent full alkene reduction to afford saturated alcohol, 3-phenyl-1-propanol (**2c**) (Table 2, entry 3) whereas the internal alkene in methyl cyclohex-1-ene-1-carboxylate (**1d**) remained untouched (Table 2, entry 4). The complete hydrosilylation of the alkene in **1c** to form **2c** align with prior studies done by Han and co-workers on the hydrosilylations of olefins catalyzed by activated titanocene. It should be noted that the olefins tested in their studies were predominantly styrene derivatives and acyclic olefins.^31^ The chemoselective reduction of the ester and not the alkene observed in **1d** aligns with observations made by Buchwald and co-workers.^18^ The chemoselectivity observed with α,β-unsaturated esters under the reduction conditions could serve as a complement to the titanocene-catalyzed conjugate reduction of α,β-unsaturated carbonyls reported by Ashfeld.^32^

Lactones **1g** and **1h** were successfully transformed to their corresponding lactols **2g** and **2h** with **2h** isolated as a 1:1 mixture of cis/trans diastereomeric alcohols (Table 2, entries 7 and 8). These results corroborate with observations reported by Buchwald and co-workers.^16,17^ The sterically demanding substrate methyl adamantane-1-carboxylate (**1i**) was not reduced even at higher catalyst loadings and higher temperatures (Table 2, entry 9 and see Supporting Information). A plausible explanation for the lack of reactivity of methyl adamantane-1-carboxylate (**1i**) is the substantial steric congestion imposed by the rigid, cage-like adamantyl substituent directly adjacent to the ester carbonyl, which likely inhibits productive hydride transfer. While no systematic steric study was conducted, this interpretation is consistent with the unique structural features of this substrate relative to those successfully reduced.

Menthyl acetate (**1j**) was fully converted to product only when the isopropanol additive was added (Table 2, entry 10). Also, for many of the esters with direct aromatic conjugation, the addition of isopropanol was necessary to achieve conversion (Table 3). The isopropanol additive is proposed to promote the formation of the titanocene­(III) alkoxide intermediate that undergoes a σ-bond metathesis with PMHS to form a titanocene­(III) hydride complex.^20^ The titanocene­(III) hydride complex formed is responsible for mediating the ester hydrosilylation reaction. This modification to the reduction protocol outlined by Flowers and co-workers suggests that titanocene­(III) borohydride is not as reactive toward esters as it is toward aldehydes and ketones. The titanocene­(III) hydride that is formed in situ is however reactive toward most of the aromatic esters.

It was however noted that complete conversion was not achieved for benzyl benzoate (**3n**), extended reaction times did not result in further improvement. Conversions exceeding 90% were reproducible under these conditions and represent the practical limit of the current system for this substrate. Notably, **3n** was unreactive in the absence of isopropanol, highlighting the critical role of the additive in promoting ester reduction. It was also noted that the addition of isopropanol was consistently observed to reduce gel formation and simplify workup, although no quantitative study of this effect was performed.

Control experiments using a 1:1 mixture of ethyl 3-phenylpropionate (**1b**) and ethyl benzoate (**3b**) were conducted to evaluate the selective reduction of **1b** in the absence of isopropanol. Complete reduction of both esters to their corresponding alcohols was observed instead (Table 4). A plausible explanation is that titanocene­(III) borohydride initially catalyzes the reduction of **1b** to generate the proposed titanocene­(III) hydride intermediate in situ, which subsequently reacts with **3b**.

**Table 4:**
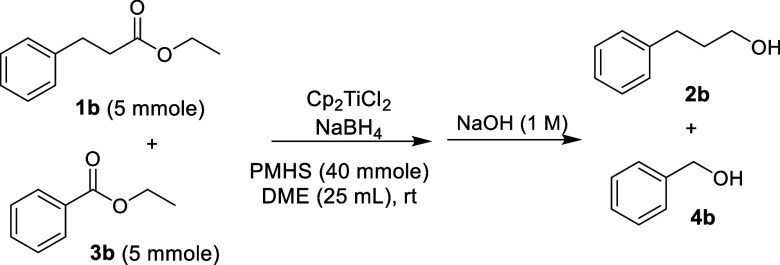
Results from Control Experiments

entry	Cp_2_TiCl_2_ (mmole)	NaBH_4_ (mmole)	conv. to 2b (yield) (%)^a^ ^,^ ^b^	conv. to 4b (yield) (%)^a^ ^,^ ^b^
1	0.25	1	100 (95)	100 (89)
2	0.125	0.5	100 (97)	100 (79)

a Conversion to product monitored by GCMS.

b Yield in parentheses calculated by NMR analysis.

It is possible that direct conjugation of the carbonyl group with the aromatic ring decreases the electrophilicity of the carbonyl carbon, thereby reducing the overall reactivity of the ester. Consequently, a more reactive titanocene­(III) hydride species is required to promote hydrosilylation in such cases. Although the precise influence of substitution pattern on reactivity is not fully understood at this stage, the observed trends likely result from the interplay of two competing factors: (I) activation of the carbonyl carbon through inductive withdrawal by electronegative substituents, and (II) resonance stabilization of the carbonyl group by substituents capable of π-donation.

For esters **3c–3e**, the substituents appear to activate the carbonyl group inductively, enhancing reactivity relative to the unsubstituted esters **3a** and **3b**. In contrast, **3f** (methyl 3-bromobenzoate) displays diminished activation due to the lower electronegativity of bromine compared to chlorine and its meta orientation, which limits inductive effects. The para-substituted ester **3g** (ethyl 4-bromobenzoate) was unreactive toward hydrosilylation, even under elevated catalyst loadings and temperatures (Table 3, entry 7). This lack of reactivity may arise from the reduced inductive contribution of bromine combined with resonance stabilization of the carbonyl group through conjugation.

Similarly, for the methoxy-substituted esters, **3k** (*ortho*-methoxy) exhibited increased reactivity, likely due to the proximity of the electronegative oxygen atom, which enhances carbonyl activation via induction (Table 3, entry 11). In contrast, **3l** and **3m** (meta- and *para*-methoxy derivatives) displayed lower reactivity (Table 3, entries 12 and 13). Notably, **3m** was less reactive than the unsubstituted esters (**3a** & **3b**). This reduced reactivity can be rationalized by the diminished inductive effect of the para substituent and the resonance stabilization imparted by the electron-donating methoxy group. Repeating the reaction under reflux led to a lower conversion of **3m** to **4m** (See Supporting Information). Complete reduction of **3m** to the corresponding alcohol **4m** was only realized when the reaction was run at higher catalyst loadings and at room temperature (Table 3, entry 13). These results indicate that increased thermal energy does not compensate for insufficient catalyst concentration and that catalyst loading, rather than temperature, governs conversion efficiency for this substrate.

Overall, these observations underscore the delicate balance between inductive activation and resonance stabilization in determining the reactivity of substituted aromatic esters toward titanocene­(III)-mediated hydrosilylation.

## Conclusions

In summary, we report a mild, operationally simple, and cost-effective protocol for the hydrosilylation of esters to alcohols using a titanocene borohydride–PMHS system. The method exhibits broad reactivity with a wide range of aliphatic and aromatic esters in good to excellent yields. In certain cases, however, isopropanol was required, likely facilitating in situ formation of the reactive titanocene­(III) hydride species. Future efforts will focus on further optimization of hydride efficiency and expansion of substrate scope, as well as mechanistic studies to better define the factors governing ester reactivity. The method shows potential for application in complex molecule synthesis, especially where selective reduction of unactivated esters is necessary. Ongoing studies to refine and extend this approach to the hydrosilylation of amides and nitriles are underway and will be reported in due course.

## Experimental Section

### General Information

Unless otherwise stated, all reactions were carried out in the glovebox under argon atmosphere. A MBraun solvent purification system was used to purify all the solvents used for experiments. All reagents and chemicals, mostly argon or nitrogen flushed, were purchased from reputable chemical vendors (Alfa Aesar, Acros, Sigma-Aldrich, Thermo Scientific, and TCI) and used without further purification. Chemicals not flushed with an inert gas were degassed with argon and used without any additional purification protocols. 1H NMR spectra were measured on a Bruker 400 MHz spectrometer in deuterated chloroform (CDCl3).13C NMR spectra were measured at 101 MHz in CDCl3. GC–MS analyses were done with a Shimadzu GCMS-2010 series with a SH-Rxi-5Sil MS (30m) column.

### General Procedure for Ester Reduction (GP1)

A 50-mL round-bottomed flask (RBF) was charged with titanocene dichloride (Cp_2_TiCl_2_) (0.5 mmol,125 mg) and sodium borohydride (NaBH4) (2 mmol, 76 mg). To this was added 25 mL dimethoxyethane (DME) and left to stir until a violet-colored solution was formed, indicative of the formation of titanocene borohydride (Cp_2_TiBH_4_). The ester (10 mmol) was then added followed by the addition of Polymethylhydrosiloxane (PMHS) (40 mmol, 2.40 mL). The solution was stirred overnight.

The solution was taken out of the glovebox and exposed to air to quench the catalyst followed by dropwise addition of 1 M NaOH solution to quench the excess PMHS (Note: vigorous bubbling observed with NaOH addition). The mixture was stirred until bubbling stopped and clear layers were observed (Note: for good yields of alcohol products, the mixture was stirred overnight). The alcohol product was extracted two times with 50 mL of diethyl ether. Organic layers were combined, filtered and washed with about 20 mL 1 M NaOH, followed by 10 mL brine solution then dried with Na_2_SO_4_. The solution was evaporated to dryness to obtain the isolated yield. Product identity and purity were determined with GC–MS and NMR.

NMR and GC–MS analyses were performed after aqueous workup; analysis of unworked reaction mixtures was avoided due to interference from solvent and silane byproducts and the risk of GC column fouling.

### General Procedure for Ester Reduction (GP2)

A 50-mL round-bottomed flask (RBF) was charged with titanocene dichloride (Cp_2_TiCl_2_) (0.5 mmol,125 mg) and sodium borohydride (NaBH_4_) (2 mmol, 76 mg). To this was added 25 mL dimethoxyethane (DME) and left to stir until a violet-colored solution was formed, indicative of the formation of titanocene borohydride (Cp_2_TiBH_4_). The ester (10 mmol) was then added followed by the addition of Polymethylhydrosiloxane (PMHS) (40 mmol, 2.40 mL). Isopropanol (2 mmol, 0.15 mL) was added dropwise to the reaction mixture (Note: some bubbling observed) and left to stir overnight.

Note: there was no difference in results when isopropanol was added before the addition of the ester and PMHS.

The solution was taken out of the glovebox and exposed to air to quench the catalyst followed by dropwise addition of 1 M NaOH solution to quench the excess PMHS (Note: vigorous bubbling observed with NaOH addition). The mixture was stirred until bubbling stopped and clear layers were observed (Note: for good yields of alcohol products, the mixture was stirred overnight). The alcohol product was extracted two times with 50 mL of diethyl ether. Organic layers were combined, filtered and washed with about 20 mL 1 M NaOH, followed by 10 mL brine solution then dried with Na_2_SO_4_. The solution was evaporated to dryness to obtain the isolated yield. Product identity and purity were determined with GC–MS and NMR.

#### 3-Phenyl-1-propanol (**2a**)

3-phenyl-1-propanol (**2a**) was prepared from methyl 3-phenylpropionate (**1a**) by the procedure outlined in **GP1** (Note: 2.5 equiv of PMHS used). GCMS analysis showed 100% conversion to product and 95% isolated yield upon complete workup. 1H NMR (400 MHz, CDCl_3_): δ 7.17–7.09 (m, 2H), 7.04 (d, *J* = 7.0 Hz, 3H), 3.48 (t, *J* = 6.5 Hz, 2H), 2.55 (dd, *J* = 17.9, 10.4 Hz, 3H), 1.72 (dq, *J* = 7.4, 6.5 Hz, 2H). 13C NMR (101 MHz, CDCl_3_): δ 141.95, 128.50, 128.46, 125.92, 62.11, 34.23, 32.14.

#### 3-Phenyl-1-propanol (**2b**)

3-phenyl-1-propanol (**2b**) was prepared from ethyl 3-phenylpropionate (**1b**) by the procedure outlined in **GP1** (Note: 2.5 equiv of PMHS used). GCMS analysis showed 100% conversion to product and 99% isolated yield upon complete workup. 1H NMR (400 MHz, CDCl_3_): δ 7.17–7.09 (m, 2H), 7.04 (d, *J* = 6.8 Hz, 3H), 3.48 (t, *J* = 6.4 Hz, 2H), 2.53 (t, *J* = 7.6 Hz, 2H), 2.45 (d, *J* = 6.5 Hz, 1H), 1.72 (dd, *J* = 8.1, 7.1 Hz, 2H). 13C NMR (101 MHz, CDCl_3_): δ 142.12, 128.62, 128.57, 126.01, 61.95, 34.33, 32.26.

#### 3-Phenyl-1-propanol (**2c**)

3-phenyl-1-propanol (**2c**) was prepared from ethyl 3-phenylpropionate (**1c**) by the procedure outlined in **GP1** (Note: 2.5 equiv of PMHS used). GCMS analysis showed 100% conversion to product and 86% isolated yield upon complete workup. 1H NMR (400 MHz, CDCl_3_): δ 7.13 (t, *J* = 6.6 Hz, 2H), 7.04 (d, *J* = 7.3 Hz, 3H), 3.48 (t, *J* = 6.5 Hz, 2H), 2.54 (t, *J* = 7.9 Hz, 2H), 2.01 (s, 1H), 1.72 (p, *J* = 7.1 Hz, 2H). 13C NMR (101 MHz, CDCl_3_): δ 141.93, 128.68, 128.51, 128.47, 125.93, 62.18, 34.25, 32.14.

#### Cyclohex-1-en-1-ylmethanol (**2d**)

Cyclohex-1-en-1-ylmethanol (**2d**) was prepared from methyl cyclohex-1-ene-1-carboxylate (**1d**) by the procedure outlined in **GP1**. GCMS analysis showed 100% conversion to product and 92% isolated yield upon complete workup. 1H NMR (400 MHz, CDCl_3_): δ 5.68 (s, 1H), 3.97 (s, 2H), 2.07–1.97 (m, 4H), 1.87 (s, 1H), 1.69–1.56 (m, 4H). 13C NMR (101 MHz, CDCl_3_): δ 137.54, 122.95, 77.40, 77.08, 76.76, 67.58, 25.60, 24.92, 22.54, 22.44.

#### 2-thiophenylethanol (**2e**)

2-thiophenylethanol (**2e**) was prepared from ethyl 2-thiopheneacetate (**1e**) by the procedure outlined in **GP1**. GCMS analysis showed 100% conversion to product and 70% isolated yield upon complete workup. 1H NMR (400 MHz, CDCl_3_): δ 6.99 (d, *J* = 4.3 Hz, 1H), 6.82–6.73 (m, 1H), 6.70 (s, 1H), 3.65 (t, *J* = 6.4 Hz, 2H), 2.89 (t, *J* = 6.4 Hz, 2H), 2.28 (s, 1H). 13C NMR (101 MHz, CDCl_3_): δ 140.90, 127.03, 125.57, 123.98, 63.45, 33.27.

#### **1-octanol** (**2f**)

1-octanol (**2f**) was prepared from methyl octanoate (**1f**) by the procedure outlined in **GP1**. GCMS analysis showed 100% conversion to product and 80% isolated yield upon complete workup. 1H NMR (400 MHz, CDCl_3_): δ 3.49 (q, *J* = 6.2 Hz, 2H), 2.49 (d, *J* = 5.1 Hz, 1H), 1.43 (q, *J* = 7.0 Hz, 2H), 1.18 (d, *J* = 10.8 Hz, 10H), 0.76 (t, *J* = 6.5 Hz, 3H). 13C NMR (101 MHz, CDCl_3_): δ 62.77, 32.71, 31.82, 29.42, 29.29, 25.77, 22.64, 14.05.

#### Chroman-2-ol (**2g**)

Chroman-2-ol (**2g**) was prepared from chroman-2-one (**1g**) by the procedure outlined in **GP1**. GCMS analysis showed 100% conversion to product and 82% isolated yield upon complete workup. 1H NMR (400 MHz, CDCl_3_): δ 7.00–6.88 (m, 2H), 6.77–6.63 (m, 2H), 5.43 (t, *J* = 3.3 Hz, 1H), 3.44 (s, 1H), 2.82 (ddd, *J* = 16.6, 10.2, 6.5 Hz, 1H), 2.55 (dq, *J* = 16.6, 6.0 Hz, 1H), 1.98–1.74 (m, 2H). 13C NMR (101 MHz, CDCl_3_): δ 152.02, 129.35, 127.48, 122.10, 120.92, 116.90, 92.21, 27.09, 20.38.

#### 5-phenyltetrahydrofuran-2-ol (**2h**)

5-phenyltetrahydrofuran-2-ol (**2h**) was prepared from (±)-γ-phenyl-γ-butyrolactone (**1h**) by the procedure outlined in **GP1**. GCMS analysis showed 100% conversion to product and 72% isolated yield upon complete workup. NMR analysis shows a 1:1 mixture of cis/trans stereoisomers of product present that could not be isolated. 1H NMR (400 MHz, CDCl_3_) cis diastereomer: δ 7.33–7.11 (m, 5H), 5.60–4.10 (m, 1H), 3.64 (s, 1H), 3.50–3.41 (m, 1H), 2.41–1.39 (m, 4H). Trans diastereomer : δ 7.33–7.11 (m, 5H), 5.60–4.10 (m, 1H), 3.50–3.41 (m, 1H), 3.17 (s, 1H), 2.41–1.39 (m, 4H). 13C NMR (101 MHz, CDCl_3_) mixture of diastereomers: δ 144.77, 142.82, 142.41, 128.41, 127.53, 127.44, 127.39, 126.44, 125.86, 125.74, 98.56, 82.90, 76.82, 74.19, 62.61, 36.29, 34.51, 33.14, 32.86, 32.80, 29.10.

#### (−)-Menthol (**2j**)

(−)-Menthol (**2j**) was prepared from (−)-menthyl acetate (**1j**) by the procedure outlined in **GP1**. GCMS and NMR analysis showed 96% conversion to product and 93% isolated yield upon complete workup. The reaction was repeated using the procedure outlined in **GP2**. GCMS analysis showed 100% conversion to product and 94% isolated yield upon complete workup. 1H NMR (400 MHz, CDCl_3_): δ 3.29 (td, *J* = 10.5, 4.9 Hz, 1H), 2.05 (pd, *J* = 7.0, 2.8 Hz, 1H), 1.88–1.80 (m, 1H), 1.51 (ddq, *J* = 18.9, 12.6, 3.0 Hz, 3H), 1.32–1.24 (m, 2H), 1.12–1.03 (m, 1H), 0.99 (ddt, *J* = 12.9, 10.0, 3.0 Hz, 2H), 0.80 (t, *J* = 6.4 Hz, 6H), 0.68 (d, *J* = 6.9 Hz, 3H). 13C NMR (101 MHz, CDCl_3_): δ 71.55, 50.15, 45.07, 34.56, 31.66, 25.84, 23.14, 22.23, 21.03, 16.10.

#### Benzyl alcohol (**4a**)

Benzyl alcohol (**4a**) was prepared from methyl benzoate (**3a**) by the procedure outlined in **GP2**. GCMS analysis showed 100% conversion and 82% isolated yield upon complete workup. The reaction did not work with the procedure outlined in **GP1**.

1H NMR (400 MHz, CDCl3): δ 7.52–6.93 (m, 5H), 4.46 (s, 2H), 2.57 (s, 1H). 13C NMR (101 MHz, CDCl3): δ 140.89, 128.57, 127.63, 127.06, 65.15.

#### Benzyl alcohol (**4b**)

Benzyl alcohol (**4b**) was prepared from ethyl benzoate (**3b**) by the procedure outlined in **GP2**. GCMS analysis showed 100% conversion to product and 79% isolated yield upon complete workup. The reaction did not work with the procedure outlined in **GP1**. 1H NMR (400 MHz, CDCl_3_): δ 7.40–6.96 (m, 5H), 4.48 (s, 2H), 2.72 (s, 1H). 13C NMR (101 MHz, CDCl_3_): δ 140.92, 128.56, 127.62, 127.06, 65.11.

#### 2-Chlorobenzyl alcohol (**4c**)

2-Chlorobenzyl alcohol (**4c**) was prepared from methyl 2-chlorobenzoate (**3c**) by the procedure outlined in **GP1**. GCMS analysis showed 100% conversion to product and 99% isolated yield upon complete workup. 1H NMR (400 MHz, CDCl_3_): δ 7.30 (d, *J* = 5.3 Hz, 1H), 7.19 (d, *J* = 7.6 Hz, 1H), 7.14–7.03 (m, 2H), 4.59 (s, 2H), 2.18 (s, 1H). 13C NMR (101 MHz, CDCl_3_): δ 138.18, 132.70, 129.35, 128.84, 128.72, 127.04, 77.41, 77.09, 76.77, 62.79.

#### 4-Chlorobenzyl alcohol (**4d**)

4-Chlorobenzyl alcohol (**4d**) was prepared from methyl 4-chlorobenzoate (**3d**) by the procedure outlined in **GP1**. GCMS analysis showed 100% conversion to product and 77% isolated yield upon complete workup. 1H NMR (400 MHz, CDCl_3_): δ 7.36 (d, *J* = 8.6 Hz, 2H), 7.31 (d, *J* = 8.5 Hz, 3H), 4.68 (s, 2H), 1.91 (s, 1H). 13C NMR (101 MHz, CDCl_3_): δ 139.26, 133.37, 128.70, 128.30, 77.38, 77.06, 76.74, 64.55.

#### 2-Bromobenzyl alcohol (**4e**)

2-Bromobenzyl alcohol (**4e**) was prepared from ethyl 2-bromobenzoate (**3e**) by the procedure outlined in **GP1**. GCMS analysis showed 100% conversion to product and 92% isolated yield upon complete workup. 1H NMR (400 MHz, CDCl_3_): δ 7.28 (dd, *J* = 33.1, 7.7 Hz, 2H), 7.10 (t, *J* = 7.5 Hz, 1H), 6.94 (t, *J* = 7.6 Hz, 1H), 4.48 (s, 2H), 3.00 (s, 1H). 13C NMR (101 MHz, CDCl_3_): δ 139.76, 132.54, 129.02, 128.72, 127.64, 122.45, 64.75.

#### 3-Bromobenzyl alcohol (**4f**)

3-Bromobenzyl alcohol (**4f**) was prepared from methyl 3-bromobenzoate (**3f**) by the procedure outlined in **GP2** (NOTE: 2.5 equiv of PMHS used). GCMS analysis showed 100% conversion to product and 99% isolated yield upon complete workup. The reaction did not work with the procedure outlined in **GP1**. 1H NMR (400 MHz, CDCl_3_): δ 7.31 (s, 1H), 7.23 (d, *J* = 7.3 Hz, 1H), 7.09–7.00 (m, 2H), 4.42 (s, 2H), 2.67 (s, 1H). 13C NMR (101 MHz, CDCl_3_): δ 143.12, 130.60, 130.13, 129.88, 125.37, 122.62, 64.26.

#### 2-methylbenzyl alcohol (**4h**)

2-methylbenzyl alcohol (**4h**) was prepared from methyl 2-methylbenzoate (**3h**) by the procedure outlined in **GP2**. GCMS analysis showed 100% conversion to product and 94% isolated yield upon complete workup. The reaction did not work with the procedure outlined in **GP1**. 1H NMR (400 MHz, CDCl_3_): δ 7.46–7.40 (m, 1H), 7.37–7.24 (m, 3H), 4.63 (s, 2H), 3.81 (s, 1H), 2.39 (s, 3H). 13C NMR (101 MHz, CDCl_3_): δ 138.89, 136.04, 130.28, 127.65, 127.54, 126.09, 62.87, 18.70.

#### 3-methylbenzyl alcohol (**4i**)

3-methylbenzyl alcohol (**4i**) was prepared from methyl 3-methylbenzoate (**3i**) by the procedure outlined in **GP2**. GCMS analysis showed 100% conversion to product and 85% isolated yield upon complete workup. The reaction did not work with the procedure outlined in **GP1**. 1H NMR (400 MHz, CDCl_3_): δ 7.35 (t, *J* = 7.4 Hz, 1H), 7.29–7.08 (m, 3H), 4.63 (s, 2H), 4.03 (s, 1H), 2.47 (s, 3H). 13C NMR (101 MHz, CDCl_3_): δ 141.07, 138.11, 128.49, 128.28, 127.90, 124.22, 64.77, 21.50.

#### 4-methylbenzyl alcohol (**4j**)

4-methylbenzyl alcohol (**4j**) was prepared from methyl 4-methylbenzoate (**3j**) by the procedure outlined in **GP2**. GCMS analysis showed 100% conversion to product and 85% isolated yield upon complete workup. The reaction did not work with the procedure outlined in **GP1**. 1H NMR (400 MHz, CDCl_3_): δ 7.10 (d, *J* = 8.0 Hz, 2H), 7.03 (d, *J* = 8.0 Hz, 2H), 4.47 (s, 2H), 2.21 (s, 3H), 1.95 (s, 1H). 13C NMR (101 MHz, CDCl_3_): δ 137.94, 137.39, 129.26, 127.16, 65.20, 21.19.

#### 2-methoxybenzyl alcohol (**4k**)

2-methoxybenzyl alcohol (**4k**) was prepared from methyl 2-methoxybenzoate (**3k**) by the procedure outlined in **GP1**. GCMS analysis showed 100% conversion to product and 71% isolated yield upon complete workup. 1H NMR (400 MHz, CDCl_3_): δ 7.16–7.07 (m, 2H), 6.77 (t, *J* = 7.5 Hz, 1H), 6.69 (d, *J* = 8.1 Hz, 1H), 4.50 (d, *J* = 5.7 Hz, 2H), 3.65 (s, 3H), 2.75 (s, 1H). 13C NMR (101 MHz, CDCl_3_): δ 157.33, 129.23, 128.83, 128.60, 120.65, 110.20, 61.61, 55.26.

#### 3-methoxybenzyl alcohol (**4**l)

3-methoxybenzyl alcohol (**4l**) was prepared from methyl 3-methoxybenzoate (**3l**) by the procedure outlined in **GP2**. GCMS analysis showed 100% conversion to product and 79% isolated yield upon complete workup. The reaction did not work with the procedure outlined in **GP1**. 1H NMR (400 MHz, CDCl_3_): δ 7.26 (t, *J* = 8.1 Hz, 1H), 6.91 (s, 2H), 6.83 (d, *J* = 7.7 Hz, 1H), 4.56 (s, 2H), 4.15 (s, 1H), 3.75 (s, 3H). 13C NMR (101 MHz, CDCl_3_): δ 159.71, 142.82, 129.51, 119.22, 113.04, 112.28, 64.56, 55.12, 55.08.

#### 4-methoxybenzyl alcohol (**4m**)

4-methoxybenzyl alcohol (**4m**) was prepared from methyl 4-methoxybenzoate (**3 m**) by the procedure outlined in **GP2** but with 20 mol % of the catalyst. GCMS analysis showed 100% conversion to product and 81% isolated yield upon complete workup. The reaction did not work with the procedure outlined in **GP1**. 1H NMR (400 MHz, CDCl_3_): δ 7.06 (d, *J* = 8.6 Hz, 2H), 6.69 (d, *J* = 8.2 Hz, 2H), 4.33 (s, 2H), 3.60 (s, 3H), 3.31 (s, 1H). 13C NMR (101 MHz, CDCl_3_): δ 159.00, 133.31, 128.64, 113.86, 64.48, 55.26.

#### Benzyl alcohol (**4n**)

Benzyl alcohol (**4n**) was prepared from benzyl benzoate (**4n**) by the procedure outlined in **GP2**. GCMS analysis showed 93% conversion to product and 79% isolated yield upon complete workup. The reaction did not work with the procedure outlined in **GP1**. 1H NMR (400 MHz, CDCl_3_): δ 7.52–6.93 (m, 5H), 4.46 (s, 2H), 2.57 (s, 1H). 13C NMR (101 MHz, CDCl_3_): δ 140.89, 128.57, 127.63, 127.06, 65.15.

#### 2-Furfuryl alcohol (**4o**)

2-Furfuryl alcohol (**4o**) was prepared from ethyl 2-furancarboxylate (**3o**) by the procedure outlined in **GP2**. GCMS analysis showed 100% conversion to product and 43% isolated yield upon complete workup. The reaction did not work with the procedure outlined in **GP1**. Attempts to improve isolated yields by reducing the concentration of PMHS to 2.5 equiv did not lead to full conversion of **3o** to product (**4o**). 1H NMR (400 MHz, CDCl_3_): δ 7.23 (d, *J* = 0.5 Hz, 1H), 6.18 (s, 1H), 6.12 (s, 1H), 4.38 (s, 2H), 3.11 (s, 1H). 13C NMR (101 MHz, CDCl_3_): δ 154.15, 142.45, 110.35, 107.68, 57.04.

## Supplementary Material



## Data Availability

The data underlying this study are available in the article and its Supporting Information.

## Notes

Experimental procedures, spectral characterization data–NMR spectra, and GC–MS of all synthesized compounds (PDF) (PDF)
