# Supplementary material for: Hydrosilylation of Esters via a Titanocene(III) Borohydride–PMHS System: Scope, Limitations, and Mechanistic Insights
Source: ACS Omega. 2026 Jan 25;11(5):7672–9. doi: 10.1021/acsomega.5c09007 (PMC12903028; doi:10.1021/acsomega.5c09007)
Supplement: Supplementary file 1 [file ao5c09007_si_001.pdf]

Supporting Information

Hydrosilylation of Esters via a Titanocene(III) Borohydride–PMHS System: Scope, Limitations, and Mechanistic Insights

Godfred Fianu*, [a], Jenna Azar[a], Emmanuel Bulted[a], Elizabeth Jones[a] and Robert A. Flowers II[b]

[a]Department of Chemistry, Moravian University, Bethlehem, PA 18018, USA

[b]Department of Chemistry, Lehigh University, Bethlehem, PA 18015, USA

Table of Contents

General Information....S2

(GP 1) General procedure for ester reduction....S2

(GP 2) General procedure for ester reduction with isopropanol additive....S2

Reduction of esters....S3

Control experiments....S11

Effects of catalyst loading and heat on the reduction of **1i** and **3m**....S11

References....S12

NMR spectra for compounds....S13

NMR for control experiments....S37

GC-MS data for compounds....S38

GC-MS for control experiments....S63

GC-MS data on effects of catalyst loading and heat on the reduction of **1i** and **3m**....S65

General Information

Unless otherwise stated, all reactions were carried out in the glove box under argon atmosphere. A MBraun solvent purification system was used to purify all the solvents used for experiments. All reagents and chemicals, mostly argon or nitrogen flushed, were purchased from reputable chemical vendors (Alfa Aesar, Acros, Sigma Aldrich, Thermo Scientific, and TCI) and used without further purification. Chemicals not flushed with an inert gas were degassed with argon and used without any additional purification protocols. 1H-NMR spectra were measured on a Bruker 400MHz spectrometer in deuterated chloroform ($CDCl_3$).13C-NMR spectra were measured at 101 MHz in $CDCl_3$. GC-MS analyses were done with a Shimadzu GCMS-2010 series with a SH-Rxi-5Sil MS (30m) column.

1. **(GP 1) General procedure for ester reduction**

   All the reactions were carried out in the glove box under argon atmosphere. A 50-mL round-bottomed flask (RBF) was charged with titanocene dichloride (Cp_2TiCl_2) (0.5 mmole,125 mg) and sodium borohydride ($NaBH_4$) (2 mmole, 76 mg). To this was added 25 mL dimethoxyethane (DME) and left to stir till a violet-colored solution was formed, indicative of the formation of titanocene borohydride (Cp_2TiBH_4). The ester (10 mmole) was then added followed by the addition of Polymethylhydrosiloxane (PMHS) (40 mmole, 2.40 mL). The solution was stirred overnight.

   The solution was taken out of the glovebox and exposed to air to quench the catalyst followed by dropwise addition of 1 M NaOH solution to quench the excess PMHS (Note: vigorous bubbling observed with NaOH addition). The mixture was stirred until bubbling stopped and clear layers were observed (Note: For good yields of alcohol products, the mixture was stirred overnight). The alcohol product was extracted two times with 50 mL of diethyl ether. Organic layers were combined, filtered and washed with about 20 mL 1 M NaOH, followed by 10 mL brine solution then dried with Na_2SO_4. The solution was evaporated to dryness to obtain the isolated yield. Product identity and purity were determined with GC-MS and NMR.

   Note: NMR and GC-MS analyses were performed after aqueous work-up; analysis of unworked reaction mixtures was avoided due to interference from solvent and silane byproducts and the risk of GC column fouling.

2. **(GP 2) General procedure for ester reduction with isopropanol additive.**

   All the reactions were carried out in the glove box under argon atmosphere. A 50-mL round-bottomed flask (RBF) was charged with titanocene dichloride (Cp_2TiCl_2) (0.5 mmole,125 mg) and sodium borohydride ($NaBH_4$) (2 mmole, 76 mg). To this was added 25 mL dimethoxyethane (DME) and left to stir till a violet-colored solution was formed, indicative of the formation of titanocene borohydride (Cp_2TiBH_4). The ester (10 mmole) was then added followed by the addition of (Polymethylhydrosiloxane (PMHS) (40 mmole, 2.40 mL). Isopropanol (2 mmole, 0.15 mL) was added dropwise to the reaction mixture (Note: Some bubbling observed) and left to stir overnight.

   NOTE: There was no difference in results when isopropanol was added before the addition of the ester and PMHS.

The solution was taken out of the glovebox and exposed to air to quench the catalyst followed by dropwise addition of 1 M NaOH solution to quench the excess PMHS (Note: vigorous bubbling observed with NaOH addition). The mixture was stirred until bubbling stopped and clear layers were observed (Note: For good yields of alcohol products, the mixture was stirred overnight). The alcohol product was extracted two times with 50 mL of diethyl ether. Organic layers were combined, filtered and washed with about 20 mL 1 M NaOH, followed by 10 mL brine solution then dried with Na_2SO_4. The solution was evaporated to dryness to obtain the isolated yield. Product identity and purity were determined with GC-MS and NMR.

NOTE: NMR and GC-MS analyses were performed after aqueous work-up; analysis of unworked reaction mixtures was avoided due to interference from solvent and silane byproducts and the risk of GC column fouling.

3. Reduction of esters

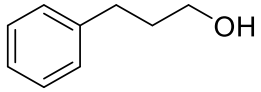

Figure S1. 3-phenyl-1-propanol (**2a**)

3-phenyl-1-propanol (**2a**) was prepared from methyl 3-phenylpropionate (**1a**) by the procedure outlined in **GP1** (Note: 2.5 equivalents of PMHS used). GCMS analysis showed 100 % conversion to product and 95 % isolated yield upon complete workup. The NMR spectra of the alcohol product are consistent with published spectra.[1]

^{1}H NMR (400 MHz, $CDCl_3$) δ 7.17 – 7.09 (m, 2H), 7.04 (d, *J* = 7.0 Hz, 3H), 3.48 (t, *J* = 6.5 Hz, 2H), 2.55 (dd, *J* = 17.9, 10.4 Hz, 3H), 1.72 (dq, *J* = 7.4, 6.5 Hz, 2H). ^{13}C NMR (101 MHz, $CDCl_3$) δ 141.95, 128.50, 128.46, 125.92, 62.11, 34.23, 32.14.

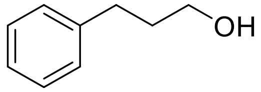

Figure S2. 3-phenyl-1-propanol (**2b**)

3-phenyl-1-propanol (**2b**) was prepared from ethyl 3-phenylpropionate (**1b**) by the procedure outlined in **GP1** (Note: 2.5 equivalents of PMHS used). GCMS analysis showed 100 % conversion to product and 99 % isolated yield upon complete workup. The NMR spectra of the alcohol product are consistent with published spectra.[1]

^{1}H NMR (400 MHz, $CDCl_3$) δ 7.17 – 7.09 (m, 2H), 7.04 (d, *J* = 6.8 Hz, 3H), 3.48 (t, *J* = 6.4 Hz, 2H), 2.53 (t, *J* = 7.6 Hz, 2H), 2.45 (d, *J* = 6.5 Hz, 1H), 1.72 (dd, *J* = 8.1, 7.1 Hz, 2H). ^{13}C NMR (101 MHz, $CDCl_3$) δ 142.12, 128.62, 128.57, 126.01, 61.95, 34.33, 32.26.

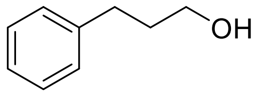

Figure S3. 3-phenyl-1-propanol (**2c**)

3-phenyl-1-propanol (**2c**) was prepared from methyl cinnamate (**1c**) by the procedure outlined in **GP1**. GCMS analysis showed 100 % conversion to product and 86 % isolated yield upon complete workup (NOTE: 2.5 equivalents of PMHS used). The NMR spectra of the alcohol product are consistent with published spectra.[1]

^{1}H NMR (400 MHz, $CDCl_3$) δ 7.13 (t, *J* = 6.6 Hz, 2H), 7.04 (d, *J* = 7.3 Hz, 3H), 3.48 (t, *J* = 6.5 Hz, 2H), 2.54 (t, *J* = 7.9 Hz, 2H), 2.01 (s, 1H), 1.72 (p, *J* = 7.1 Hz, 2H). ^{13}C NMR (101 MHz, $CDCl_3$) δ 141.93, 128.68, 128.51, 128.47, 125.93, 62.18, 34.25, 32.14.

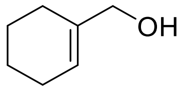

Figure S4. Cyclohex-1-en-1-ylmethanol (**2d**)

Cyclohex-1-en-1-ylmethanol (**2d**) was prepared from methyl cyclohex-1-ene-1-carboxylate (**1d**) by the procedure outlined in **GP1**. GCMS analysis showed 100 % conversion to product and 92 % isolated yield upon complete workup. The NMR spectra of the alcohol product are consistent with published spectra.[2]

^{1}H NMR (400 MHz, $CDCl_3$) δ 5.68 (s, 1H), 3.97 (s, 2H), 2.07 – 1.97 (m, 4H), 1.87 (s, 1H), 1.69 – 1.56 (m, 4H). ^{13}C NMR (101 MHz, $CDCl_3$) δ 137.54, 122.95, 77.40, 77.08, 76.76, 67.58, 25.60, 24.92, 22.54, 22.44.

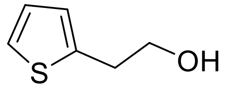

Figure S5. 2-thiophenylethanol (**2e**)

2-thiophenylethanol (**2e**) was prepared from ethyl 2-thiopheneacetate (**1e**) by the procedure outlined in **GP1**. GCMS analysis showed 100 % conversion to product and 70 % isolated yield upon complete workup. The NMR spectra of the alcohol product are consistent with published spectra.[3]

^{1}H NMR (400 MHz, $CDCl_3$) δ 6.99 (d, *J* = 4.3 Hz, 1H), 6.82 – 6.73 (m, 1H), 6.70 (s, 1H), 3.65 (t, *J* = 6.4 Hz, 2H), 2.89 (t, *J* = 6.4 Hz, 2H), 2.28 (s, 1H). ^{13}C NMR (101 MHz, $CDCl_3$) δ 140.90, 127.03, 125.57, 123.98, 63.45, 33.27.

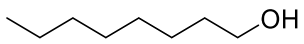

Figure S6. 1-Octanol (**2f**)

1-octanol (**2f**) was prepared from methyl octanoate (**1f**) by the procedure outlined in **GP1**. GCMS analysis showed 100 % conversion to product and 80 % isolated yield upon complete workup. The NMR spectra of the alcohol product are consistent with published spectra.[2]

^{1}H NMR (400 MHz, $CDCl_3$) δ 3.49 (q, *J* = 6.2 Hz, 2H), 2.49 (d, *J* = 5.1 Hz, 1H), 1.43 (q, *J* = 7.0 Hz, 2H), 1.18 (d, *J* = 10.8 Hz, 10H), 0.76 (t, *J* = 6.5 Hz, 3H). ^{13}C NMR (101 MHz, $CDCl_3$) δ 62.77, 32.71, 31.82, 29.42, 29.29, 25.77, 22.64, 14.05.

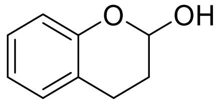

Figure S7. Chroman-2-ol (**2g**)

Chroman-2-ol (**2g**) was prepared from chroman-2-one (**1g**) by the procedure outlined in **GP1**. GCMS analysis showed 100 % conversion to product and 82 % isolated yield upon complete workup. The NMR spectra of the alcohol product are consistent with published spectra.[4]

^{1}H NMR (400 MHz, $CDCl_3$) δ 7.00 – 6.88 (m, 2H), 6.77 – 6.63 (m, 2H), 5.43 (t, *J* = 3.3 Hz, 1H), 3.44 (s, 1H), 2.82 (ddd, *J* = 16.6, 10.2, 6.5 Hz, 1H), 2.55 (dq, *J* = 16.6, 6.0 Hz, 1H), 1.98 – 1.74 (m, 2H). ^{13}C NMR (101 MHz, $CDCl_3$) δ 152.02, 129.35, 127.48, 122.10, 120.92, 116.90, 92.21, 27.09, 20.38.

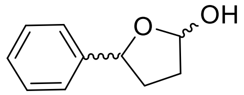

Figure S8. 5-phenyltetrahydrofuran-2-ol (**2h**)

5-phenyltetrahydrofuran-2-ol (**2h**) was prepared from (±)-γ-phenyl-γ-butyrolactone (**1h**) by the procedure outlined in **GP1**. GCMS analysis showed 100 % conversion to product and 72 % isolated yield upon complete workup. NMR analysis shows a 1:1 mixture of cis/trans stereoisomers of product present that could not be isolated. The NMR spectra of the alcohol product are consistent with published spectra.[4]

^{1}H NMR (400 MHz, $CDCl_3$) cis diastereomer δ 7.33 – 7.11 (m, 5H), 5.60 – 4.10 (m, 1H), 3.64 (s, 1H), 3.50 – 3.41 (m, 1H), 2.41 – 1.39 (m, 4H). Trans diastereomer δ 7.33 – 7.11 (m, 5H), 5.60 – 4.10 (m, 1H), 3.50 – 3.41 (m, 1H), 3.17 (s, 1H), 2.41 – 1.39 (m, 4H). ^{13}C NMR (101 MHz, $CDCl_3$) mixture of diastereomers δ 144.77, 142.82, 142.41, 128.41, 127.53, 127.44, 127.39, 126.44, 125.86, 125.74, 98.56, 82.90, 76.82, 74.19, 62.61, 36.29, 34.51, 33.14, 32.86, 32.80, 29.10.

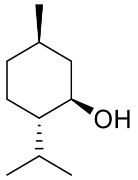

Figure S9. (-)-Menthol (**2j**)

(-)-Menthol (**2j**) was prepared from (-)-menthyl acetate (**1j**) by the procedure outlined in **GP1**. GCMS and NMR analysis showed 96 % conversion to product and 93 % isolated yield upon complete workup. The reaction was repeated using the procedure outlined in **GP2**. GCMS analysis showed 100 % conversion to product and 94 % isolated yield upon complete workup. The NMR spectra of the alcohol product are consistent with published spectra.[2]

^{1}H NMR (400 MHz, $CDCl_3$) δ 3.29 (td, *J* = 10.5, 4.9 Hz, 1H), 2.05 (pd, *J* = 7.0, 2.8 Hz, 1H), 1.88 – 1.80 (m, 1H), 1.51 (ddq, *J* = 18.9, 12.6, 3.0 Hz, 3H), 1.32 – 1.24 (m, 2H), 1.12 – 1.03 (m, 1H), 0.99 (ddt, *J* = 12.9, 10.0, 3.0 Hz, 2H), 0.80 (t, *J* = 6.4 Hz, 6H), 0.68 (d, *J* = 6.9 Hz, 3H). ^{13}C NMR (101 MHz, $CDCl_3$) δ 71.55, 50.15, 45.07, 34.56, 31.66, 25.84, 23.14, 22.23, 21.03, 16.10.

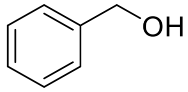

Figure S10. Benzyl alcohol (**4a**)

Benzyl alcohol (**4a**) was prepared from methyl benzoate (**3a**) by the procedure outlined in **GP2**. GCMS analysis showed 100 % conversion and 82 % isolated yield upon complete workup. The reaction did not work with the procedure outlined in **GP1**. The NMR spectra of the alcohol product are consistent with published spectra.[2]

^{1}H NMR (400 MHz, $CDCl_3$) δ 7.52 – 6.93 (m, 5H), 4.46 (s, 2H), 2.57 (s, 1H). ^{13}C NMR (101 MHz, $CDCl_3$) δ 140.89, 128.57, 127.63, 127.06, 65.15.

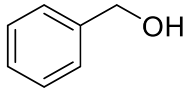

Figure S11. Benzyl alcohol (**4b**)

Benzyl alcohol (**4b**) was prepared from ethyl benzoate (**3b**) by the procedure outlined in **GP2**. GCMS analysis showed 100 % conversion to product and 79 % isolated yield upon complete workup. The reaction did not work with the procedure outlined in **GP1**. The NMR spectra of the alcohol product are consistent with published spectra.[2]

^{1}H NMR (400 MHz, $CDCl_3$) δ 7.40 – 6.96 (m, 5H), 4.48 (s, 2H), 2.72 (s, 1H). ^{13}C NMR (101 MHz, $CDCl_3$) δ 140.92, 128.56, 127.62, 127.06, 65.11.

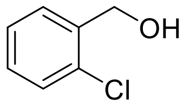

Figure S12. 2-Chlorobenzyl alcohol (**4c**)

2-Chlorobenzyl alcohol (**4c**) was prepared from methyl 2-chlorobenzoate (**3c**) by the procedure outlined in **GP1**. GCMS analysis showed 100 % conversion to product and 99 % isolated yield upon complete workup. The NMR spectra of the alcohol product are consistent with published spectra.[5]

^{1}H NMR (400 MHz, $CDCl_3$) δ 7.30 (d, *J* = 5.3 Hz, 1H), 7.19 (d, *J* = 7.6 Hz, 1H), 7.14 – 7.03 (m, 2H), 4.59 (s, 2H), 2.18 (s, 1H). ^{13}C NMR (101 MHz, $CDCl_3$) δ 138.18, 132.70, 129.35, 128.84, 128.72, 127.04, 77.41, 77.09, 76.77, 62.79.

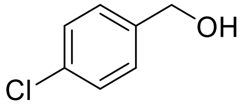

Figure S13. 4-Chlorobenzyl alcohol (**4d**)

4-Chlorobenzyl alcohol (**4d**) was prepared from methyl 4-chlorobenzoate (**3d**) by the procedure outlined in **GP1**. GCMS analysis showed 100 % conversion to product and 77 % isolated yield upon complete workup. The NMR spectra of the alcohol product are consistent with published spectra.[2]

^{1}H NMR (400 MHz, $CDCl_3$) δ 7.36 (d, *J* = 8.6 Hz, 2H), 7.31 (d, *J* = 8.5 Hz, 3H), 4.68 (s, 2H), 1.91 (s, 1H). ^{13}C NMR (101 MHz, $CDCl_3$) δ 139.26, 133.37, 128.70, 128.30, 77.38, 77.06, 76.74, 64.55.

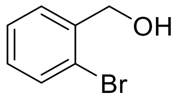

Figure S14. 2-Bromobenzyl alcohol (**4e**)

2-Bromobenzyl alcohol (**4e)** was prepared from ethyl 2-bromobenzoate (**3e**) by the procedure outlined in **GP1**. GCMS analysis showed 100 % conversion to product and 92 % isolated yield upon complete workup. The NMR spectra of the alcohol product are consistent with published spectra.[3]

^{1}H NMR (400 MHz, $CDCl_3$) δ 7.28 (dd, *J* = 33.1, 7.7 Hz, 2H), 7.10 (t, *J* = 7.5 Hz, 1H), 6.94 (t, *J* = 7.6 Hz, 1H), 4.48 (s, 2H), 3.00 (s, 1H). ^{13}C NMR (101 MHz, $CDCl_3$) δ 139.76, 132.54, 129.02, 128.72, 127.64, 122.45, 64.75.

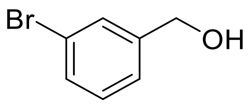

Figure S15. 3-Bromobenzyl alcohol (**4f**)

3-Bromobenzyl alcohol (**4f**) was prepared from methyl 3-bromobenzoate (**3f**) by the procedure outlined in **GP2** (NOTE: 2.5 equivalents of PMHS used). GCMS analysis showed 100 % conversion to product and 99 % isolated yield upon complete workup. The reaction did not work with the procedure outlined in **GP1**. The NMR spectra of the alcohol product are consistent with published spectra.[5]

^{1}H NMR (400 MHz, $CDCl_3$) δ 7.31 (s, 1H), 7.23 (d, *J* = 7.3 Hz, 1H), 7.09 – 7.00 (m, 2H), 4.42 (s, 2H), 2.67 (s, 1H). ^{13}C NMR (101 MHz, $CDCl_3$) δ 143.12, 130.60, 130.13, 129.88, 125.37, 122.62, 64.26.

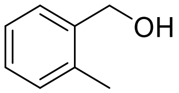

Figure S16. 2-methylbenzyl alcohol (**4h**)

2-methylbenzyl alcohol (**4h**) was prepared from methyl 2-methylbenzoate (**3h**) by the procedure outlined in **GP2**. GCMS analysis showed 100 % conversion to product and 94 % isolated yield upon complete workup. The reaction did not work with the procedure outlined in **GP1**. The NMR spectra of the alcohol product are consistent with published spectra.[5]

^{1}H NMR (400 MHz, $CDCl_3$) δ 7.46 – 7.40 (m, 1H), 7.37 – 7.24 (m, 3H), 4.63 (s, 2H), 3.81 (s, 1H), 2.39 (s, 3H). ^{13}C NMR (101 MHz, $CDCl_3$) δ 138.89, 136.04, 130.28, 127.65, 127.54, 126.09, 62.87, 18.70.

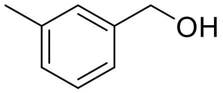

Figure S17. 3-methylbenzyl alcohol (**4i**)

3-methylbenzyl alcohol (**4i**) was prepared from methyl 3-methylbenzoate (**3i**) by the procedure outlined in **GP2**. GCMS analysis showed 100 % conversion to product and 85 % isolated yield upon complete workup. The reaction did not work with the procedure outlined in **GP1**. The NMR spectra of the alcohol product are consistent with published spectra.[5]

^{1}H NMR (400 MHz, $CDCl_3$) δ 7.35 (t, *J* = 7.4 Hz, 1H), 7.29 – 7.08 (m, 3H), 4.63 (s, 2H), 4.03 (s, 1H), 2.47 (s, 3H). ^{13}C NMR (101 MHz, $CDCl_3$) δ 141.07, 138.11, 128.49, 128.28, 127.90, 124.22, 64.77, 21.50.

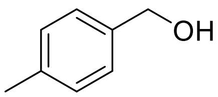

Figure S18. 4-methylbenzyl alcohol (**4j**)

4-methylbenzyl alcohol (**4j**) was prepared from methyl 4-methylbenzoate (**3j**) by the procedure outlined in **GP2**. GCMS analysis showed 100 % conversion to product and 85 % isolated yield upon complete workup. The reaction did not work with the procedure outlined in **GP1**. The NMR spectra of the alcohol product are consistent with published spectra.[6]

^{1}H NMR (400 MHz, $CDCl_3$) δ 7.10 (d, *J* = 8.0 Hz, 2H), 7.03 (d, *J* = 8.0 Hz, 2H), 4.47 (s, 2H), 2.21 (s, 3H), 1.95 (s, 1H). ^{13}C NMR (101 MHz, $CDCl_3$) δ 137.94, 137.39, 129.26, 127.16, 65.20, 21.19.

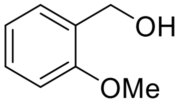

Figure S19. 2-methoxybenzyl alcohol (**4k**)

2-methoxybenzyl alcohol (**4k**) was prepared from methyl 2-methoxybenzoate (**3k**) by the procedure outlined in **GP1**. GCMS analysis showed 100 % conversion to product and 71 % isolated yield upon complete workup. The NMR spectra of the alcohol product are consistent with published spectra.[7]

^{1}H NMR (400 MHz, $CDCl_3$) δ 7.16 – 7.07 (m, 2H), 6.77 (t, *J* = 7.5 Hz, 1H), 6.69 (d, *J* = 8.1 Hz, 1H), 4.50 (d, *J* = 5.7 Hz, 2H), 3.65 (s, 3H), 2.75 (s, 1H). ^{13}C NMR (101 MHz, $CDCl_3$) δ 157.33, 129.23, 128.83, 128.60, 120.65, 110.20, 61.61, 55.26.

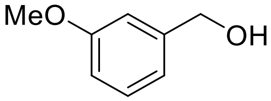

Figure S20. 3-methoxybenzyl alcohol (**4l**)

3-methoxybenzyl alcohol (**4l**) was prepared from methyl 3-methoxybenzoate (**3l)** by the procedure outlined in **GP2.** GCMS analysis showed 100 % conversion to product and 79 % isolated yield upon complete workup. The reaction did not work with the procedure outlined in **GP1**. The NMR spectra of the alcohol product are consistent with published spectra.[8]

^{1}H NMR (400 MHz, $CDCl_3$) δ 7.26 (t, *J* = 8.1 Hz, 1H), 6.91 (s, 2H), 6.83 (d, *J* = 7.7 Hz, 1H), 4.56 (s, 2H), 4.15 (s, 1H), 3.75 (s, 3H). ^{13}C NMR (101 MHz, $CDCl_3$) δ 159.71, 142.82, 129.51, 119.22, 113.04, 112.28, 64.56, 55.12, 55.08.

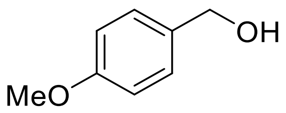

Figure S21. 4-methoxybenzyl alcohol (**4m**)

4-methoxybenzyl alcohol (**4m**) was prepared from methyl 4-methoxybenzoate (**3-m**) by the procedure outlined in **GP2** but with 20 mol% of the catalyst. GCMS analysis showed 100 % conversion to product and 81 % isolated yield upon complete workup. The reaction did not work with the procedure outlined in **GP1**. The NMR spectra of the alcohol product are consistent with published spectra.[2]

^{1}H NMR (400 MHz, $CDCl_3$) δ 7.06 (d, *J* = 8.6 Hz, 2H), 6.69 (d, *J* = 8.2 Hz, 2H), 4.33 (s, 2H), 3.60 (s, 3H), 3.31 (s, 1H). ^{13}C NMR (101 MHz, $CDCl_3$) δ 159.00, 133.31, 128.64, 113.86, 64.48, 55.26.

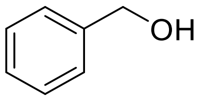

Figure S22. Benzyl alcohol (**4n**)

Benzyl alcohol (**4n**) was prepared from benzyl benzoate (**4n**) by the procedure outlined in **GP2**. GCMS analysis showed 93 % conversion to product and 79 % isolated yield upon complete workup. The reaction did not work with the procedure outlined in **GP1**. The NMR spectra of the alcohol product are consistent with published spectra.[2]

^{1}H NMR (400 MHz, $CDCl_3$) δ 7.52 – 6.93 (m, 5H), 4.46 (s, 2H), 2.57 (s, 1H). ^{13}C NMR (101 MHz, $CDCl_3$) δ 140.89, 128.57, 127.63, 127.06, 65.15.

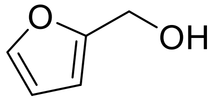

Figure S23. 2-Furfuryl alcohol (**4o**)

2-Furfuryl alcohol (**4o**) was prepared from ethyl 2-furancarboxylate (**3o**) by the procedure outlined in **GP2**. GCMS analysis showed 100 % conversion to product and 43 % isolated yield upon complete workup. The reaction did not work with the procedure outlined in **GP1**. Attempts to improve isolated yields by reducing the concentration of PMHS to 2.5 equivalents did not lead to full conversion of **3o** to product (**4o**). The NMR spectra of the alcohol product are consistent with published spectra.[2]

^{1}H NMR (400 MHz, $CDCl_3$) δ 7.23 (d, *J* = 0.5 Hz, 1H), 6.18 (s, 1H), 6.12 (s, 1H), 4.38 (s, 2H), 3.11 (s, 1H). ^{13}C NMR (101 MHz, $CDCl_3$) δ 154.15, 142.45, 110.35, 107.68, 57.04.

4. **Control experiments**

Two control experiments using a 1:1 mixture of ethyl 3-phenylpropionate (**1b**) and ethyl benzoate (**3b**) were conducted to evaluate the selective reduction of **1b** in the absence of isopropanol by following the procedure outlined in **GP1**.

Table S1. Results from control experiments

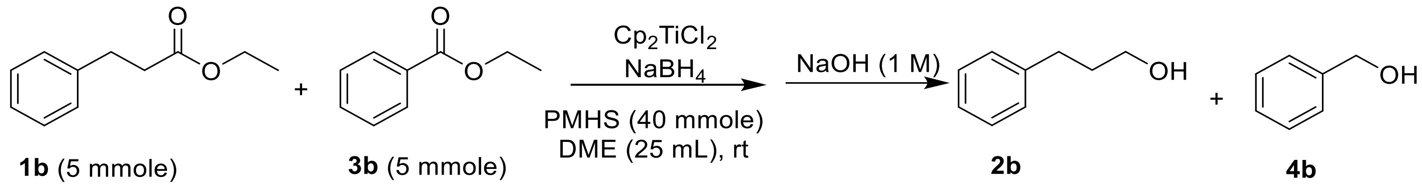

Experiment	Cp_2TiCl_2 (mmole)	$NaBH_4$ (mmole)	Conv. to **2b** (%)[a,b]	Conv. to **4b** (%)[a,b]
1	0.25	1	100 (95)	100 (89)
2	0.125	0.5	100 (97)	100 (79)

[a] Conversion to product monitored by GCMS. [b] Yield in parenthesis calculated by NMR analysis

5. **Effects of catalyst loading and heat on the reduction of 1i and 3m**

The study on the effects of catalyst loading and heat on the reduction of 1i and 3m were done by following the procedure outlined in **GP2**.

Table S2. Results from the reduction of **1i** with different catalyst loadings and at different temperatures

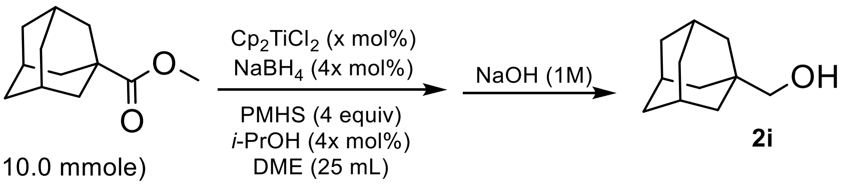

Experiment	Cp_2TiCl_2 (mol%)	Temperature (°C)	Conv (%)[a]
1	5	r.t.	0
2	40	r.t.	0
3	40	85	0

[a] Conversion to product monitored by GCMS.

Table S3. Results from the reduction of **3m** with different catalyst loadings and at different temperatures

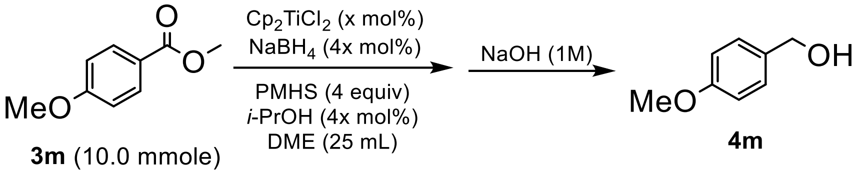

3m (10.0 mmole)

4m

Experiment	Cp_2TiCl_2 (mol%)	Temperature (°C)	Conv (%)[a]
1	5	r.t.	96
2	5	85	82
3	20	r.t.	100

[a] Conversion determined from the area% of **4m** on GCMS.

6. References

(1) Buchwald, S. L.; Berk, S. C.; Kreutzer, K. A. A Catalytic Method for the Reduction of Esters to Alcohols. *J. Am. Chem. Soc.* **1991**, *113*, 5095–5097.

(2) Werkmeister, S.; Junge, K.; Wendt, B.; Alberico, E.; Jiao, H.; Baumann, W.; Junge, H.; Gallou, F.; Beller, M. Hydrogenation of Esters to Alcohols with a Well-Defined Iron Complex. *Angew. Chem. Int. Ed.* **2014**, *53* (33), 8722–8726.

(3) Reding, M. T.; Buchwald, S. L. Inexpensive Air-Stable Titanium-Based System for the Conversion of Esters to Primary Alcohols. *J. Org. Chem.* **1995**, *60* (24), 7884–7890.

(4) Verdaguer, X.; Hansen, M. C.; Berk, S. C.; Buchwald, S. L. Titanocene-Catalyzed Reduction of Lactones to Lactols. *J. Org. Chem.* **1997**, *62* (24), 8522–8528.

(5) Wang, R.; Tang, Y.; Xu, M.; Meng, C.; Li, F. Transfer Hydrogenation of Aldehydes and Ketones with Isopropanol under Neutral Conditions Catalyzed by a Metal-Ligand Bifunctional Catalyst [Cp*Ir(2,2'-BpyO)(H_2O)]. *J. Org. Chem.* **2018**, *83* (4), 2274–2281.

(6) Mukhopadhyay, T. K.; Rock, C. L.; Hong, M.; Ashley, D. C.; Groy, T. L.; Baik, M. H.; Trovitch, R. J. Mechanistic Investigation of Bis(Imino)Pyridine Manganese Catalyzed Carbonyl and Carboxylate Hydrosilylation. *J. Am. Chem. Soc.* **2017**, *139* (13), 4901–4915.

(7) Tamang, S. R.; Bedi, D.; Shafiei-Haghighi, S.; Smith, C. R.; Crawford, C.; Findlater, M. Cobalt-Catalyzed Hydroboration of Alkenes, Aldehydes, and Ketones. *Org. Lett.* **2018**, *20* (21), 6695–6700.

(8) Shirase, S.; Tamaki, S.; Shinohara, K.; Hirosawa, K.; Tsurugi, H.; Satoh, T.; Mashima, K. Cerium(IV) Carboxylate Photocatalyst for Catalytic Radical Formation from Carboxylic Acids: Decarboxylative Oxygenation of Aliphatic Carboxylic Acids and Lactonization of Aromatic Carboxylic Acids. *J. Am. Chem. Soc.* **2020**, *142* (12), 5668–5675.

7. NMR spectra of compounds

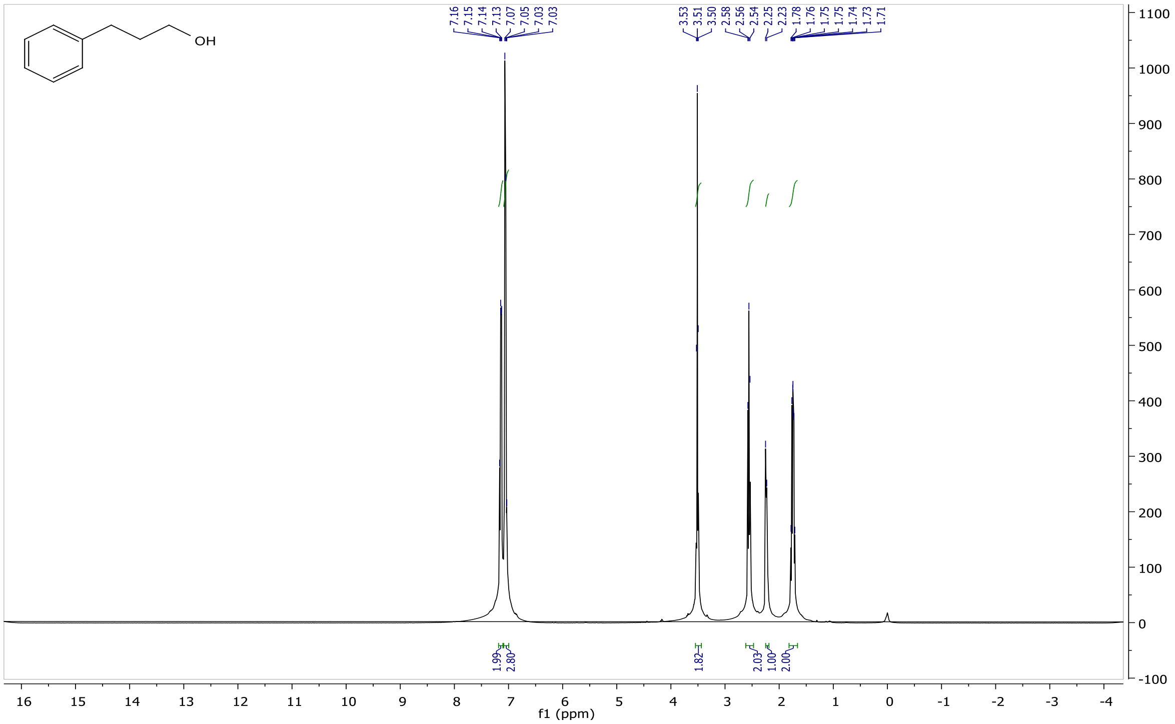

Figure S24. ^{1}H NMR spectrum of **2a** in $CDCl_3$ (400 MHz)

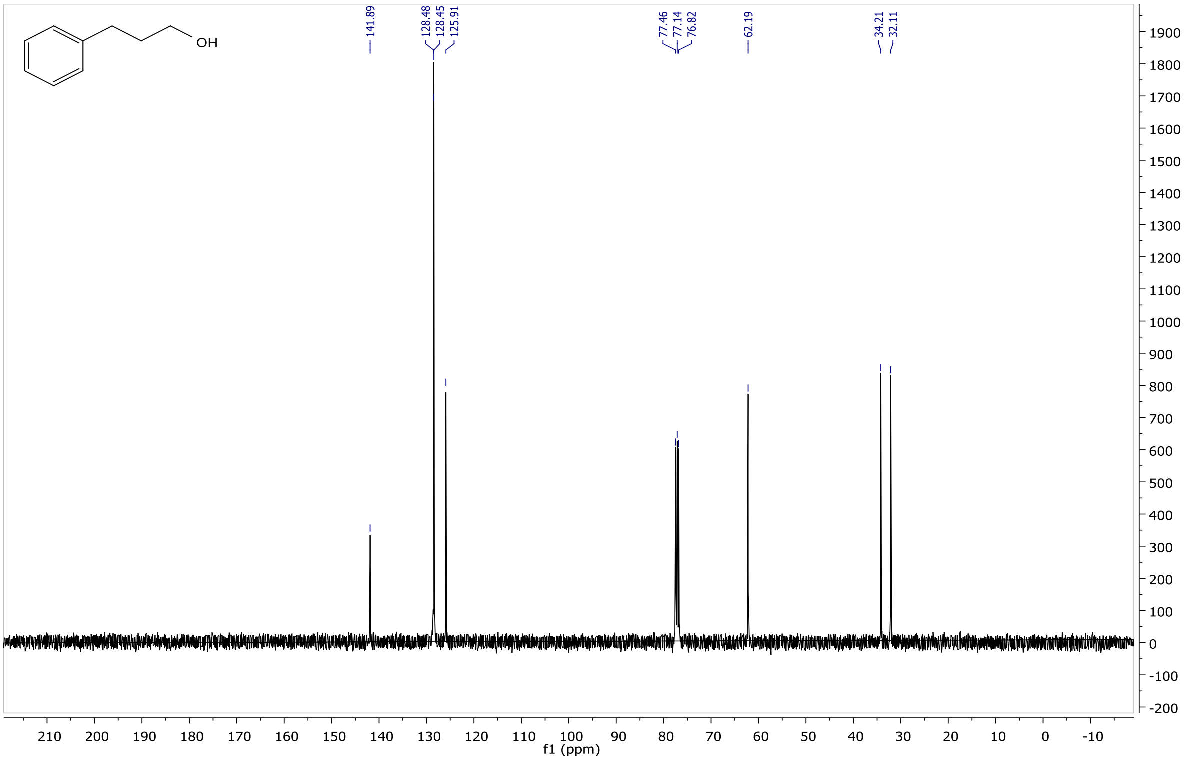

Figure S25. ^{13}C NMR spectrum of **2a** in $CDCl_3$ (101 MHz)

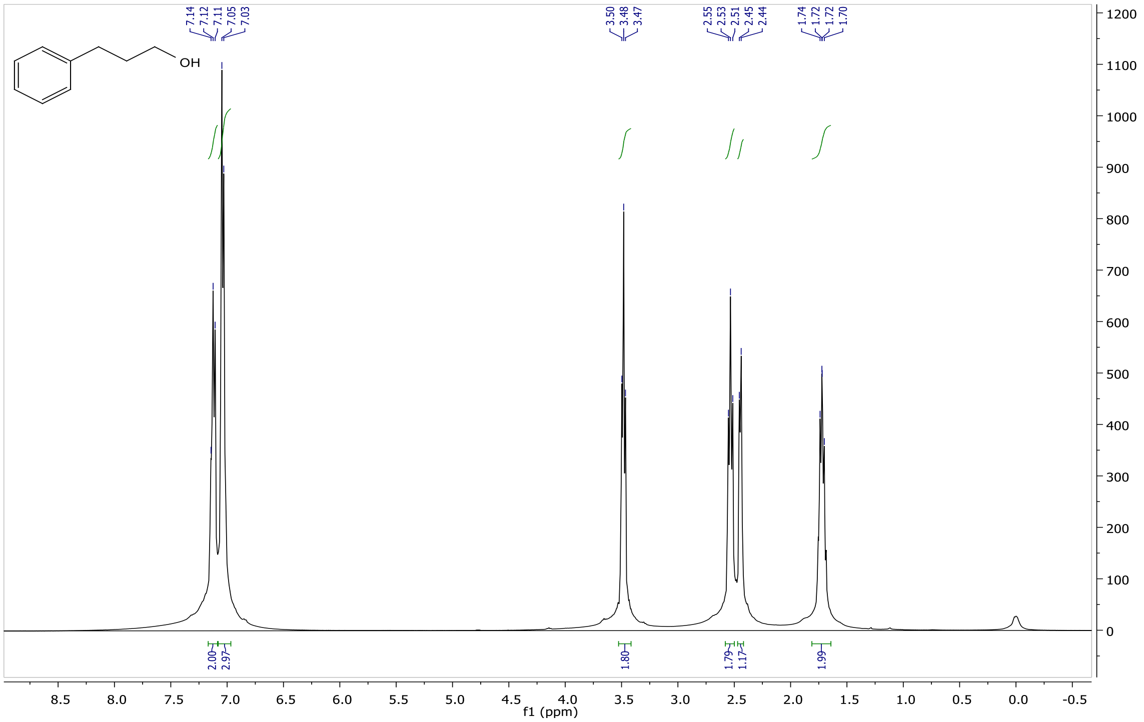

Figure S26. ^{1}H NMR spectrum of **2b** in $CDCl_3$ (400 MHz)

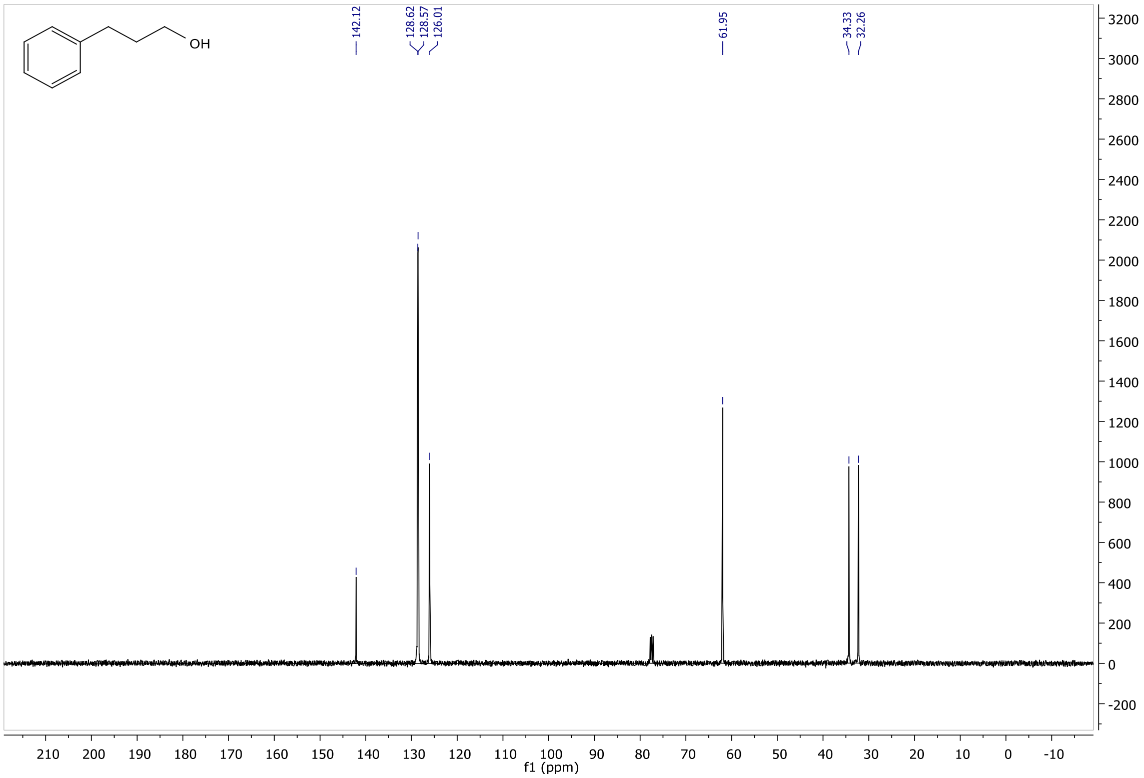

Figure S27. ^{13}C NMR spectrum of **2b** in $CDCl_3$ (101 MHz)

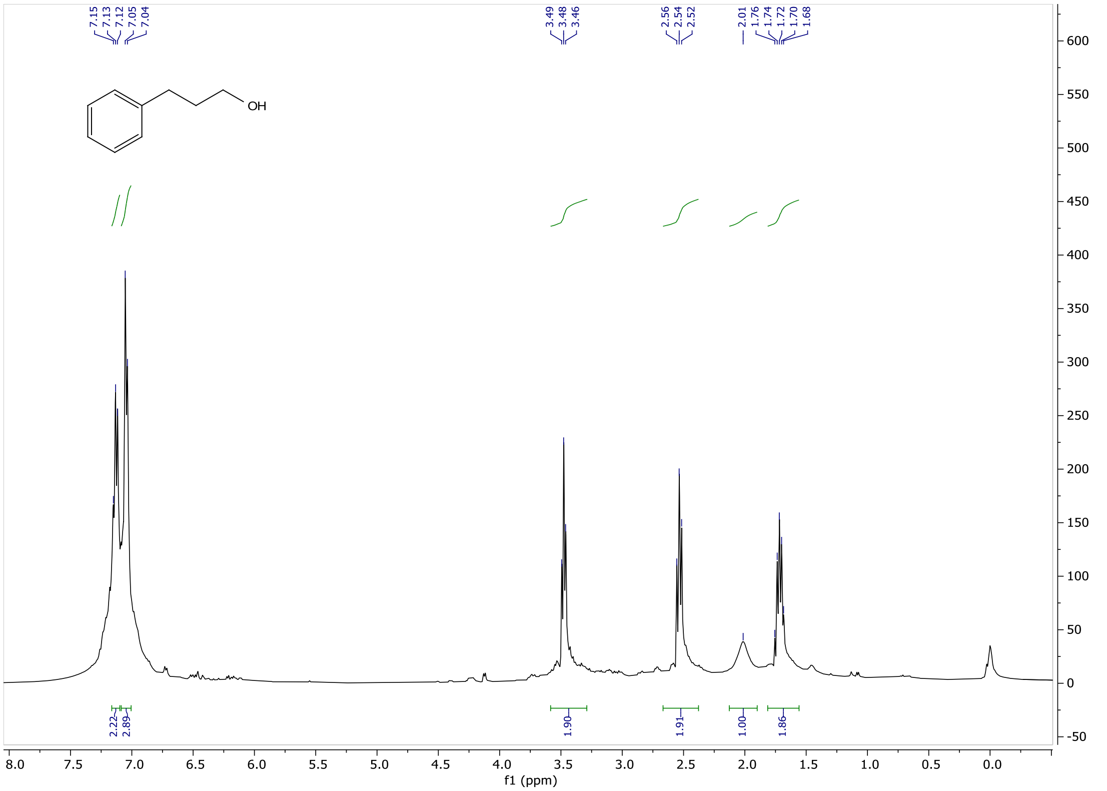

Figure S28. ^{1}H NMR spectrum of **2c** in $CDCl_3$ (400 MHz)

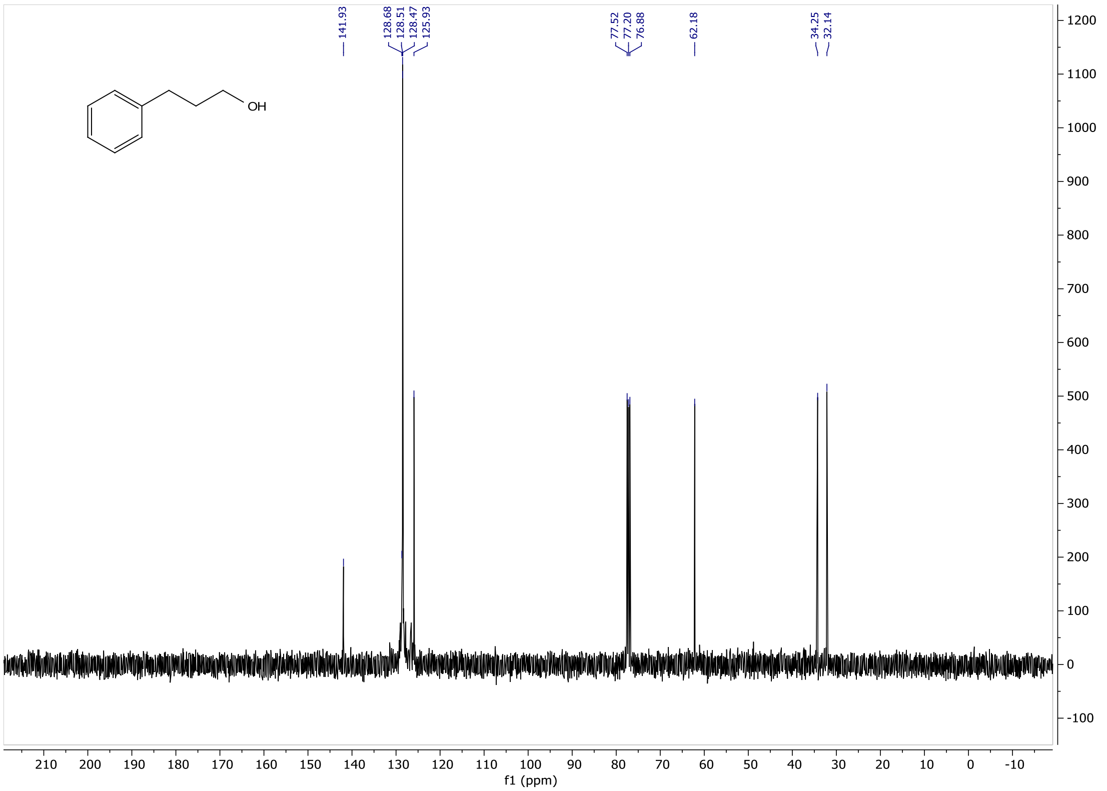

Figure S29. ^{13}C NMR spectrum of **2c** in $CDCl_3$ (101 MHz)

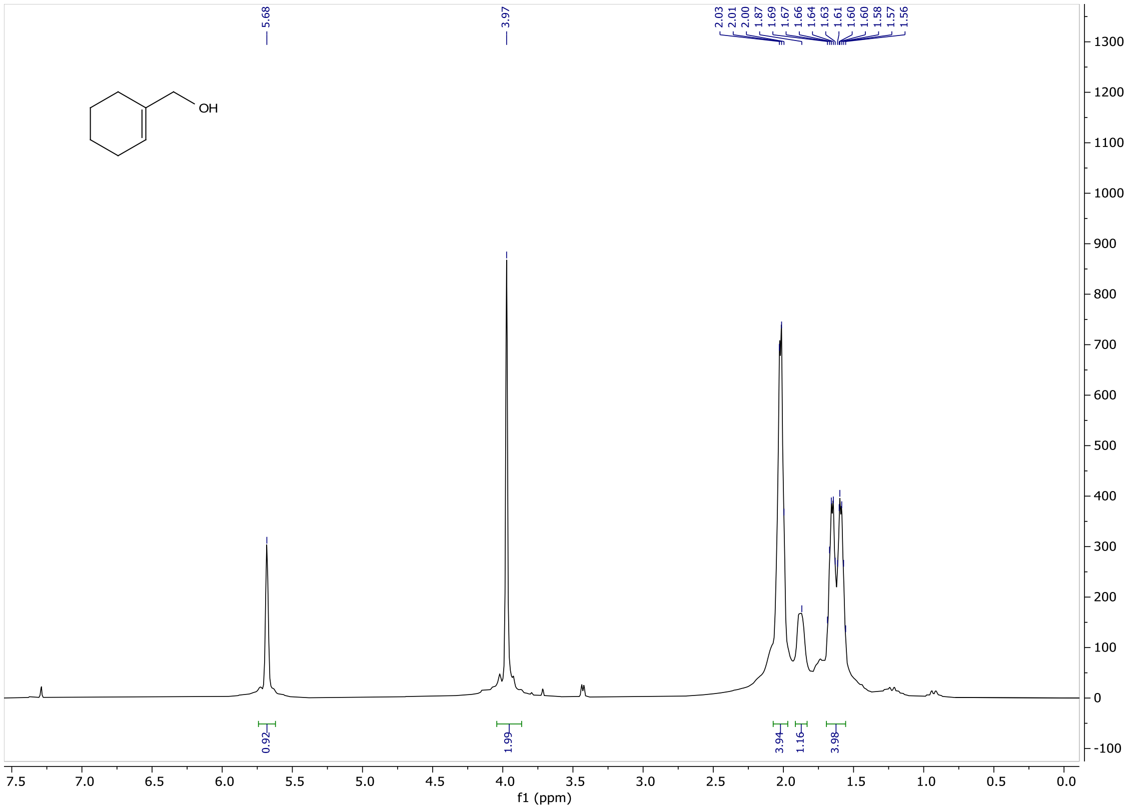

Figure S30. ^{1}H NMR spectrum of **2d** in $CDCl_3$ (400 MHz)

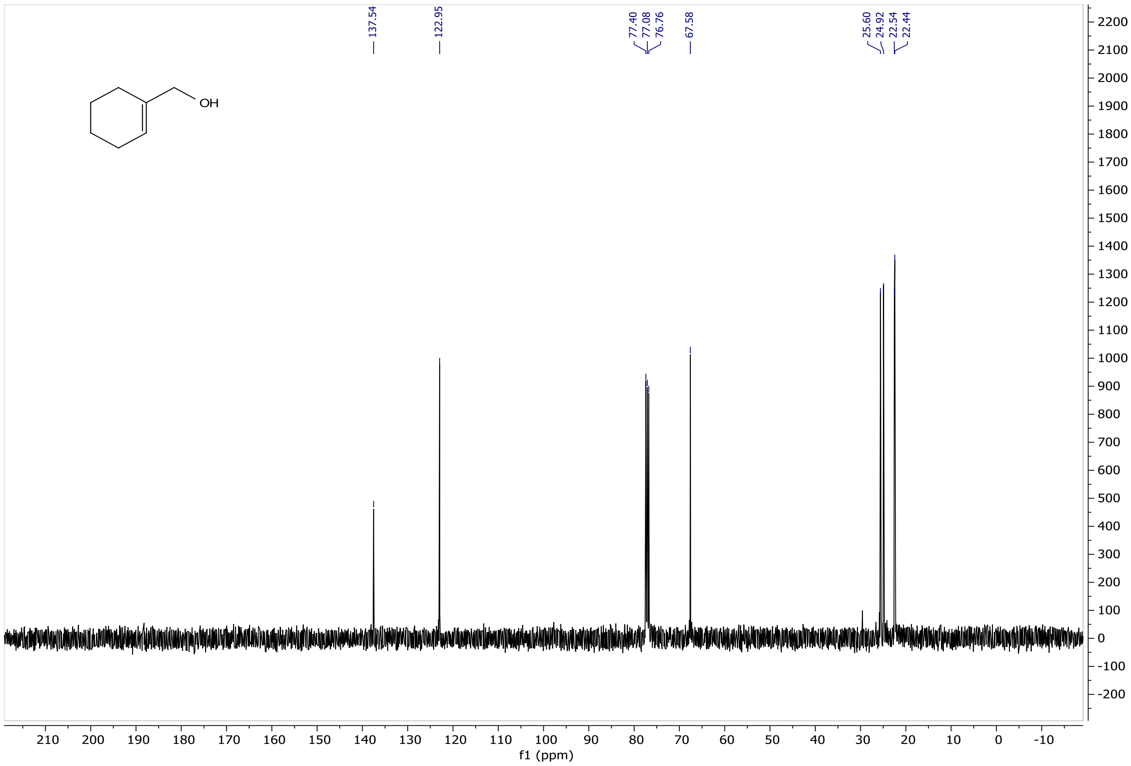

Figure S31. ^{13}C NMR spectrum of **2d** in $CDCl_3$ (101 MHz)

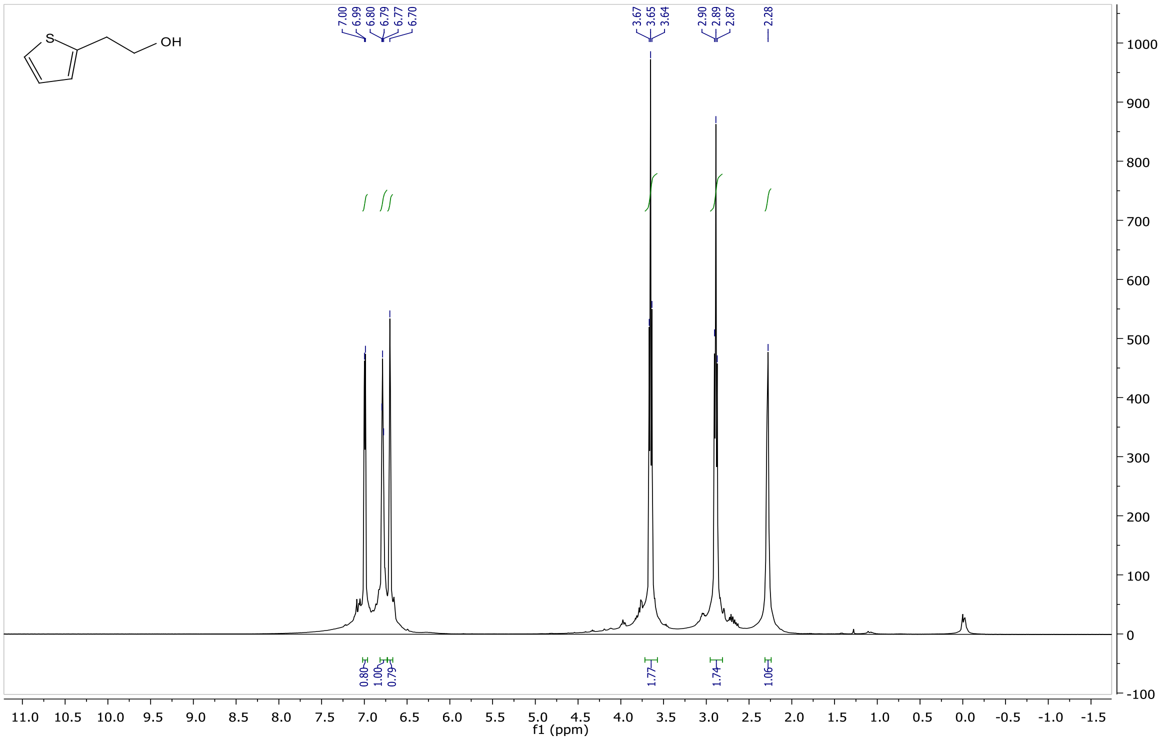

Figure S32. ^{1}H NMR spectrum of **2e** in $CDCl_3$ (400 MHz)

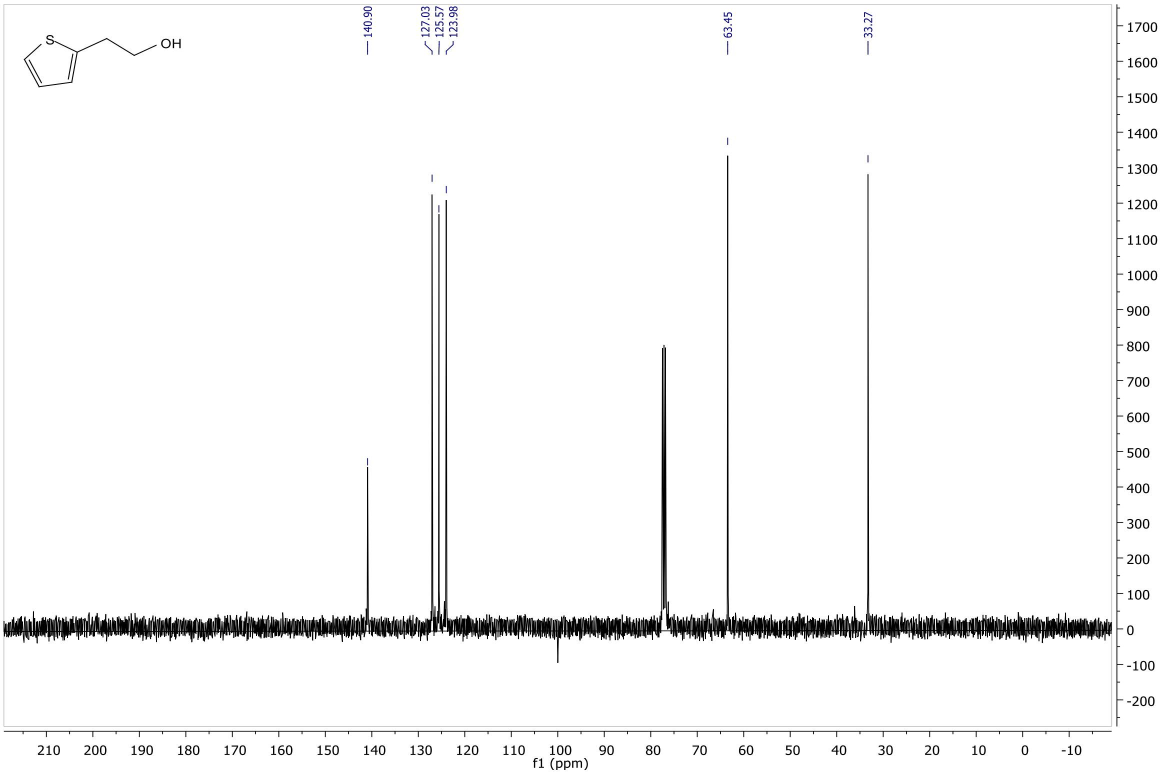

Figure S33. ^{13}C NMR spectrum of **2e** in $CDCl_3$ (101 MHz)

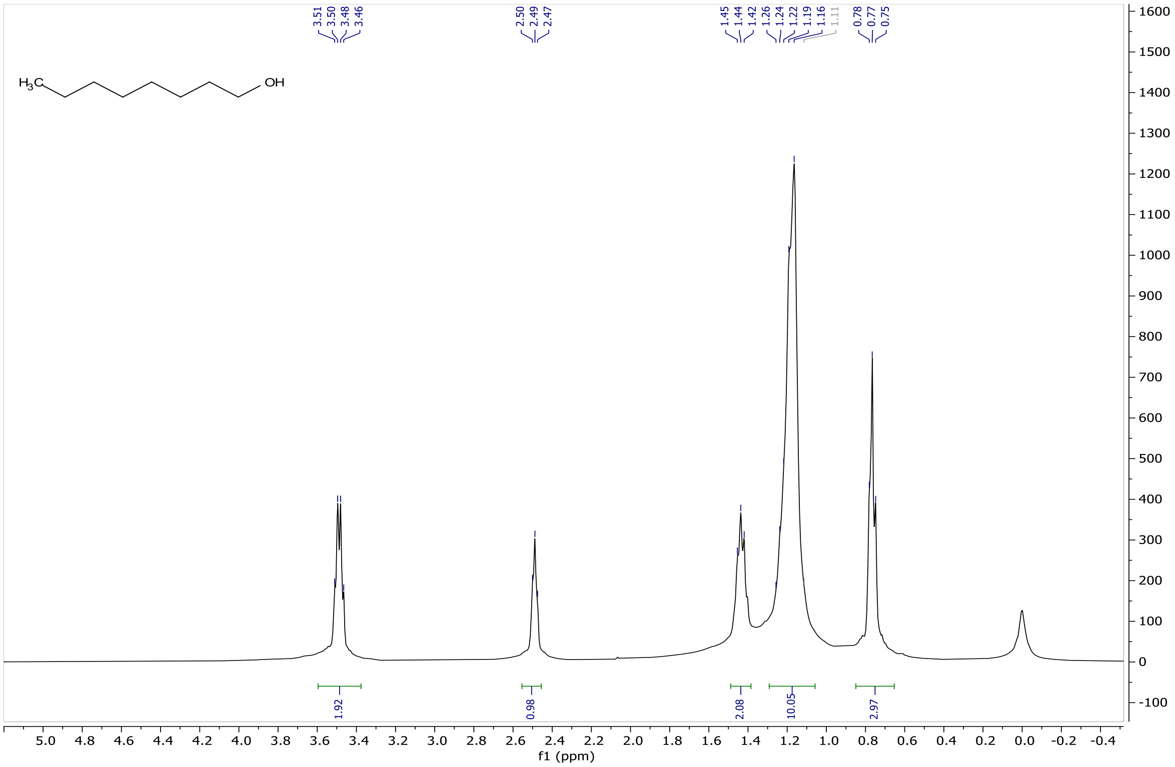

Figure S34. ^{1}H NMR spectrum of **2f** in $CDCl_3$ (400 MHz)

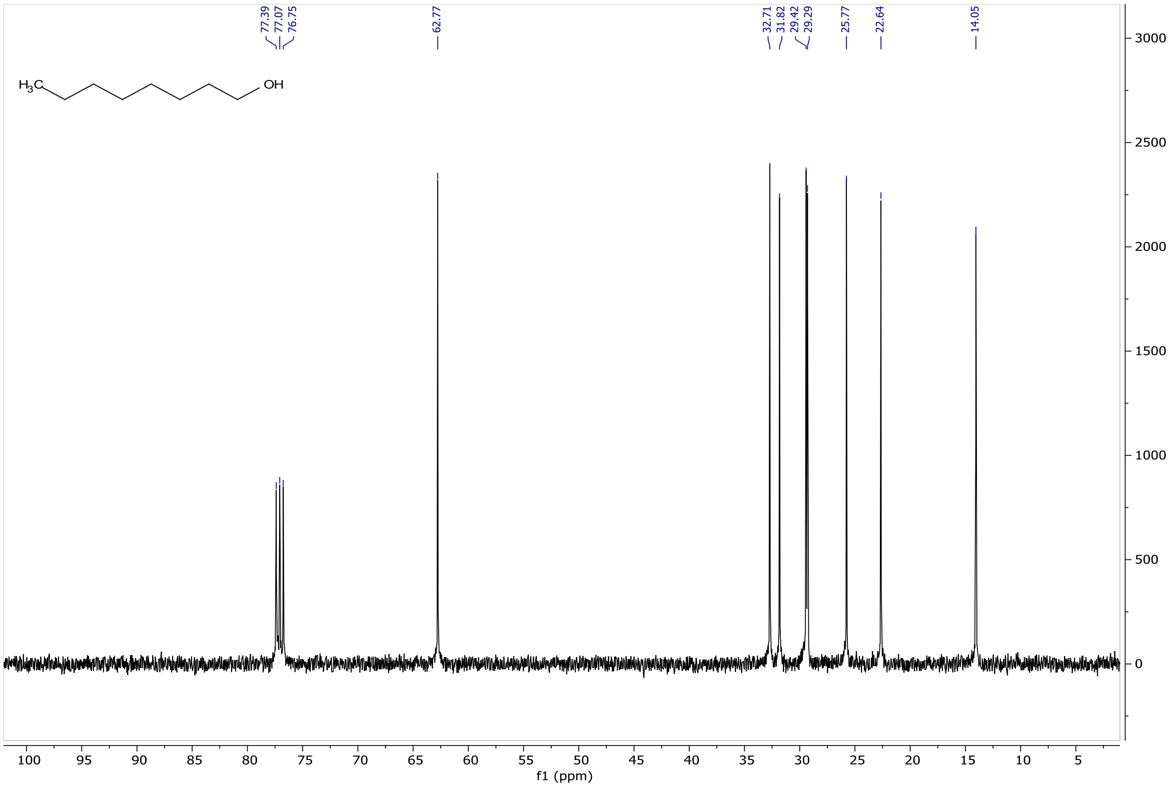

Figure S35. ^{13}C NMR spectrum of **2f** in $CDCl_3$ (101 MHz)

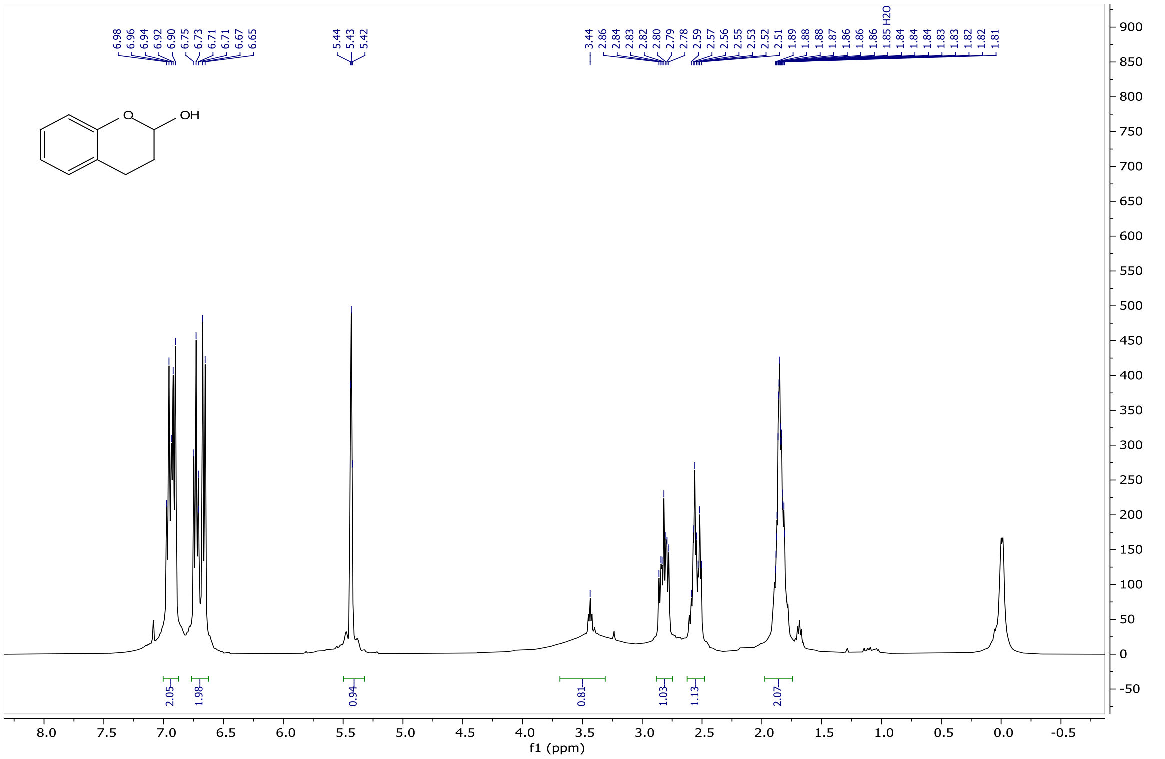

Figure S36. ^{1}H NMR spectrum of **2g** in $CDCl_3$ (400 MHz)

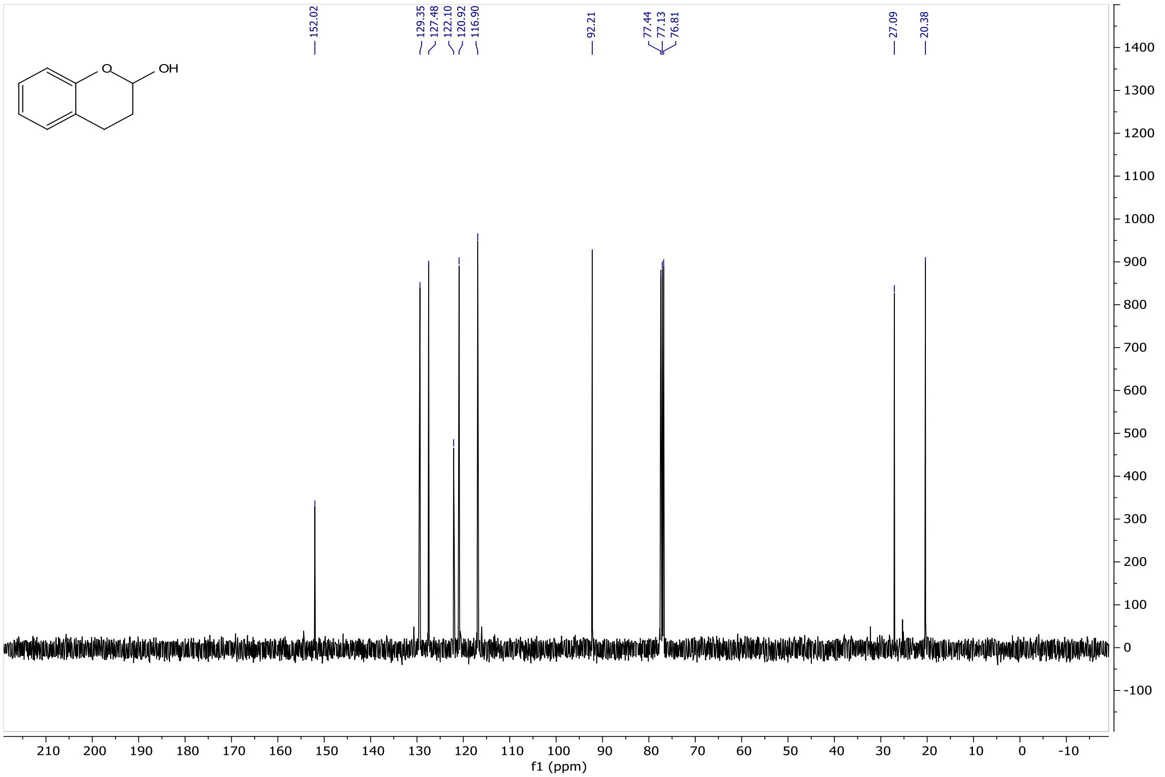

Figure S37. ^{13}C NMR spectrum of **2g** in $CDCl_3$ (101 MHz)

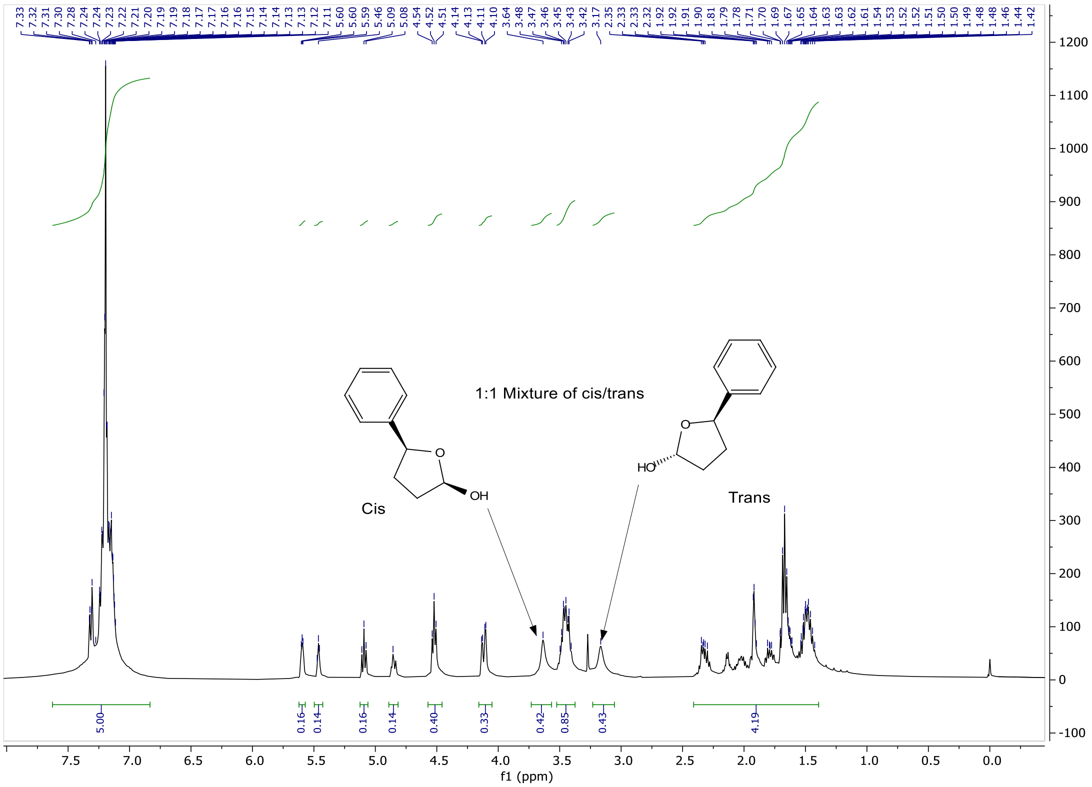

Figure S38. ^{1}H NMR spectrum of **2h** in $CDCl_3$ (400 MHz)

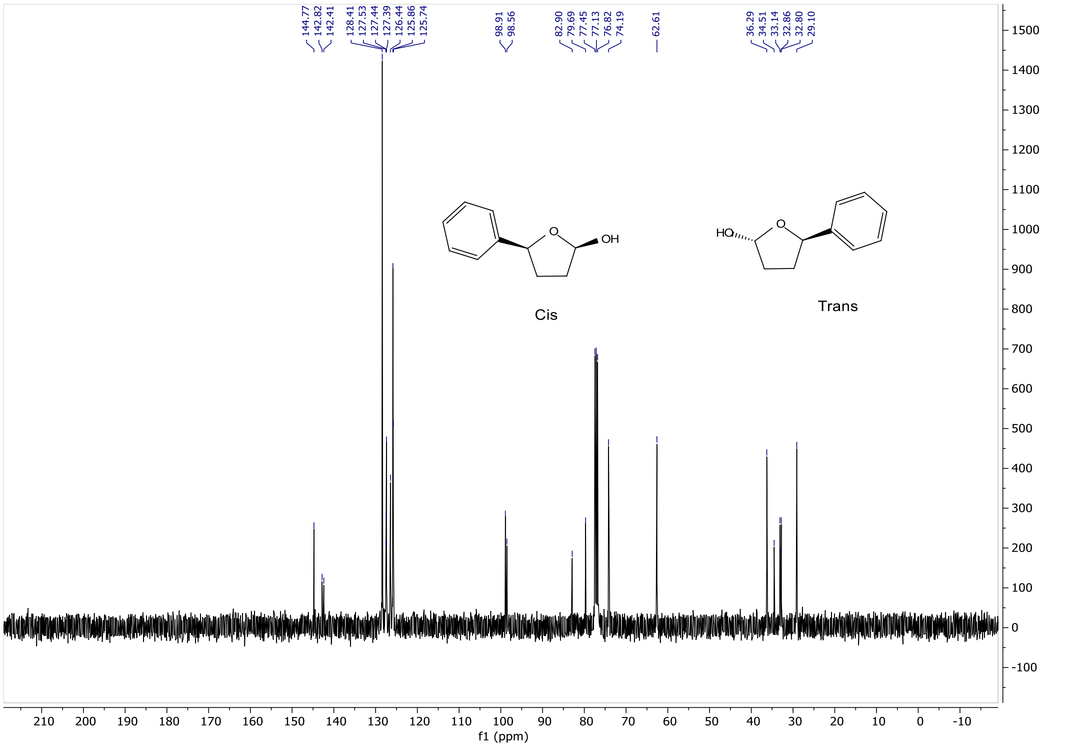

Figure S39. ^{13}C NMR spectrum of **2h** in $CDCl_3$ (101 MHz)

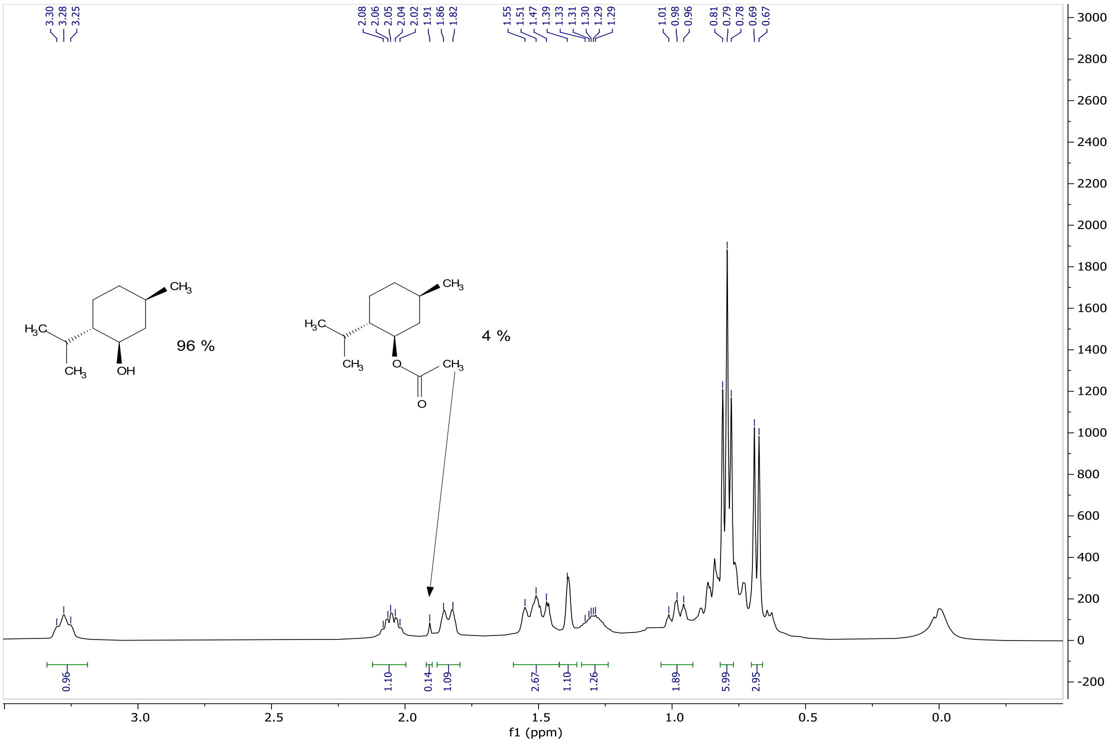

Figure S40. ^{1}H NMR spectrum of **2j** in $CDCl_3$ (400 MHz)

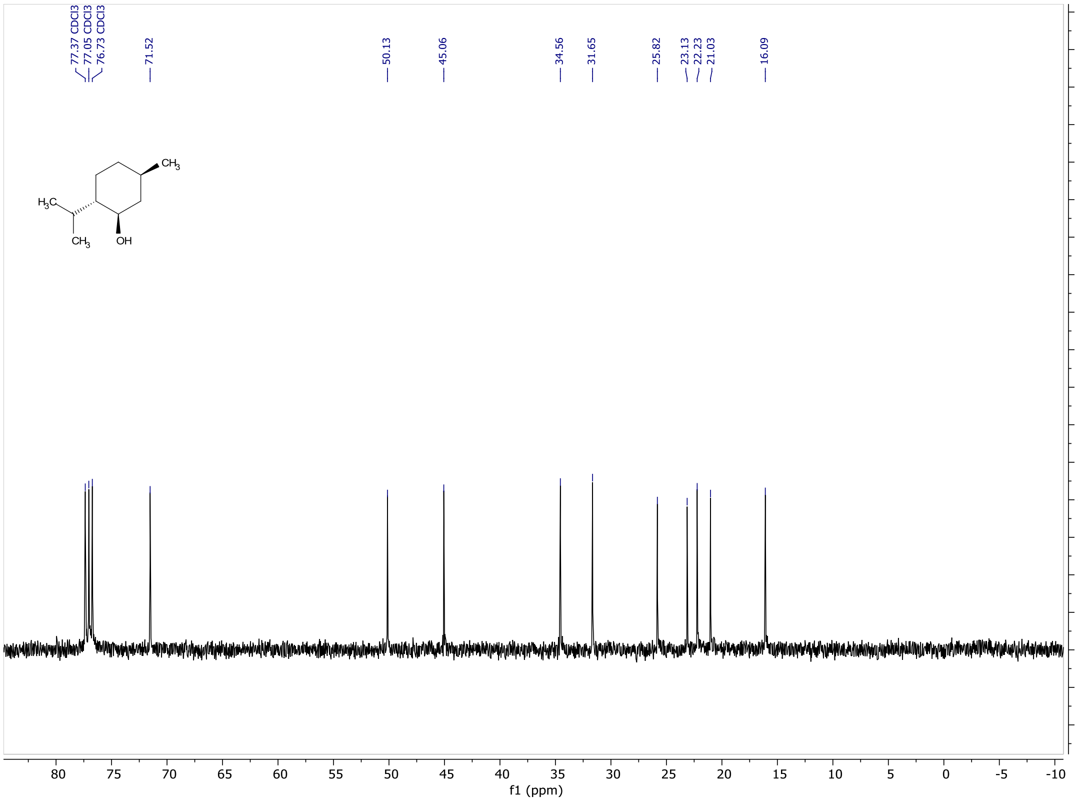

Figure S41. ^{13}C NMR spectrum of **2j** in $CDCl_3$ (101 MHz)

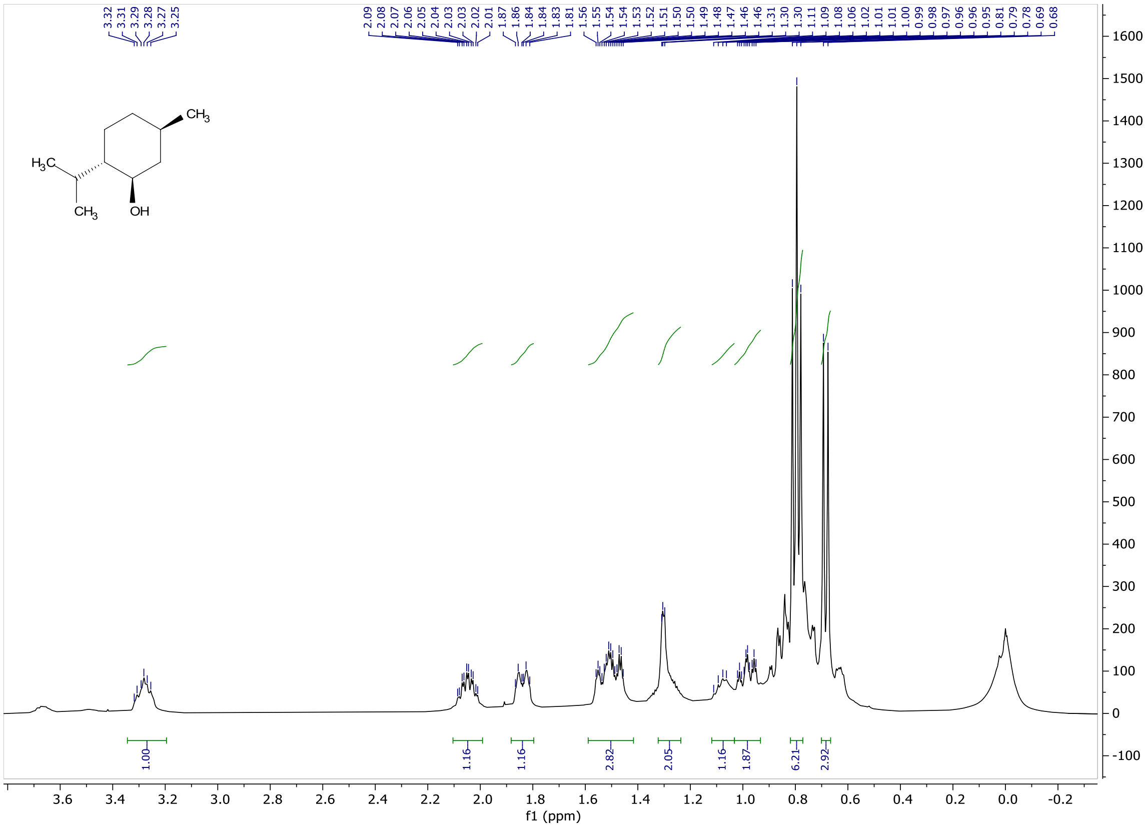

Figure S42. ^{1}H NMR spectrum of **2j** (isopropanol added) in $CDCl_3$ (400 MHz)

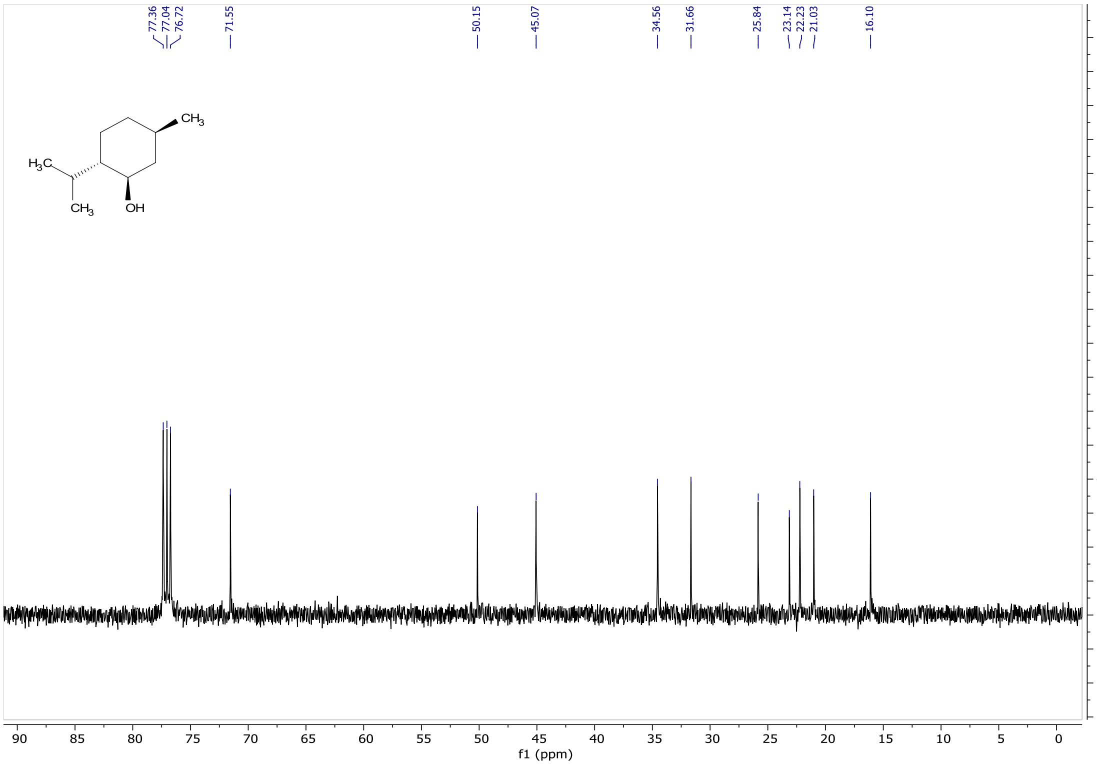

Figure S43. ^{13}C NMR spectrum of **2j** (isopropanol added) in $CDCl_3$ (101 MHz)

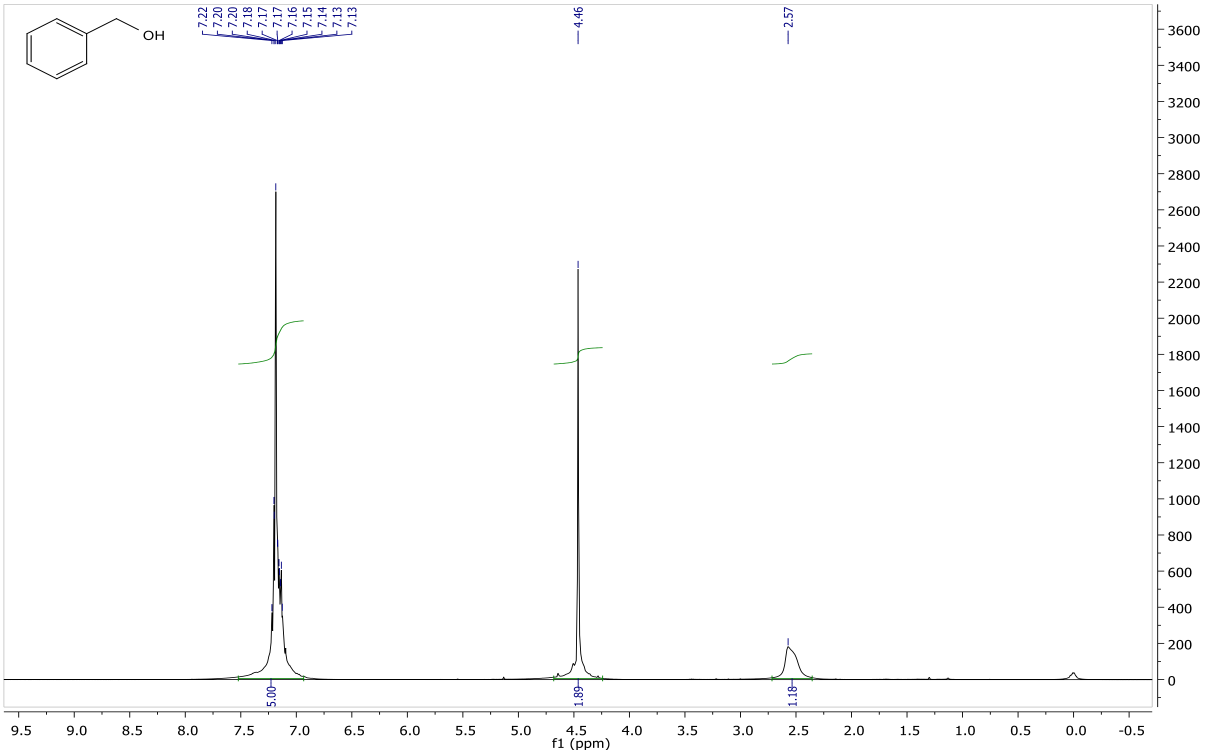

Figure S44. ^{1}H NMR spectrum of **4a** in $CDCl_3$ (400 MHz)

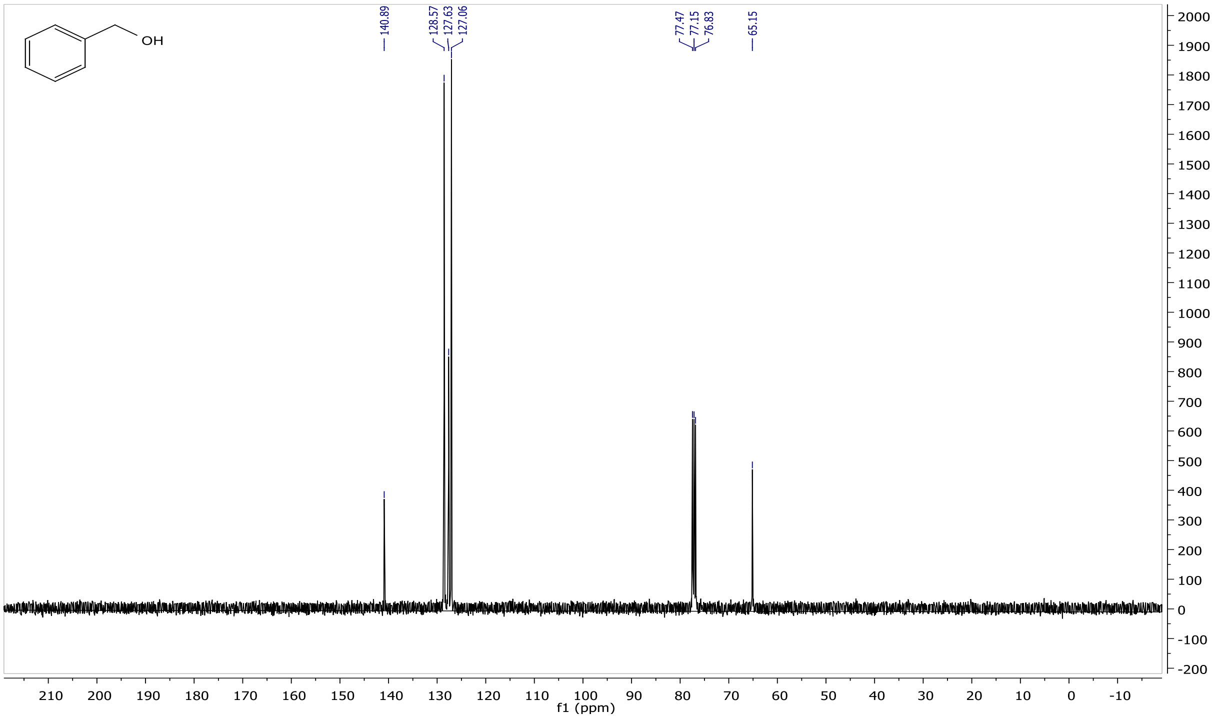

Figure S45. ^{13}C NMR spectrum of **4a** in $CDCl_3$ (101 MHz)

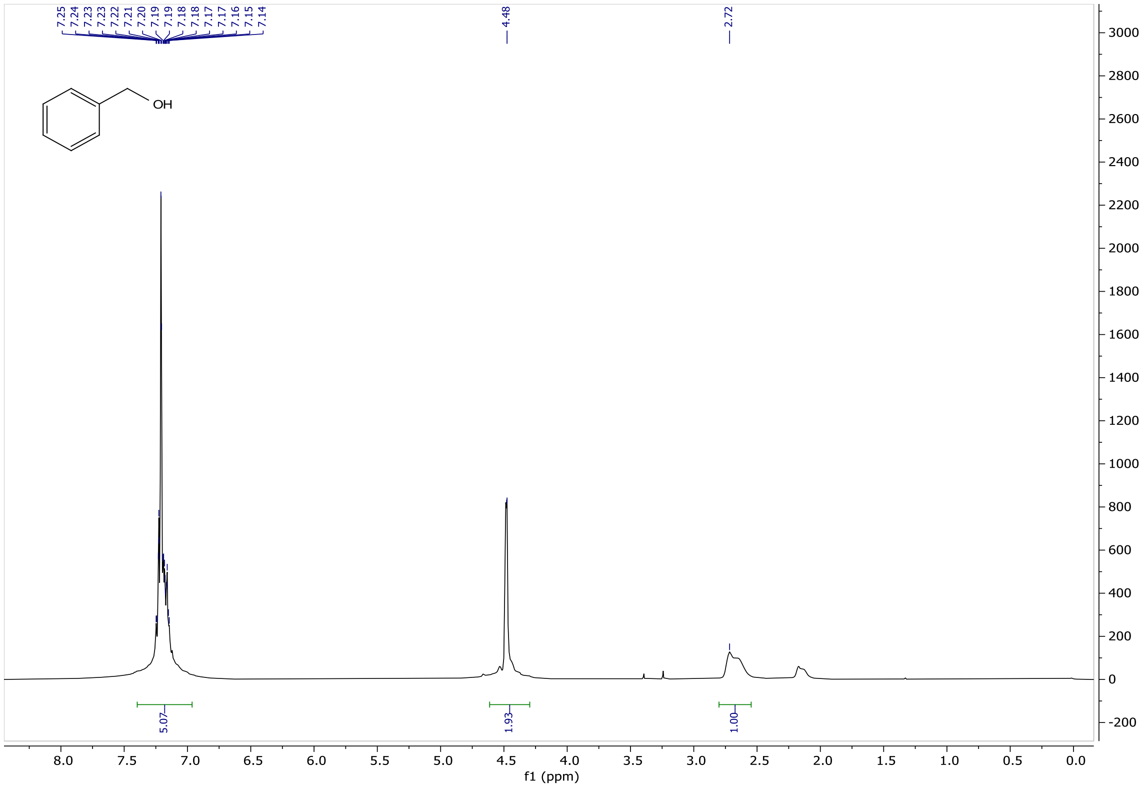

Figure S46. ^{1}H NMR spectrum of **4b** in $CDCl_3$ (400 MHz)

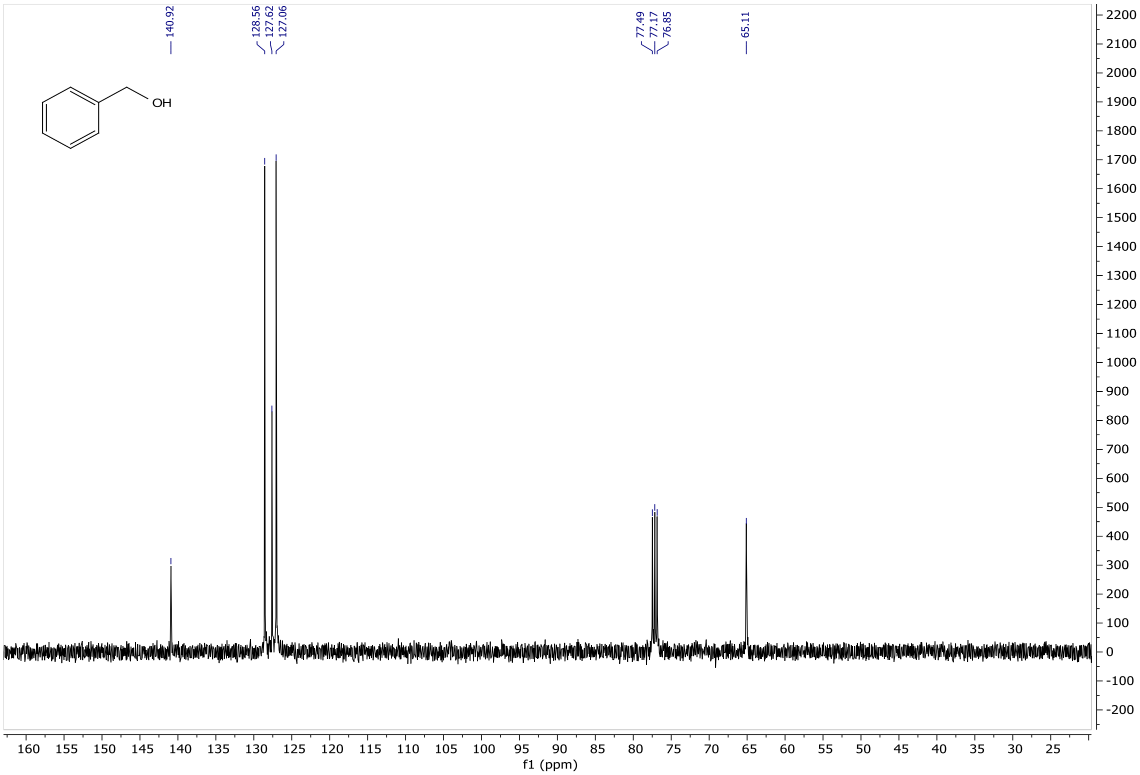

Figure S47. ^{13}C NMR spectrum of **4b** in $CDCl_3$ (101 MHz)

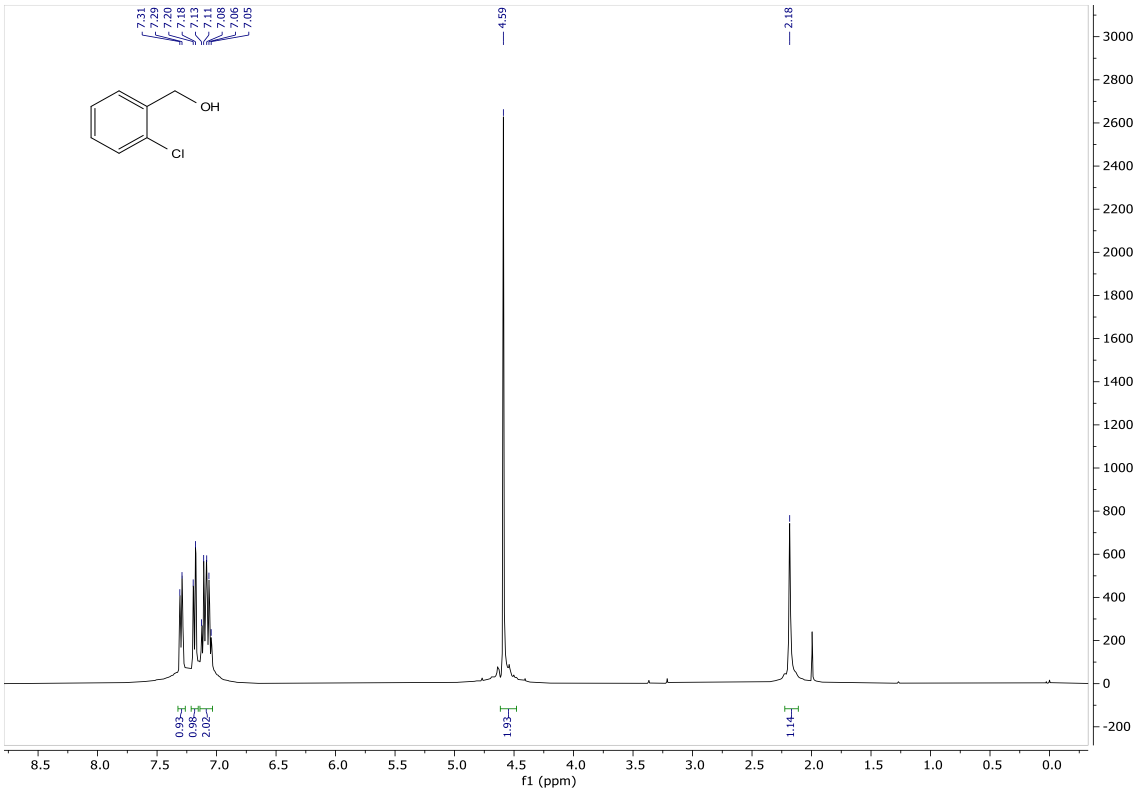

Figure S48. ^{1}H NMR spectrum of **4c** in $CDCl_3$ (400 MHz)

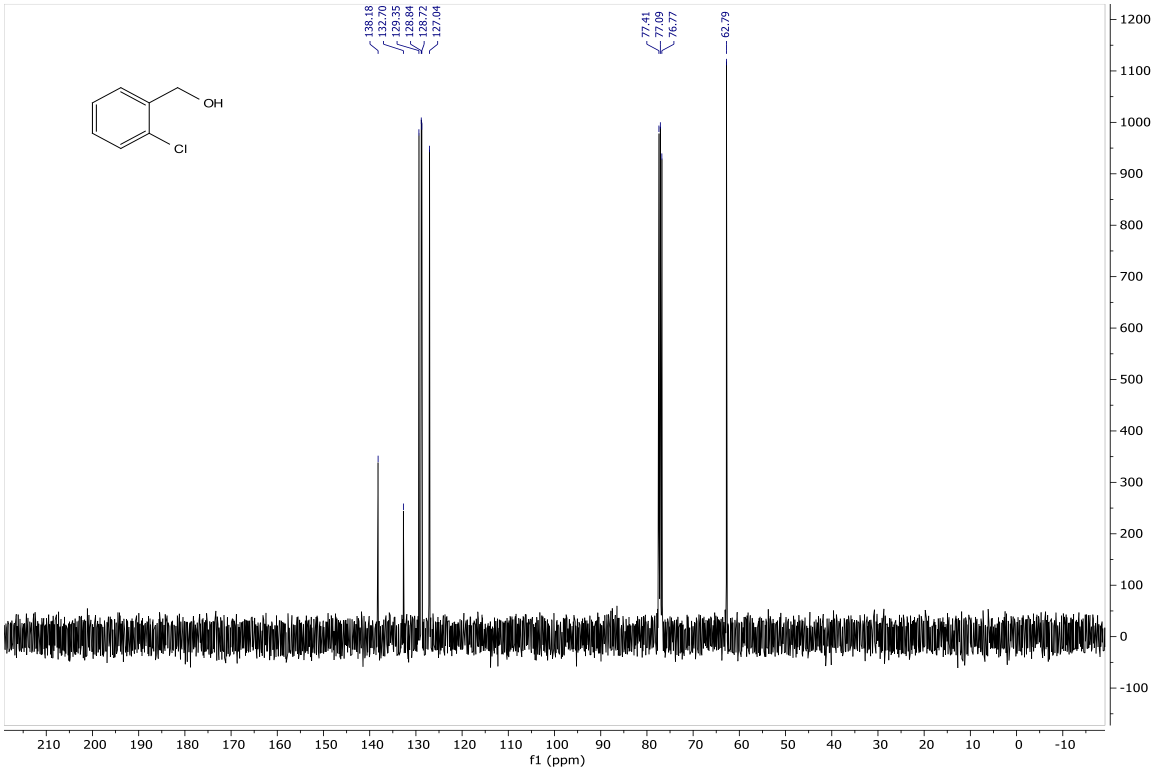

Figure S49. ^{13}C NMR spectrum of **4c** in $CDCl_3$ (101 MHz)

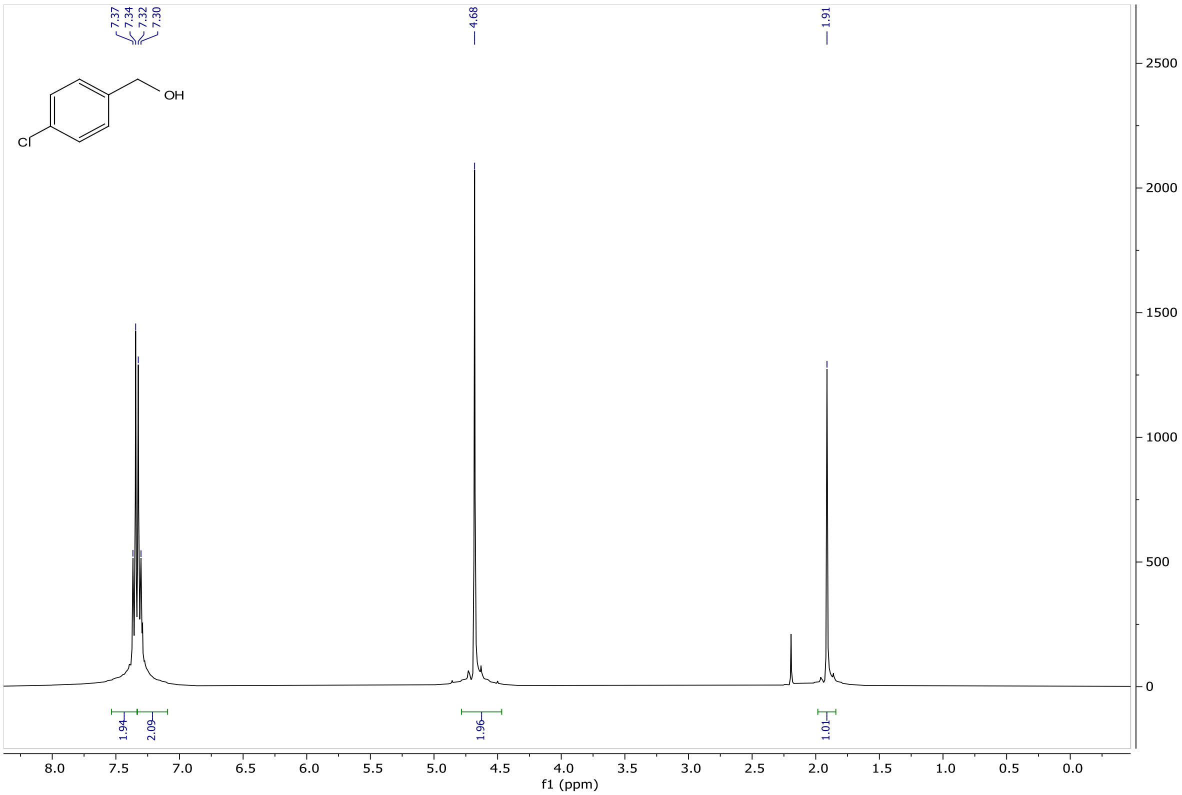

Figure S50. ^{1}H NMR spectrum of **4d** in $CDCl_3$ (400 MHz)

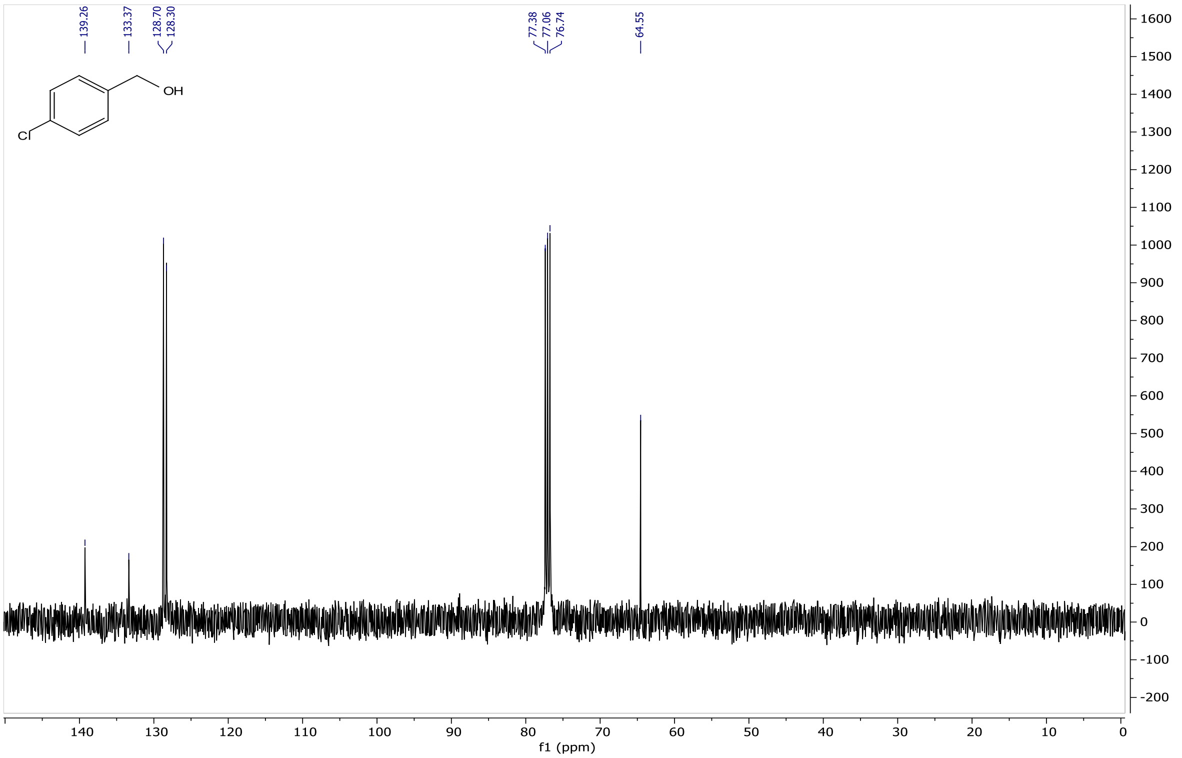

Figure S51. ^{13}C NMR spectrum of **4d** in $CDCl_3$ (101 MHz)

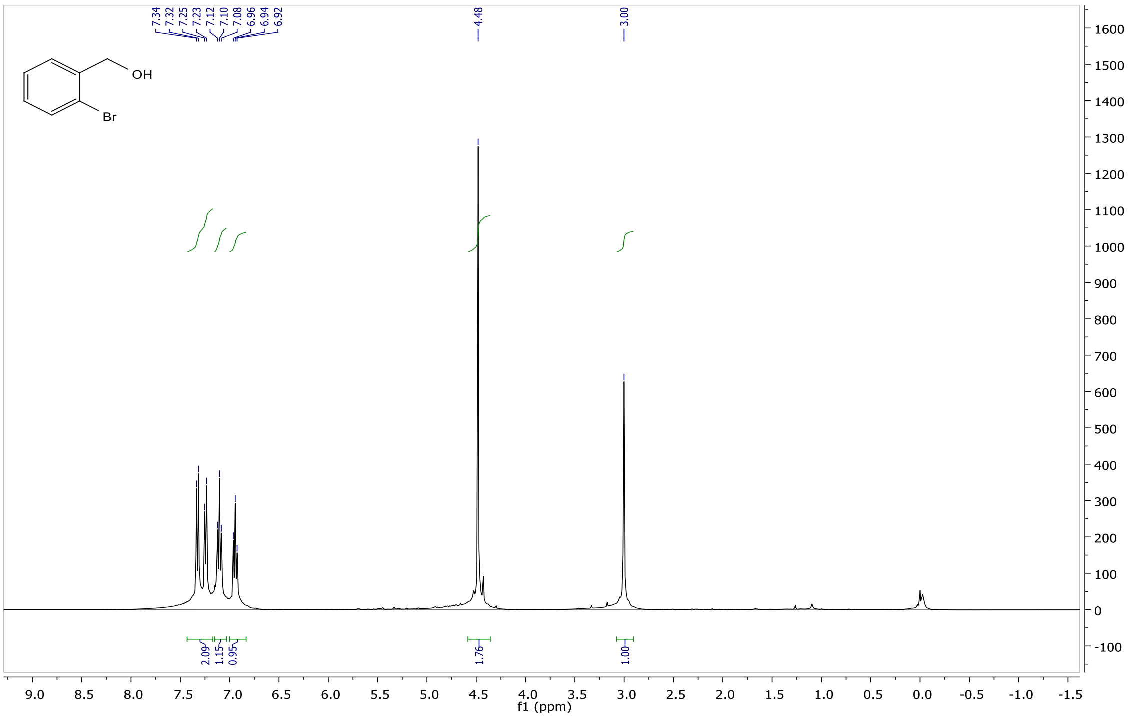

Figure S52. ^{1}H NMR spectrum of **4e** in $CDCl_3$ (400 MHz)

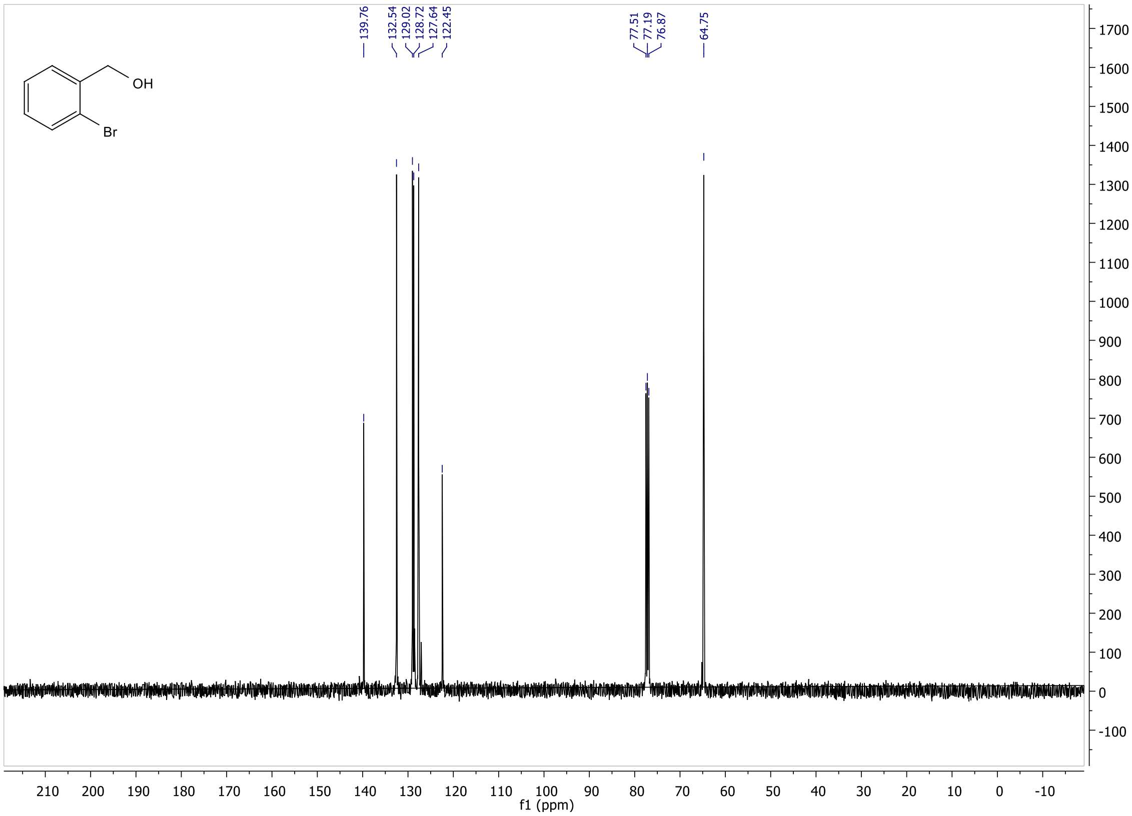

Figure S53. ^{13}C NMR spectrum of **4e** in $CDCl_3$ (101 MHz)

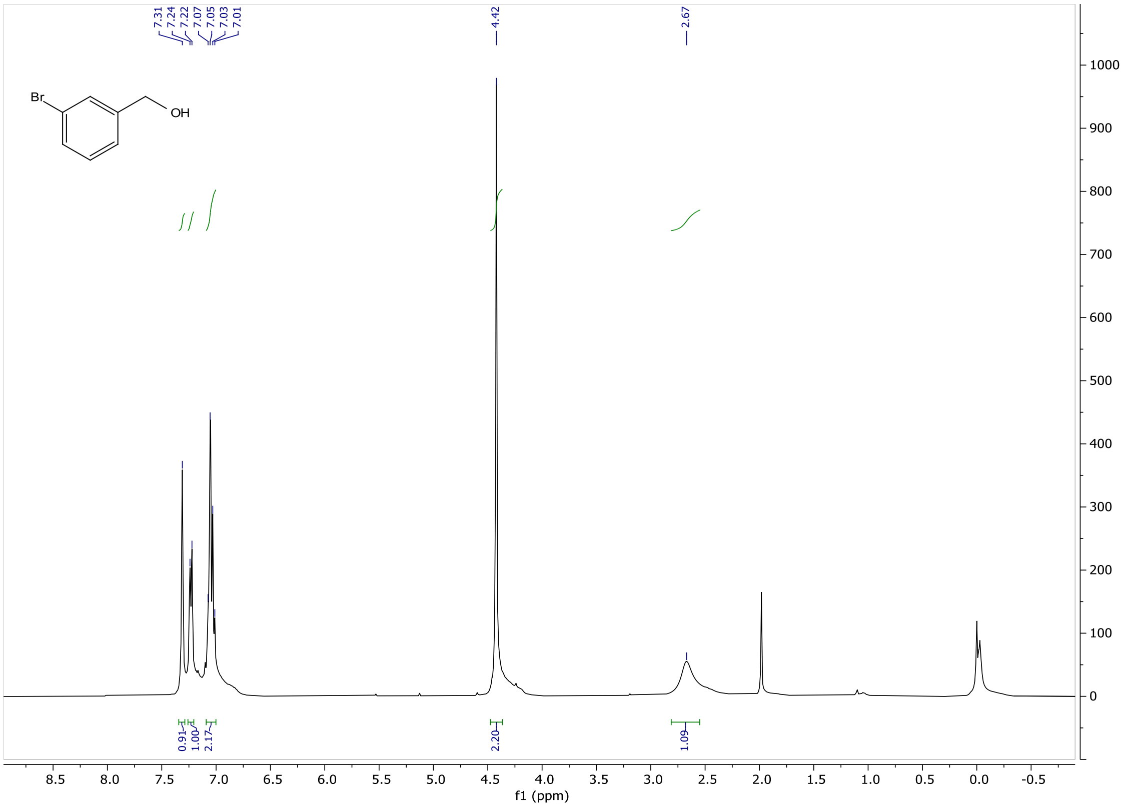

Figure S54. ^{1}H NMR spectrum of **4f** in $CDCl_3$ (400 MHz)

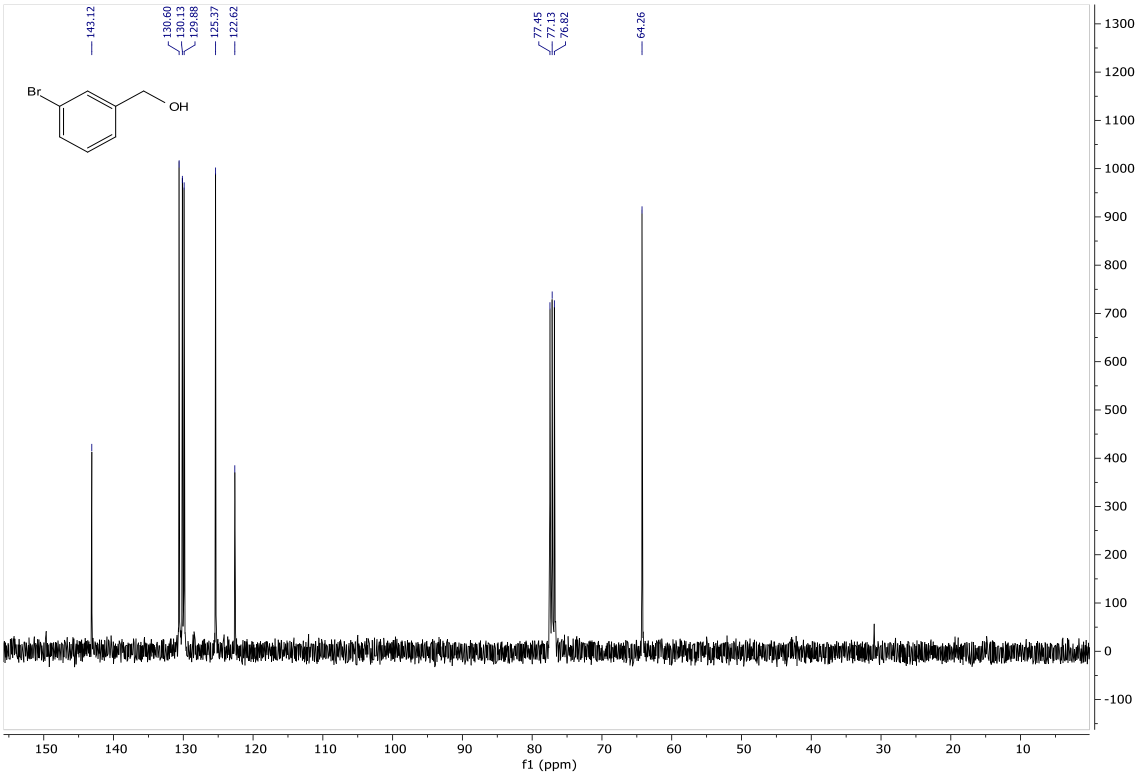

Figure S55. ^{13}C NMR spectrum of **4f** in $CDCl_3$ (101 MHz)

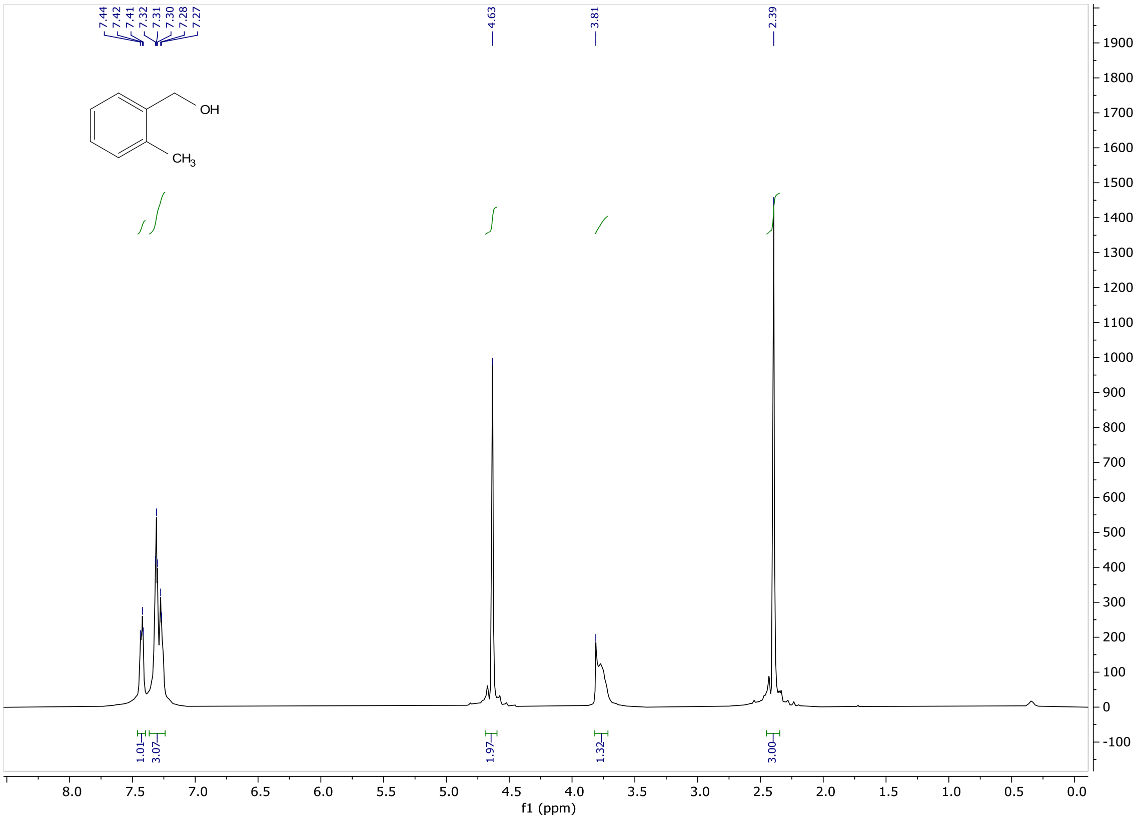

Figure S56. ^{1}H NMR spectrum of **4h** in $CDCl_3$ (400 MHz)

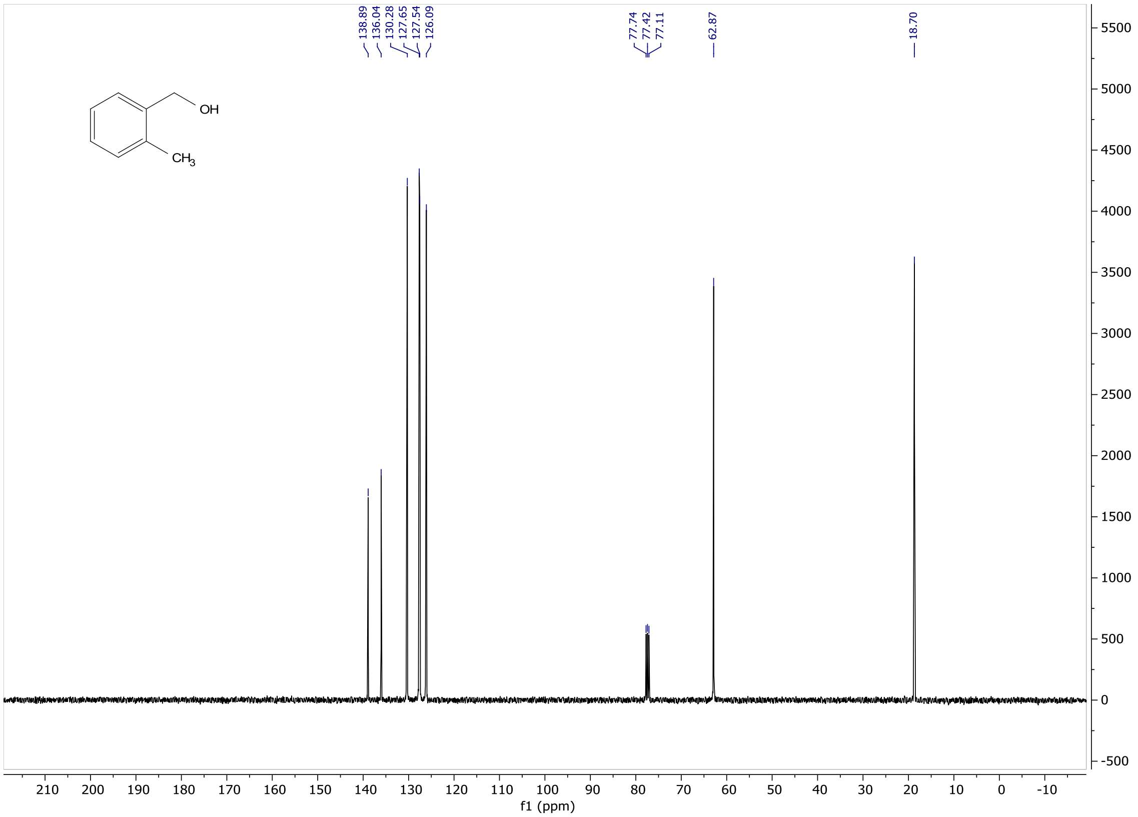

Figure S57. ^{13}C NMR spectrum of **4h** in $CDCl_3$ (101 MHz)

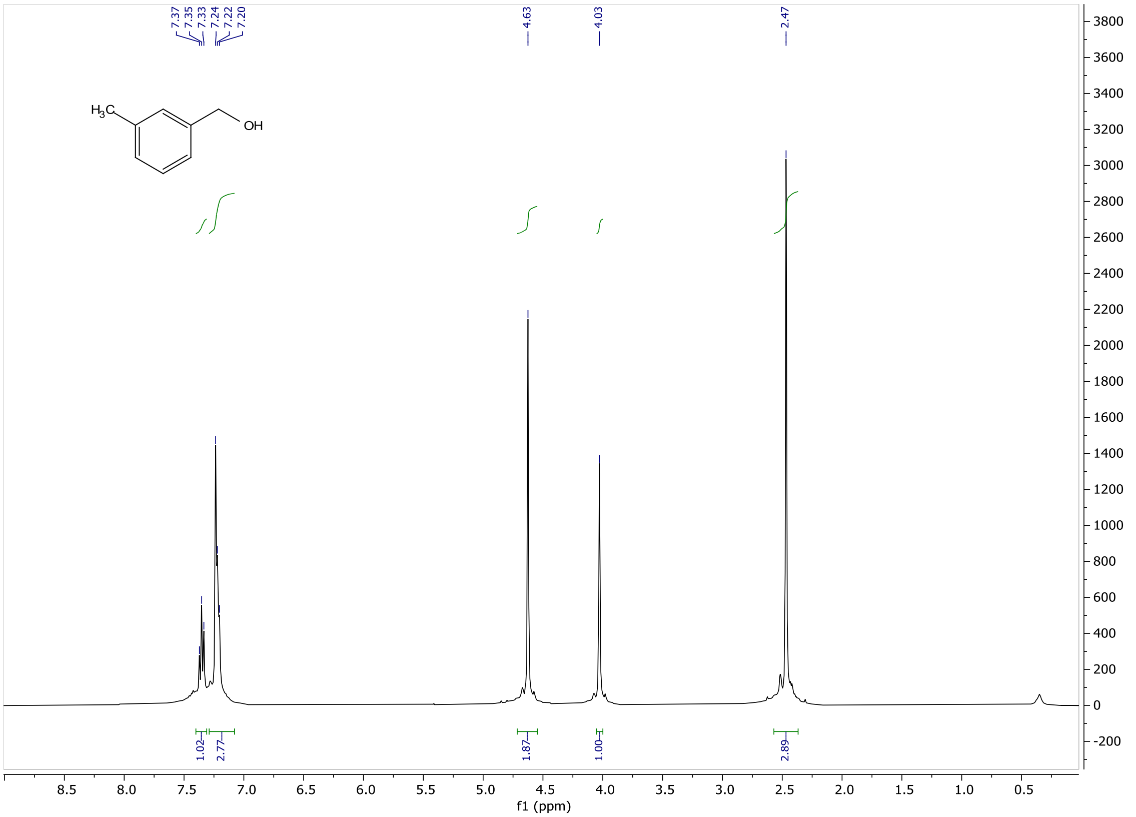

Figure S58. ^{1}H NMR spectrum of **4i** in $CDCl_3$ (400 MHz)

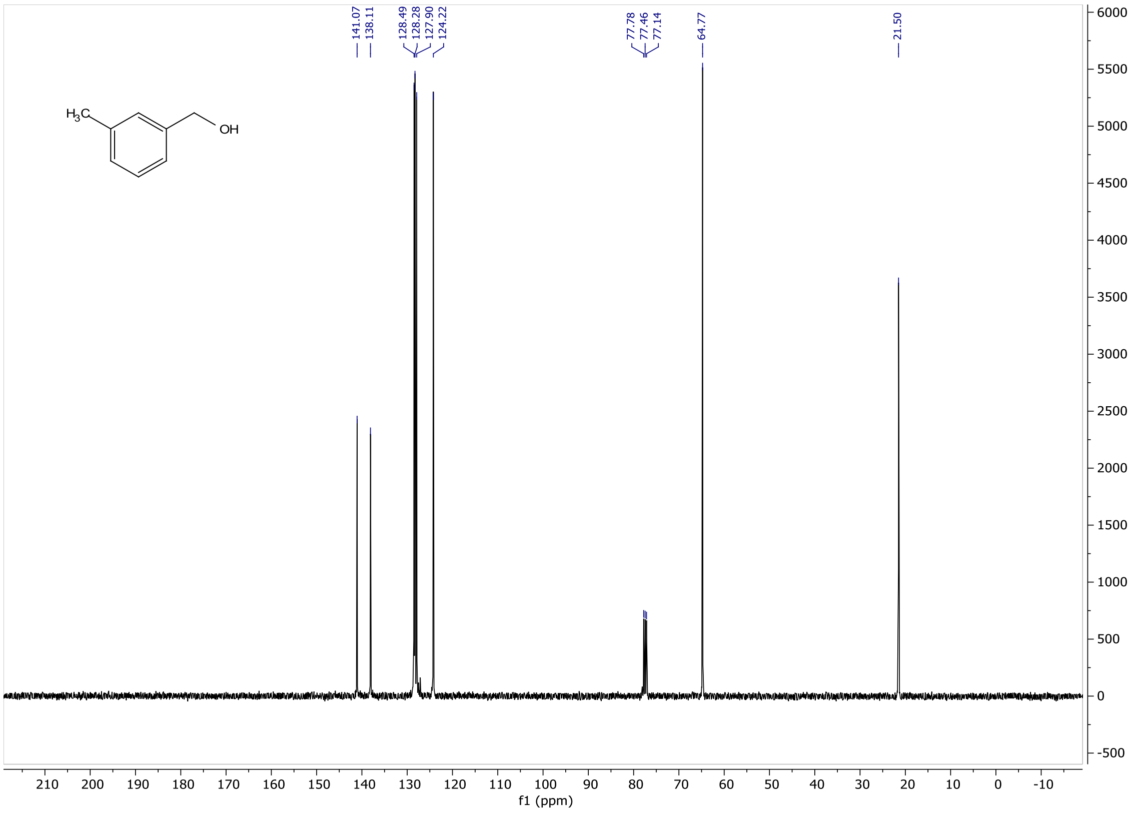

Figure S59. ^{13}C NMR spectrum of **4i** in $CDCl_3$ (101 MHz)

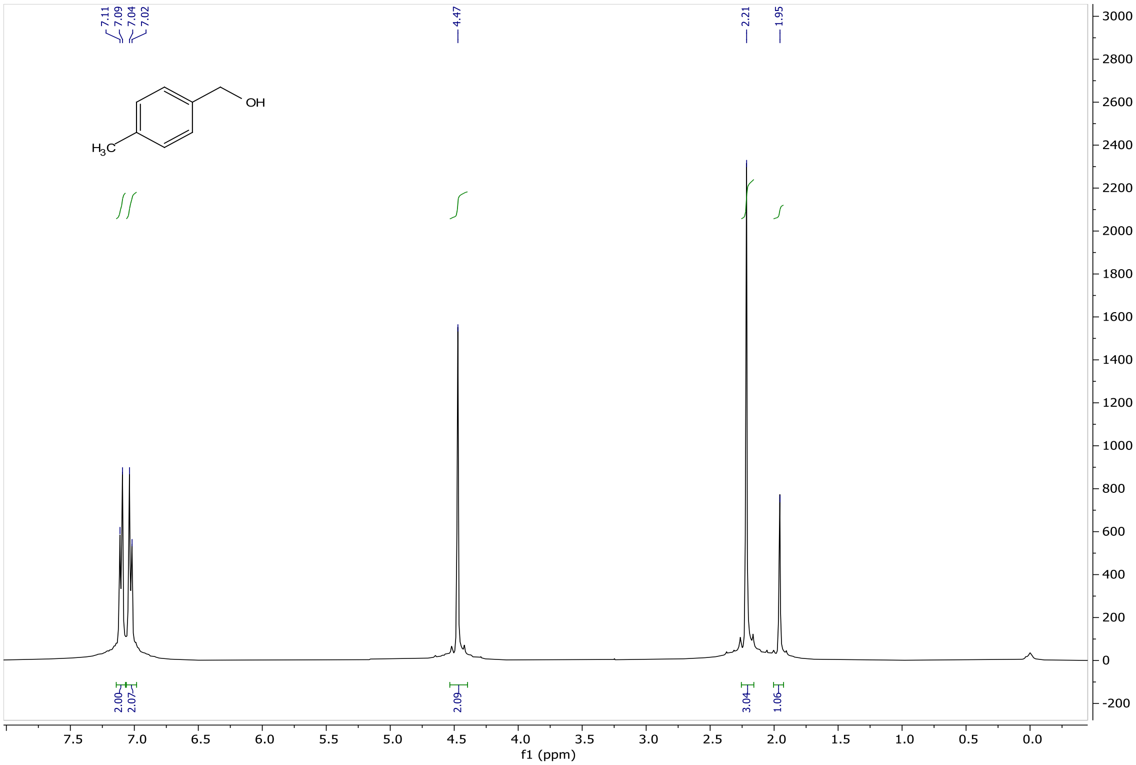

Figure S60. ^{1}H NMR spectrum of **4j** in $CDCl_3$ (400 MHz)

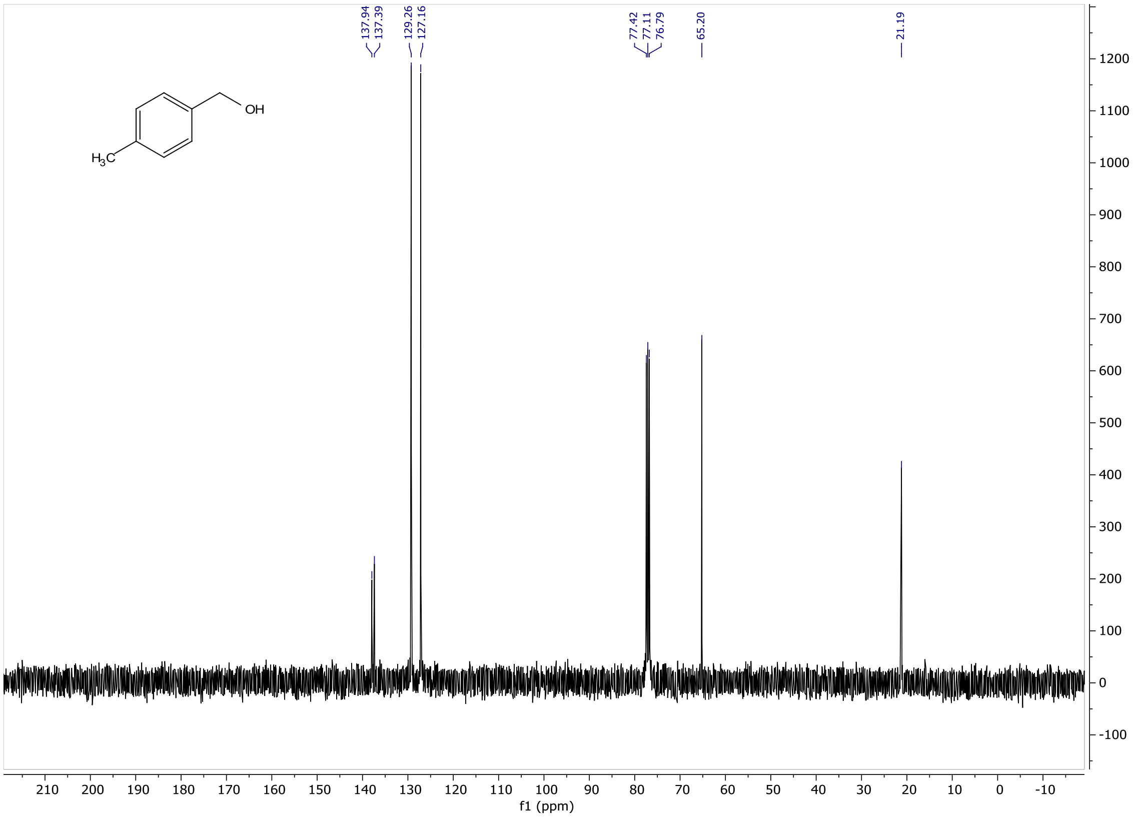

Figure S61. ^{13}C NMR spectrum of **4j** in $CDCl_3$ (101 MHz)

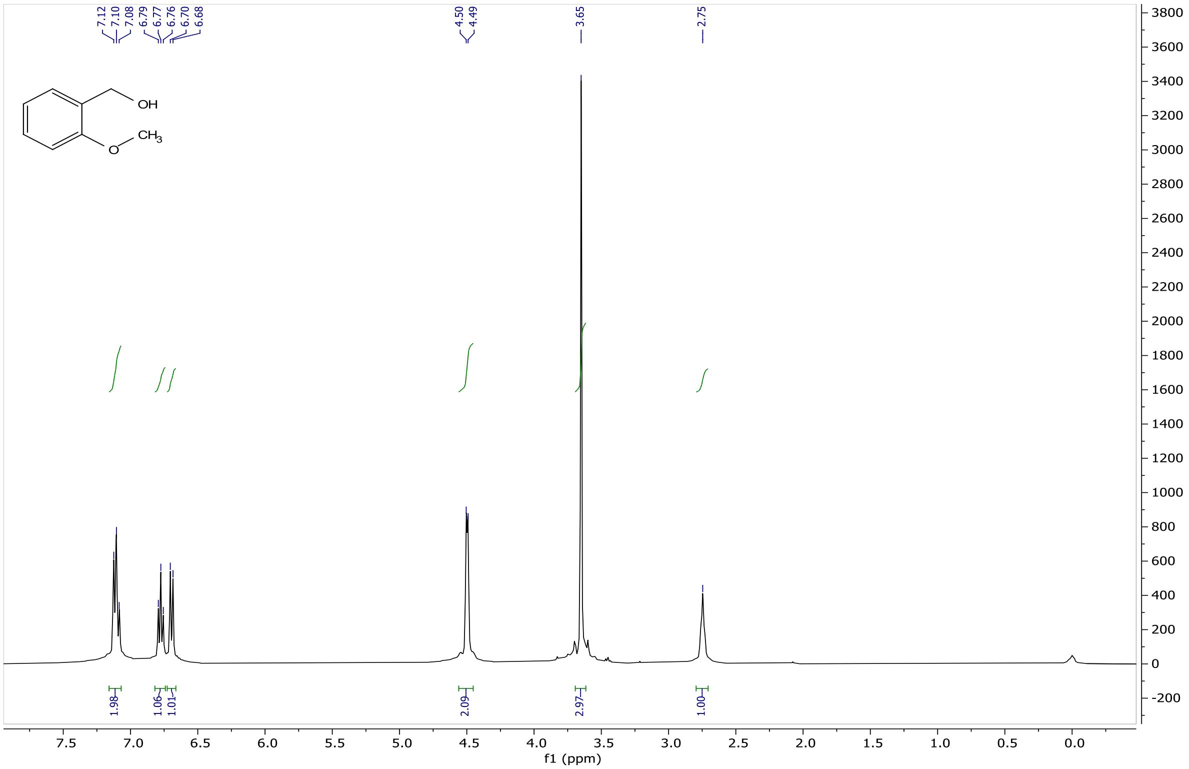

Figure S62. ^{1}H NMR spectrum of **4k** in $CDCl_3$ (400 MHz)

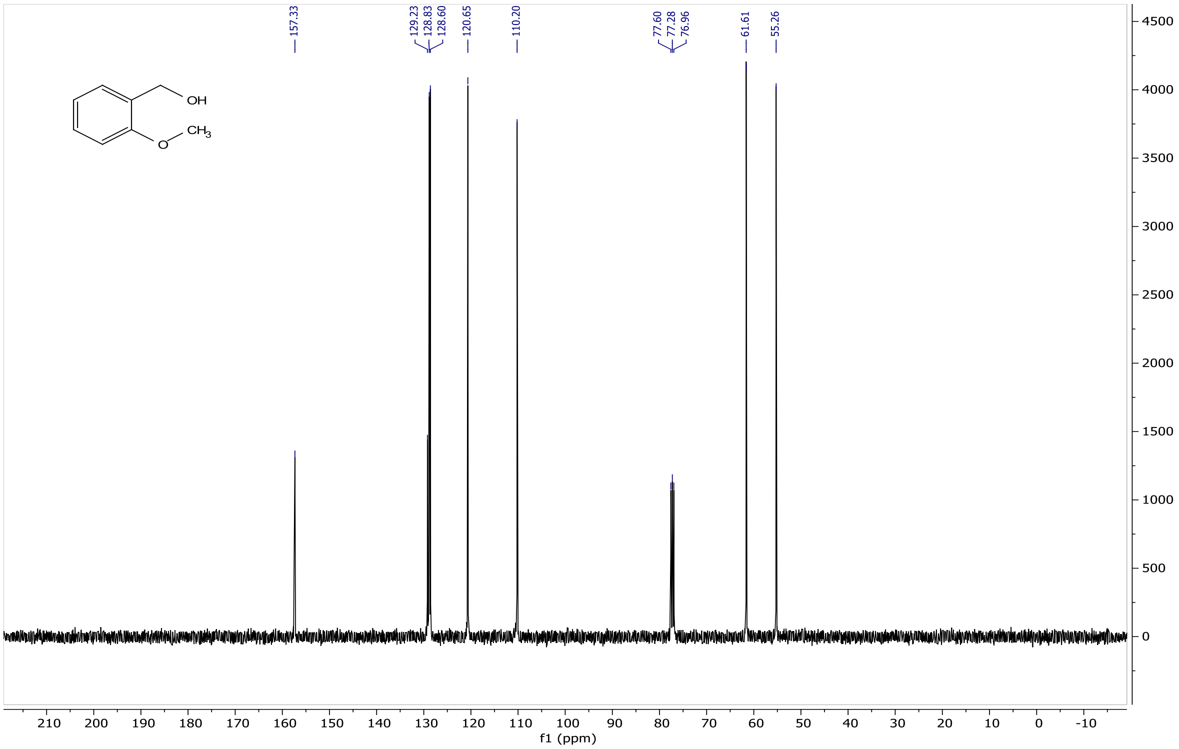

Figure S63. ^{13}C NMR spectrum of **4k** in $CDCl_3$ (101 MHz)

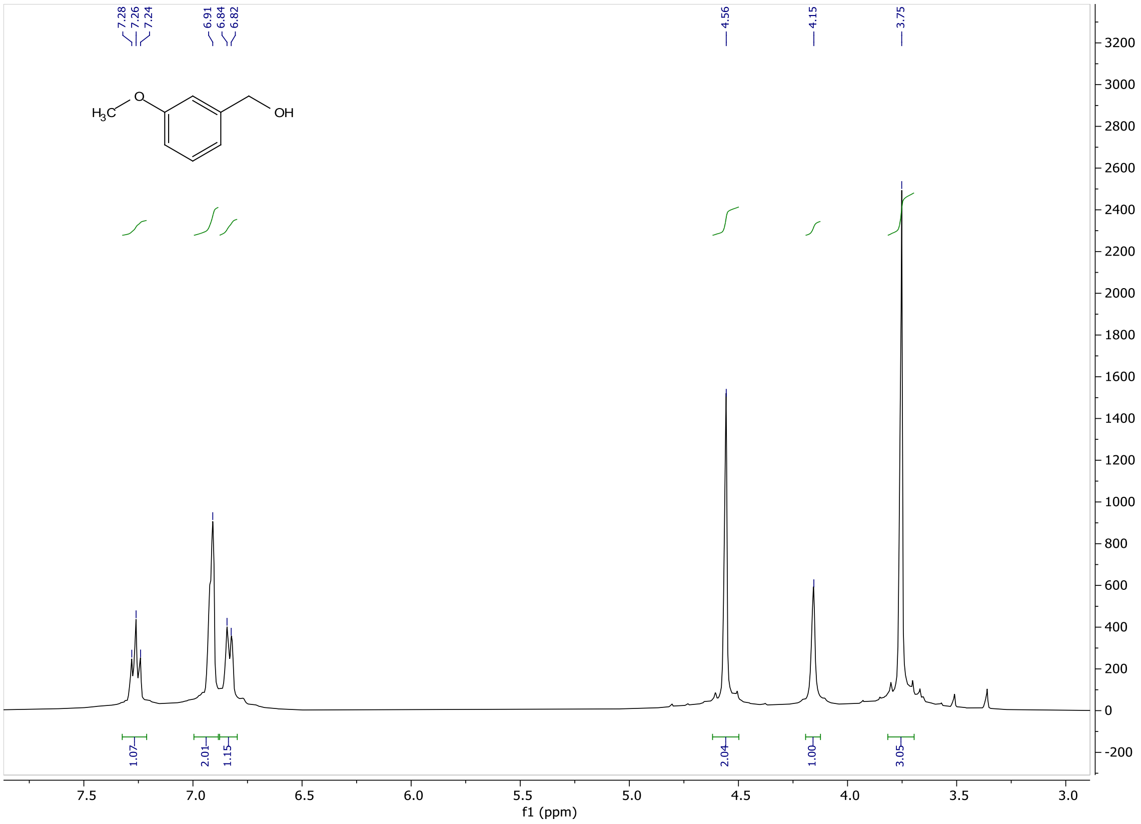

Figure S64. ^{1}H NMR spectrum of **4l** in $CDCl_3$ (400 MHz)

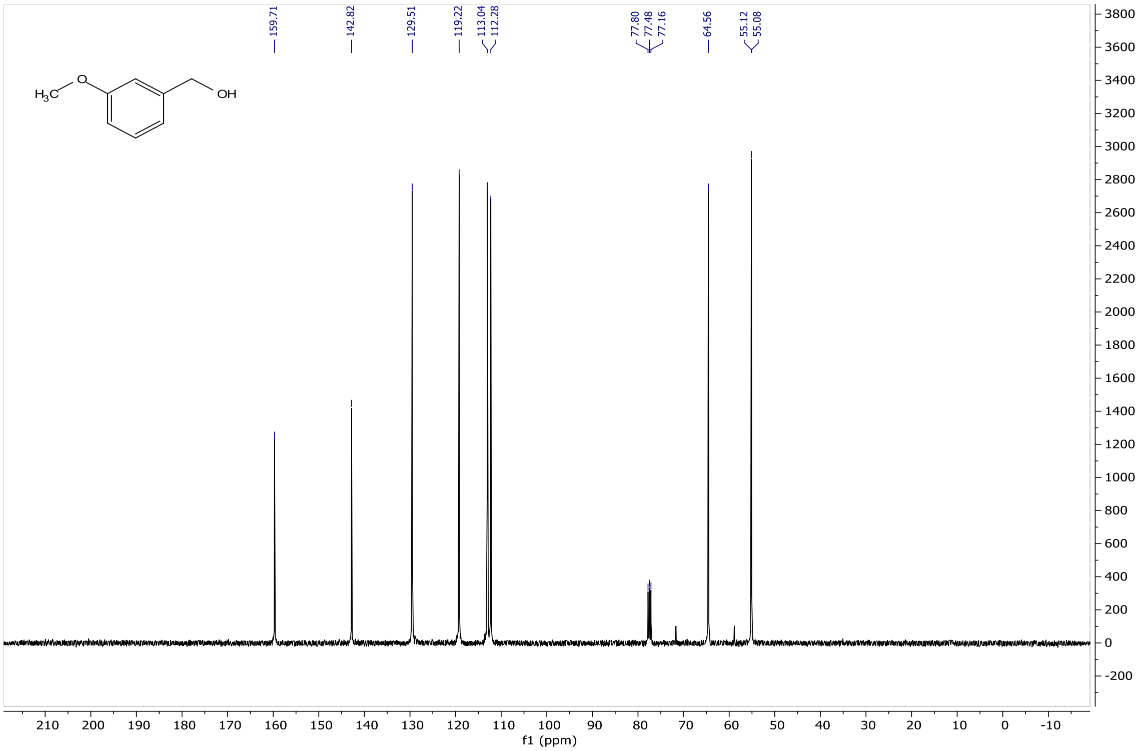

Figure S65. ^{13}C NMR spectrum of **4l** in $CDCl_3$ (101 MHz)

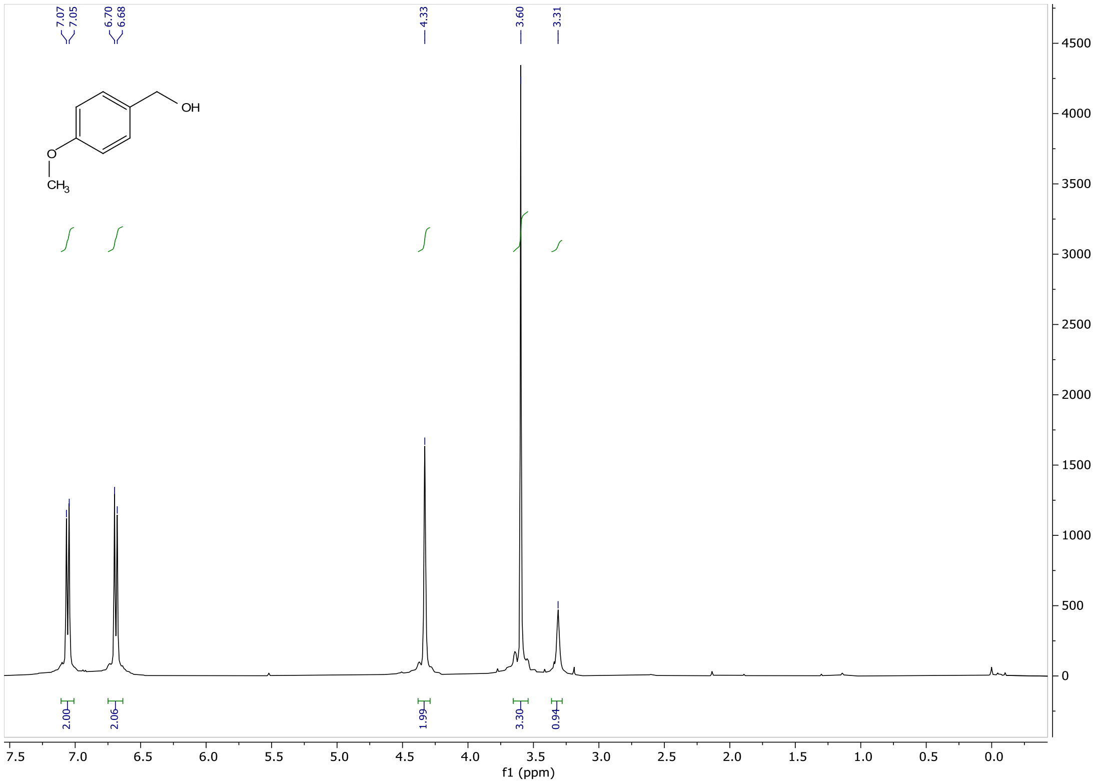

Figure S66. ^{1}H NMR spectrum of **4m** in $CDCl_3$ (400 MHz)

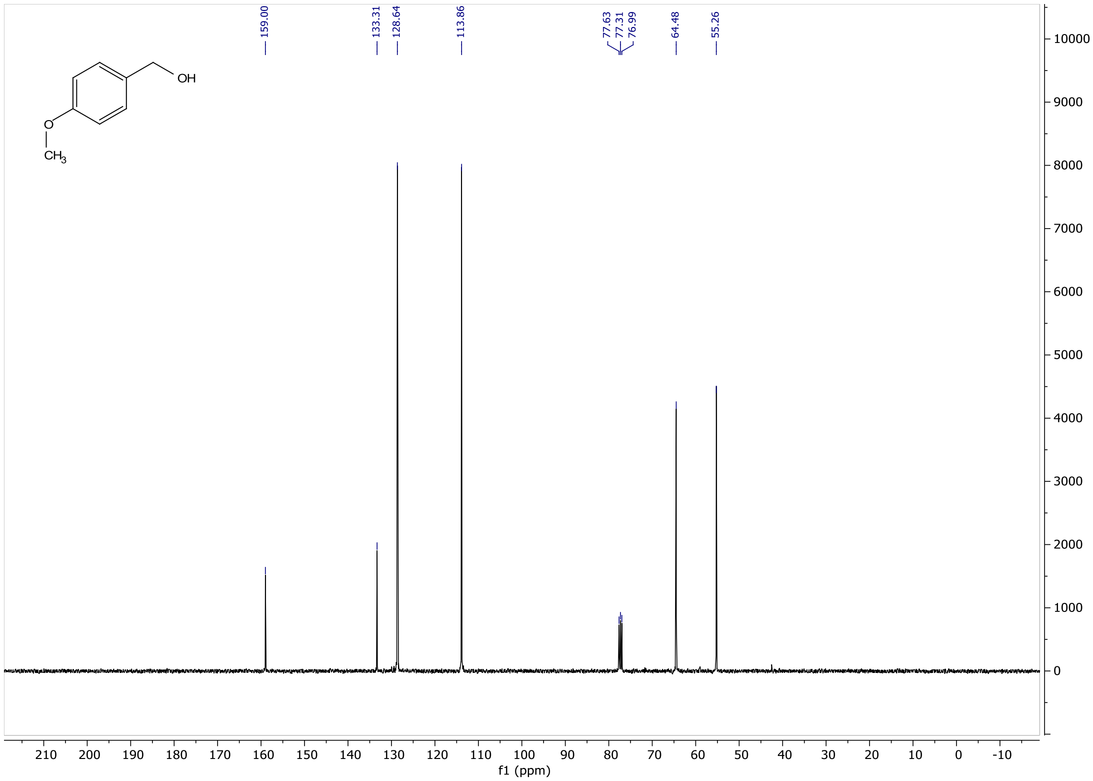

Figure S67. ^{13}C NMR spectrum of **4m** in $CDCl_3$ (101 MHz)

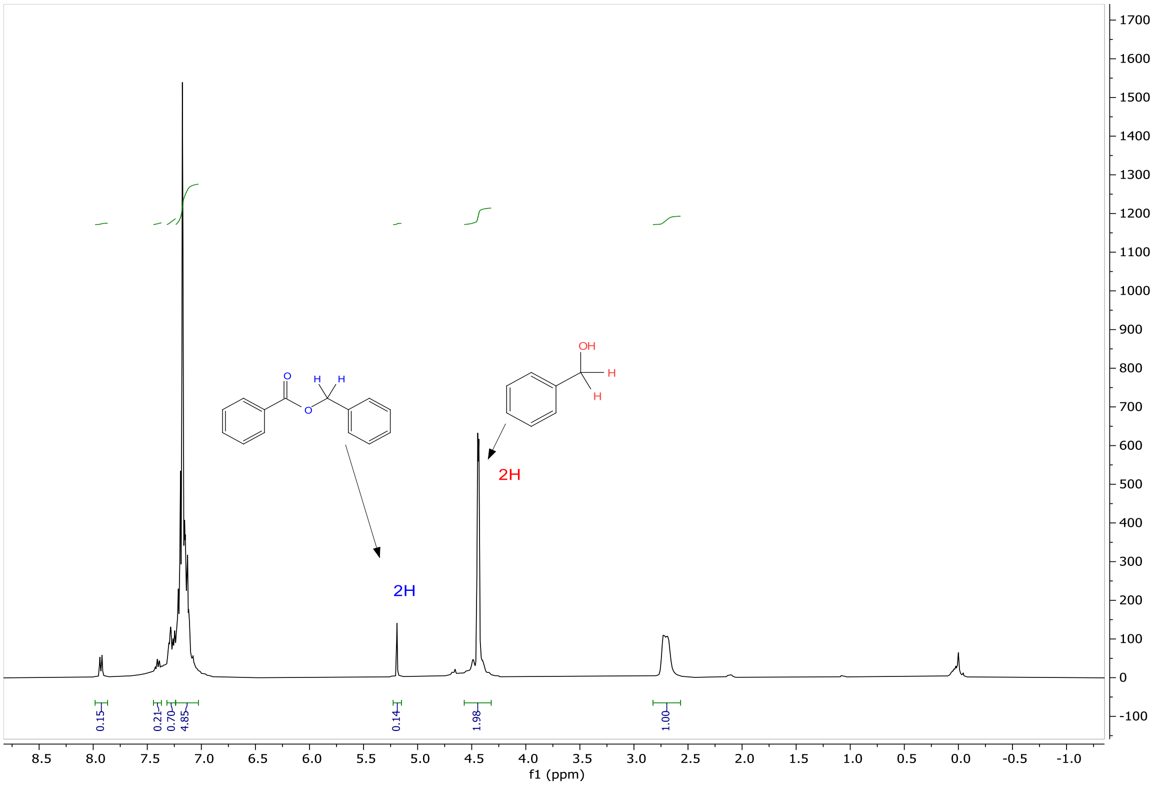

Figure S68. ^1H NMR spectrum of **4n** in $CDCl_3$ (400 MHz)

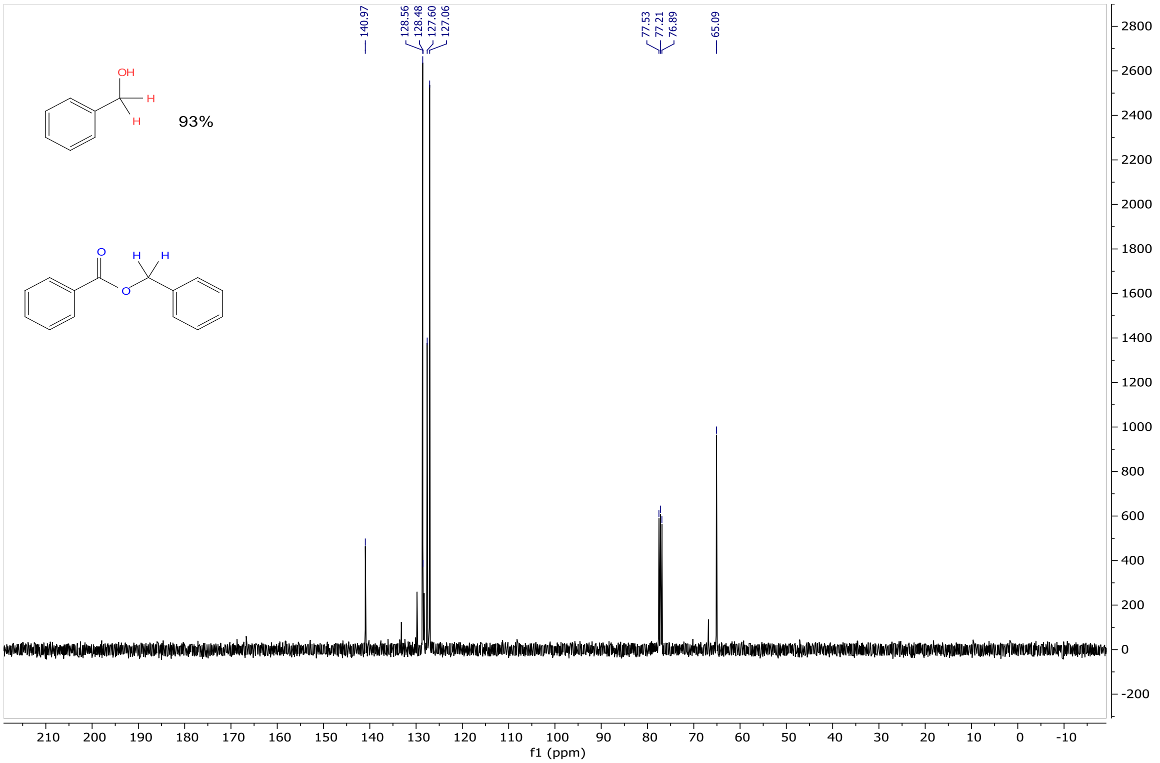

Figure S69. ^{13}C NMR spectrum of **4n** in $CDCl_3$ (101 MHz)

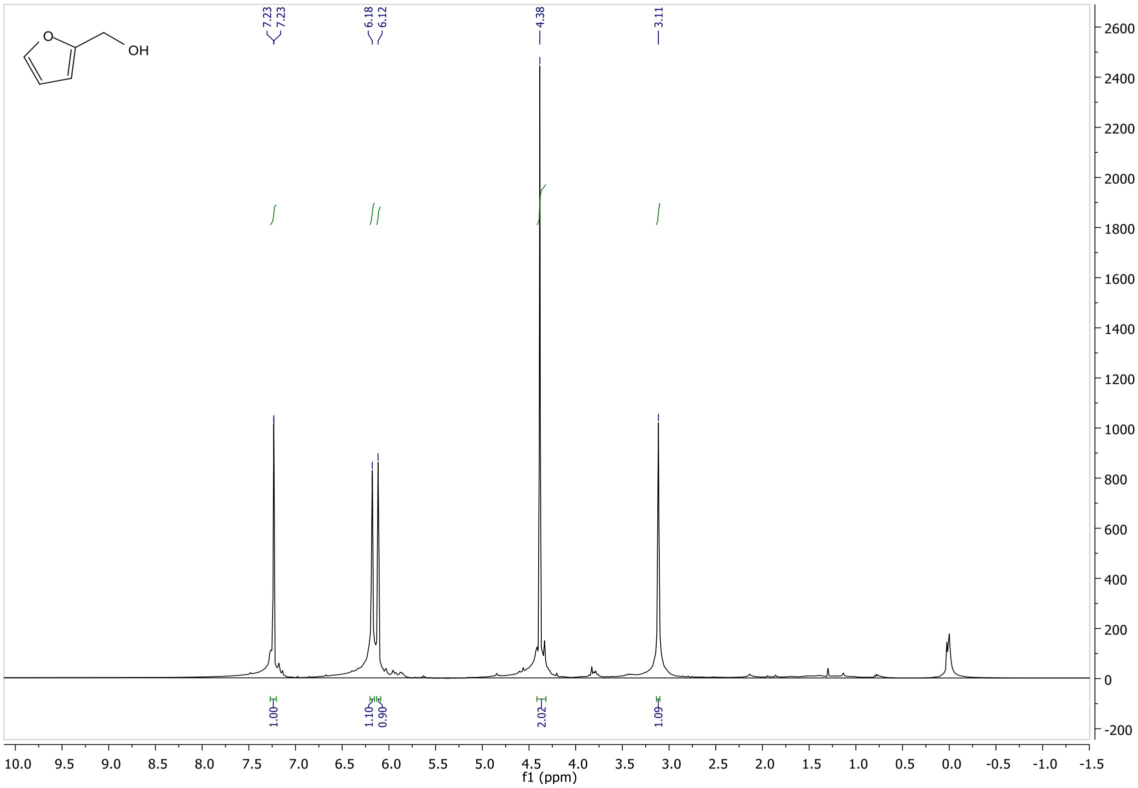

Figure S70. ^{1}H NMR spectrum of **4o** in $CDCl_3$ (400 MHz)

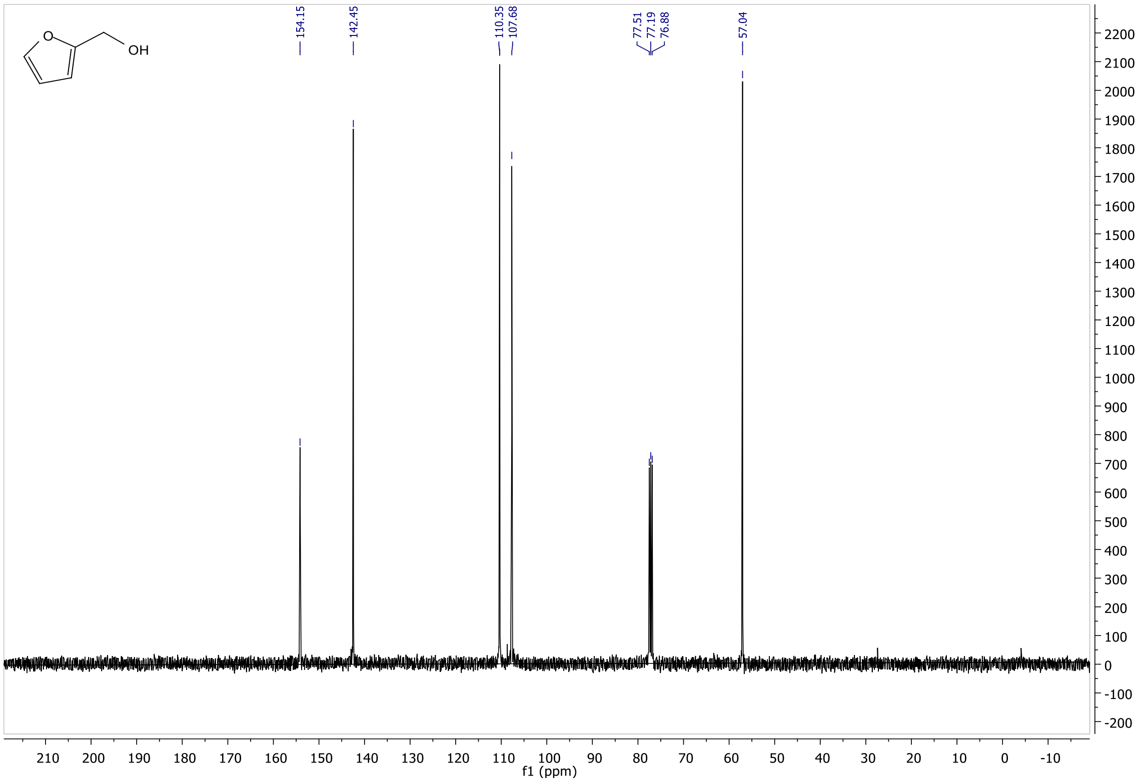

Figure S71. ^{13}C NMR spectrum of **4o** in $CDCl_3$ (101 MHz)

8. NMR Analysis for Control Experiments

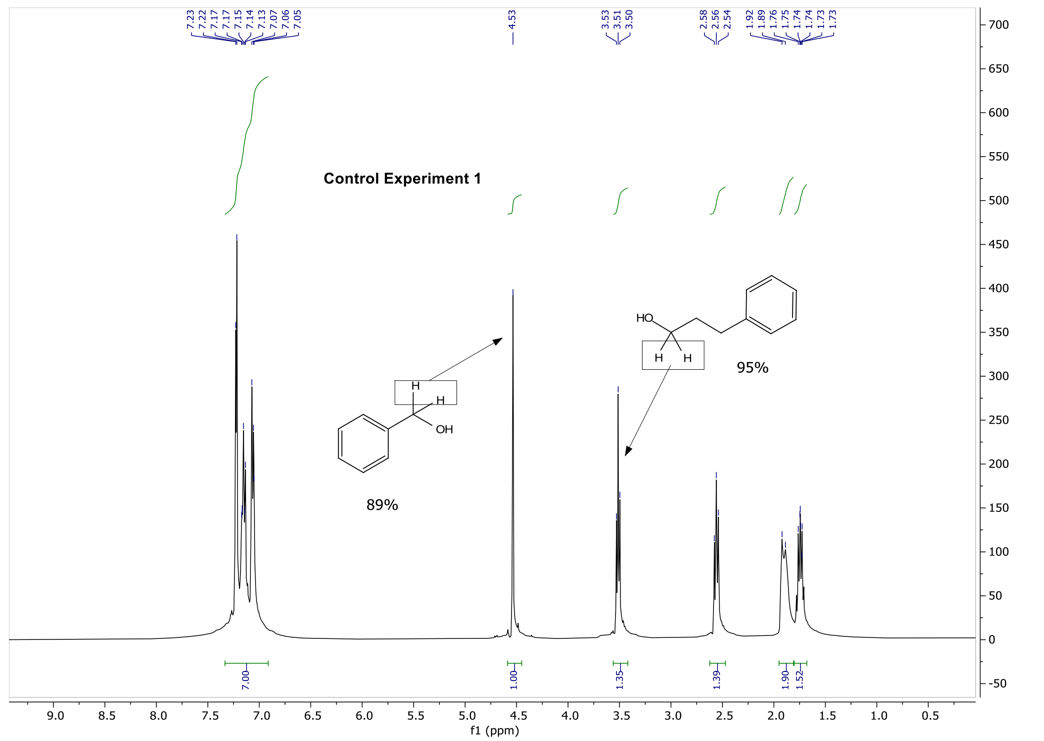

Figure S72. ^{1}H NMR spectrum of **control experiment 1** in $CDCl_3$ (400 MHz)

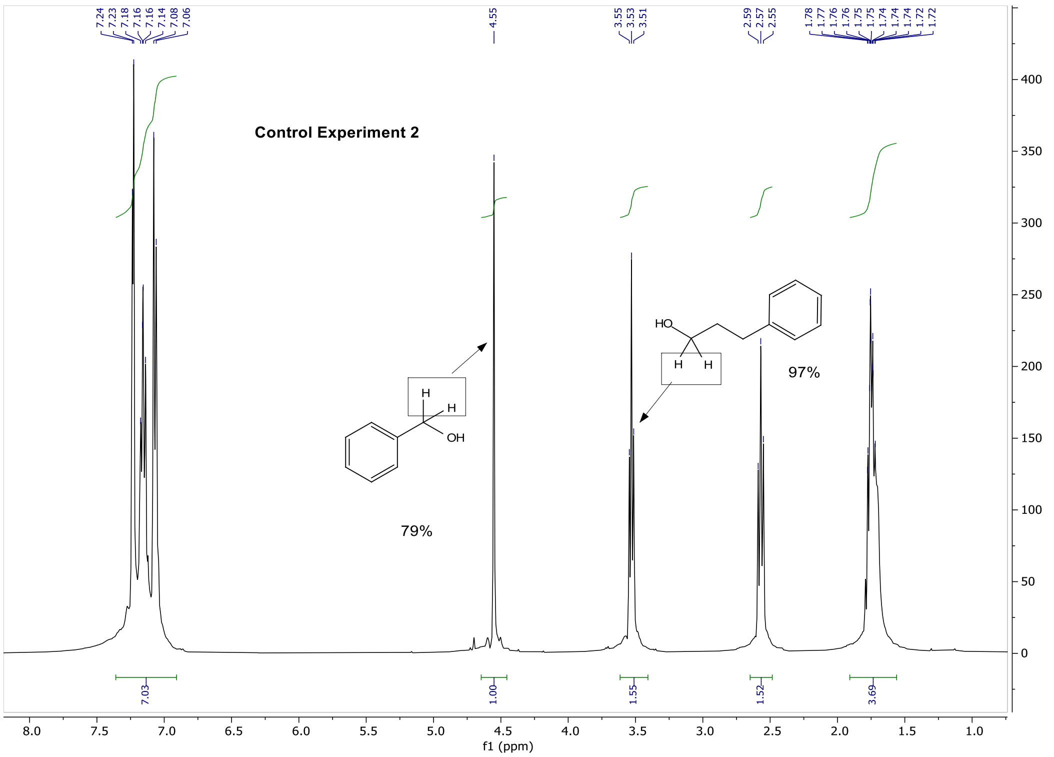

Figure S73. ^{1}H NMR spectrum of **control experiment 2** in $CDCl_3$ (400 MHz)

9. GC-MS data of compounds

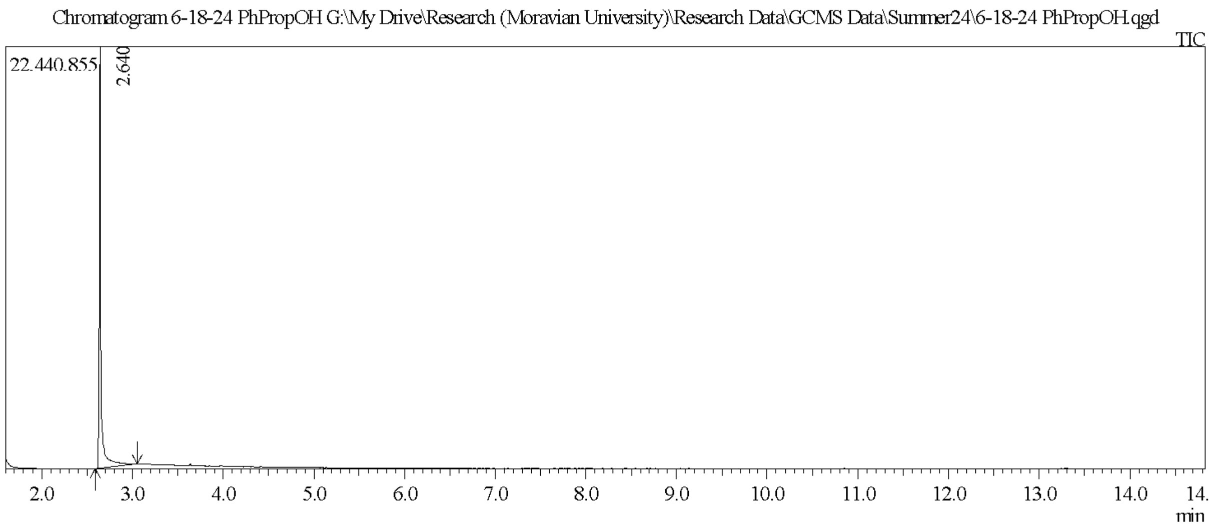

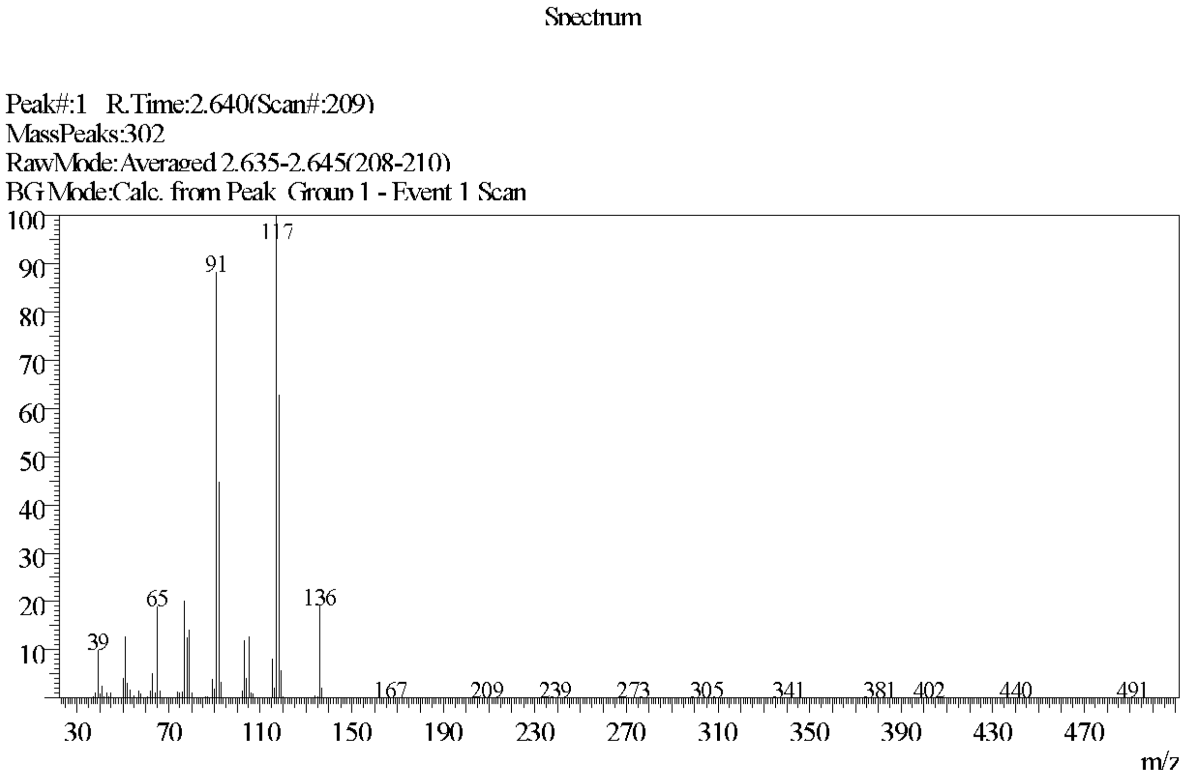

Figure S74. GC-MS of **2a**

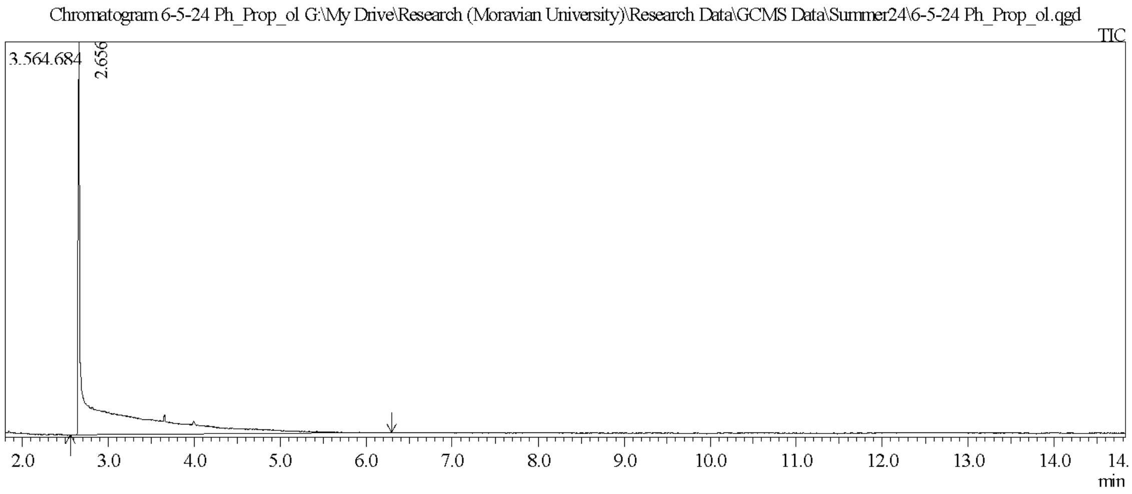

Spectrum

Peak#:1 R.Time:2.656(Scan#:172)
MassPeaks:303
RawMode:Averaged 2.650-2.660(171-173)
BG Mode:Calc. from Peak Group 1 - Event 1 Scan

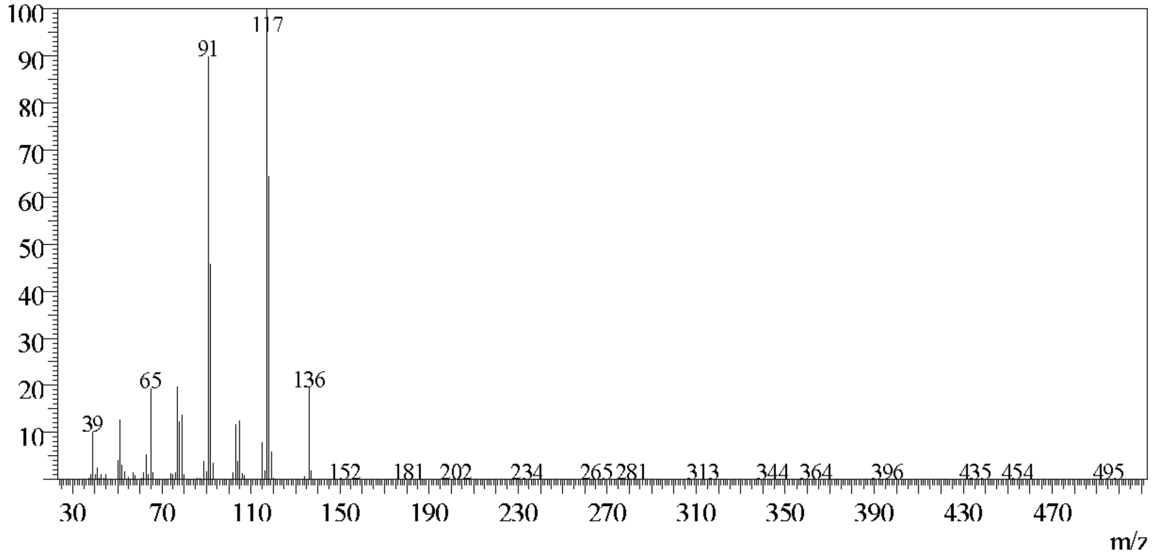

Figure S75. GC-MS of **2b**

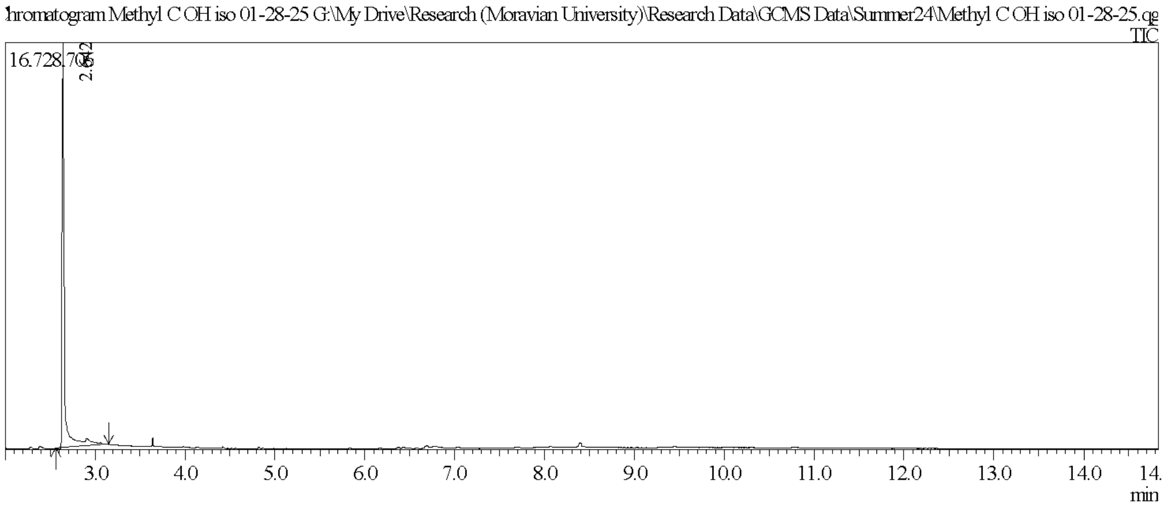

Spectrum

Peak#:1 R.Time:2.642(Scan#:129)
MassPeaks:334
RawMode:Averaged 2.635-2.645(128-130)
BG Mode:Calc. from Peak Group 1 - Event 1 Scan

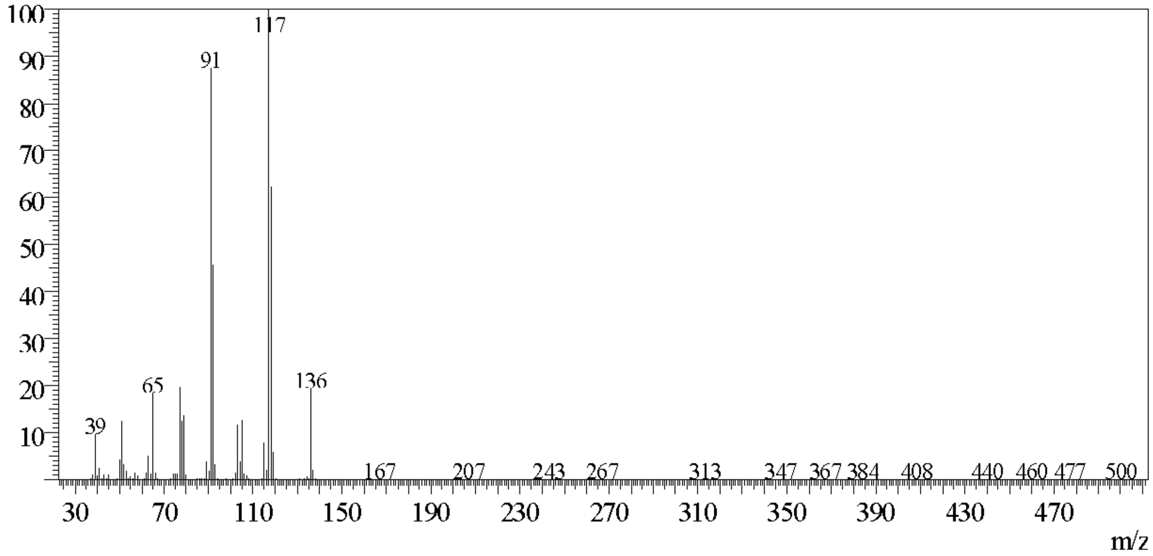

Figure S76. GC-MS of **2c**

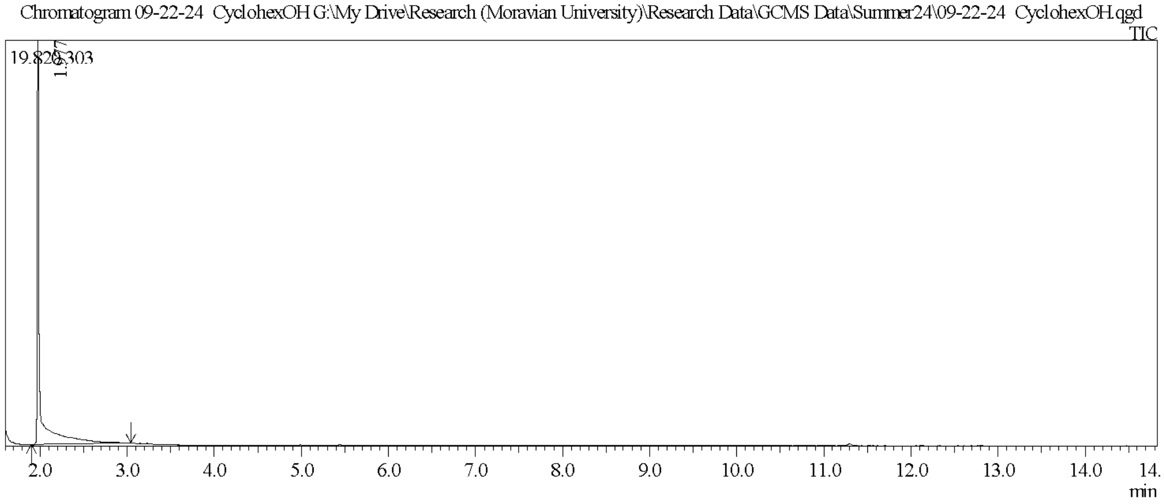

Spectrum

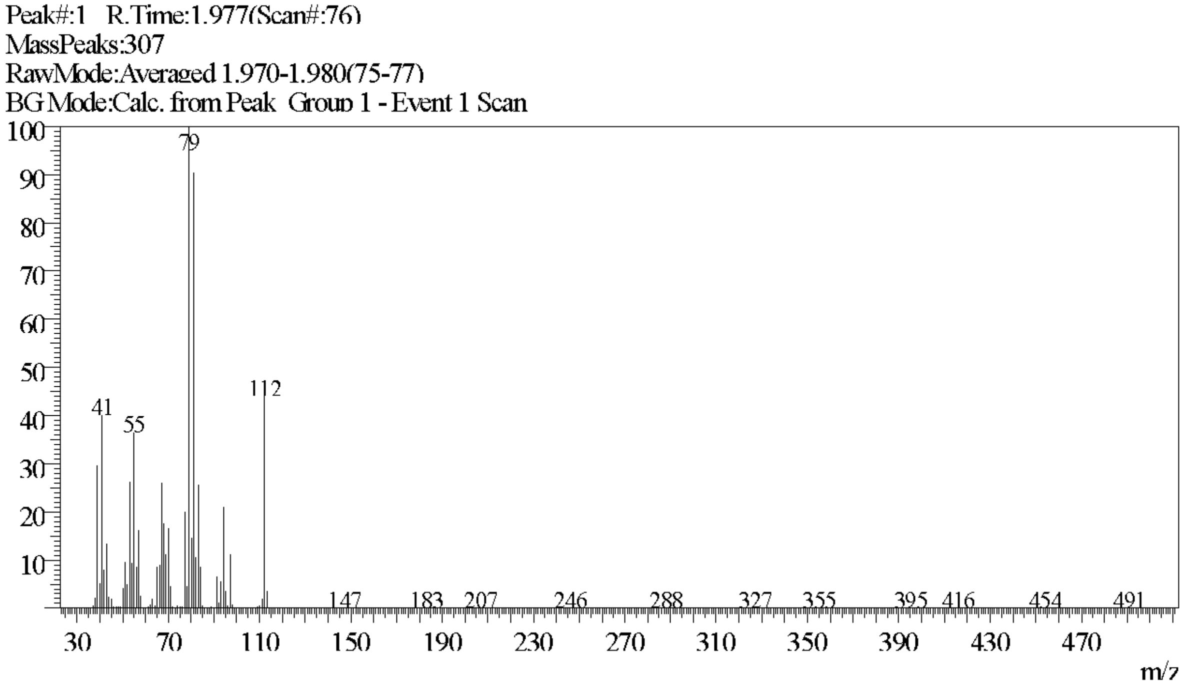

Figure S77. GC-MS of **2d**

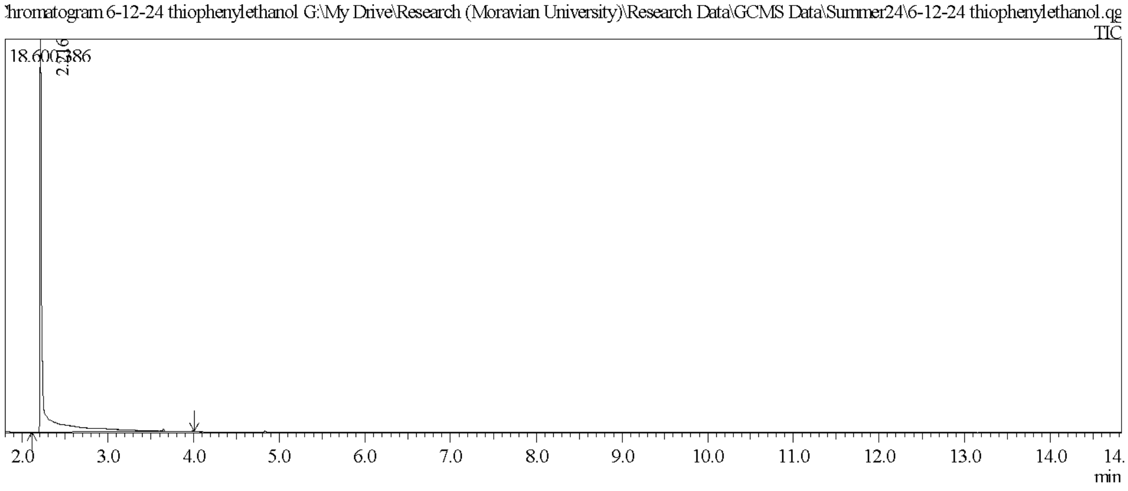

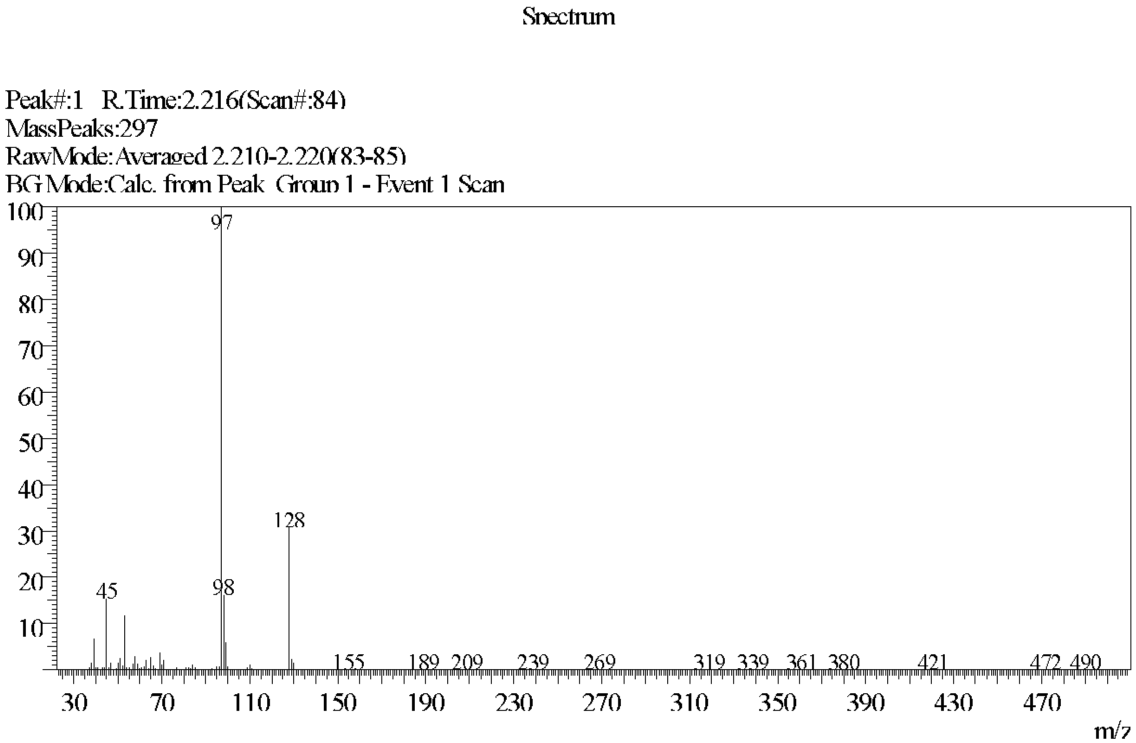

Figure S78. GC-MS of **2e**

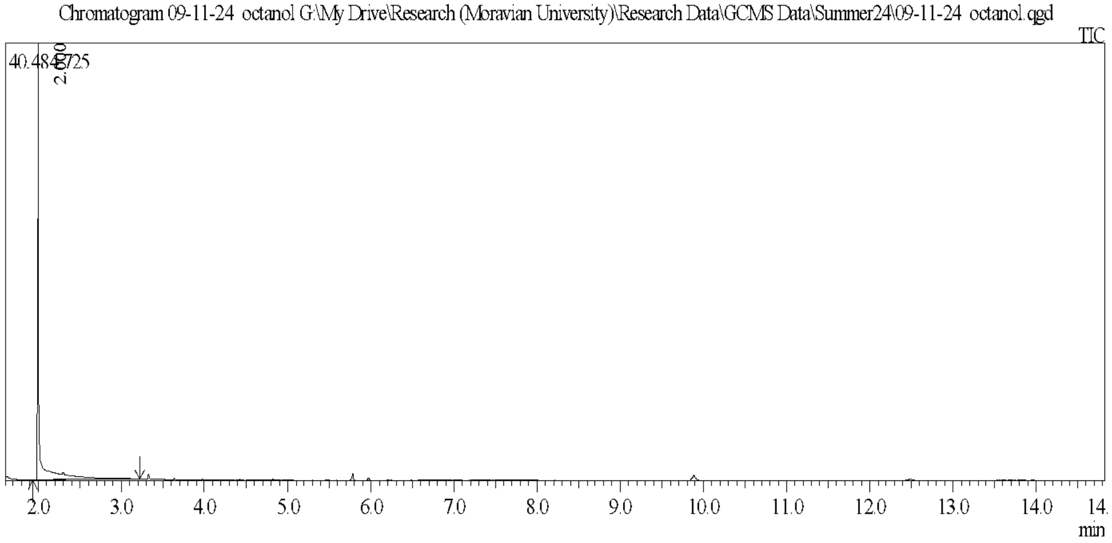

Spectrum

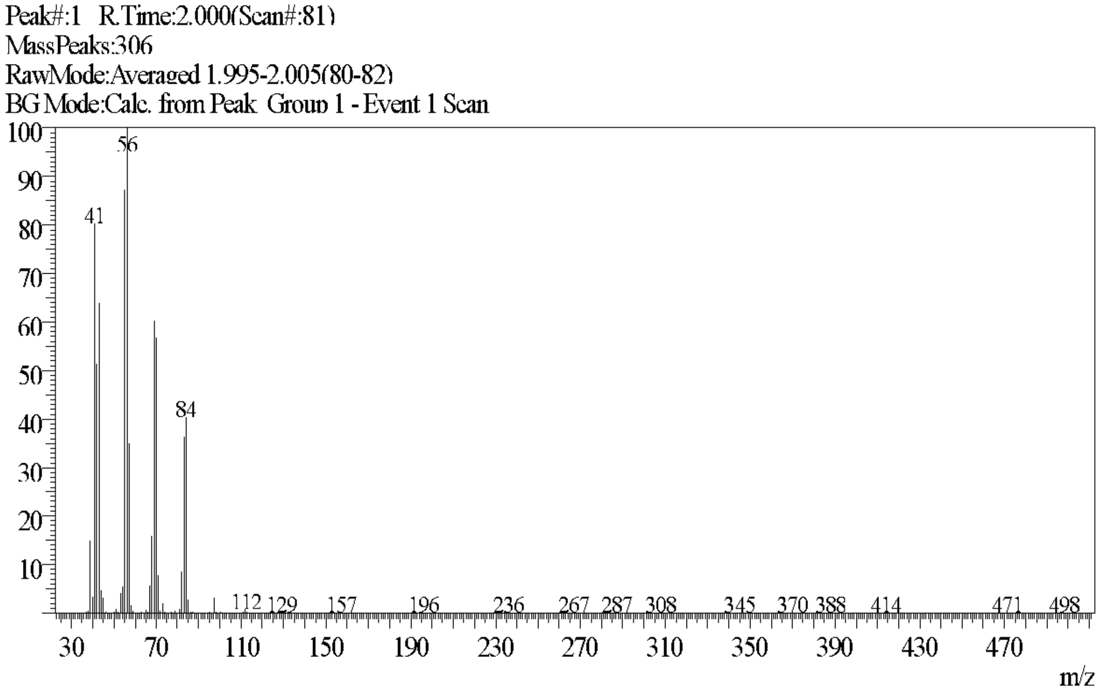

Figure S79. GC-MS of **2f**

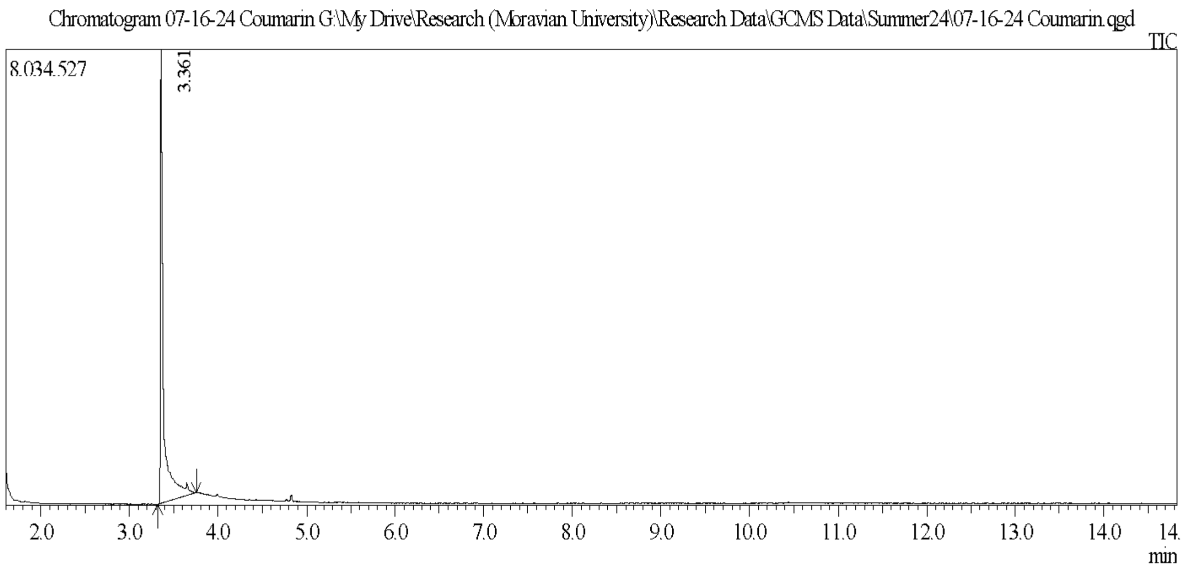

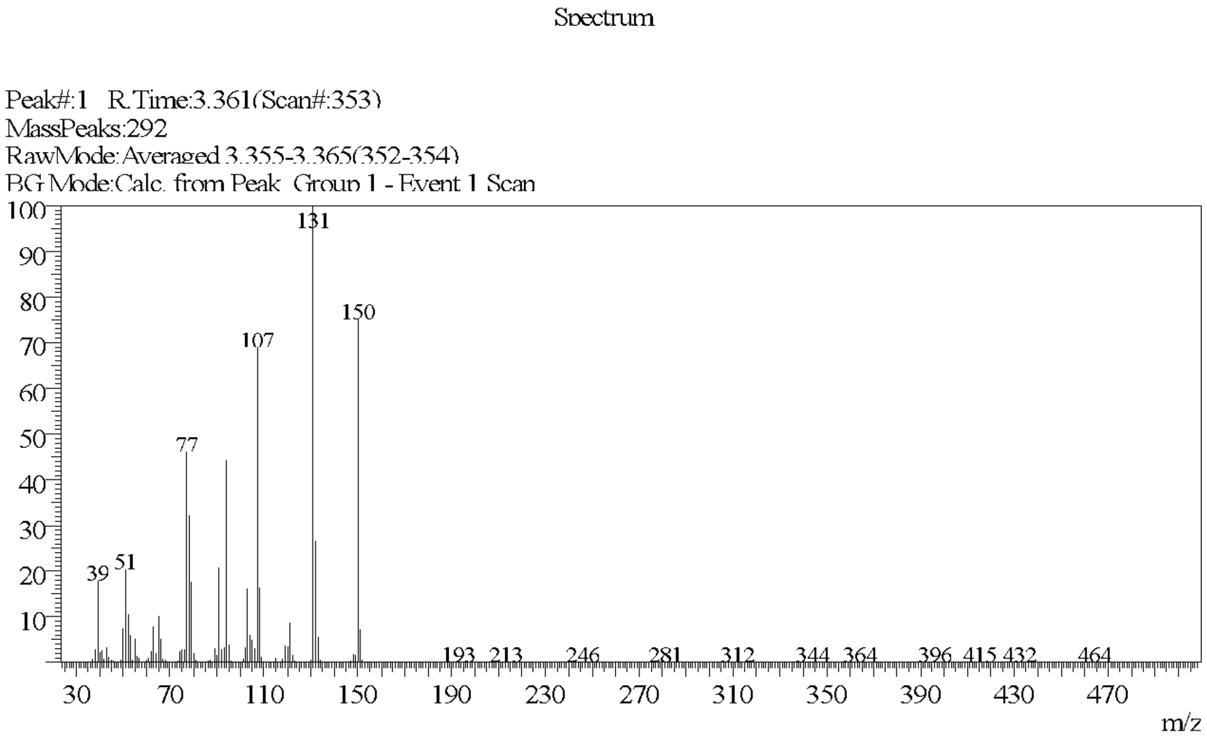

Figure S80. GC-MS of **2g**

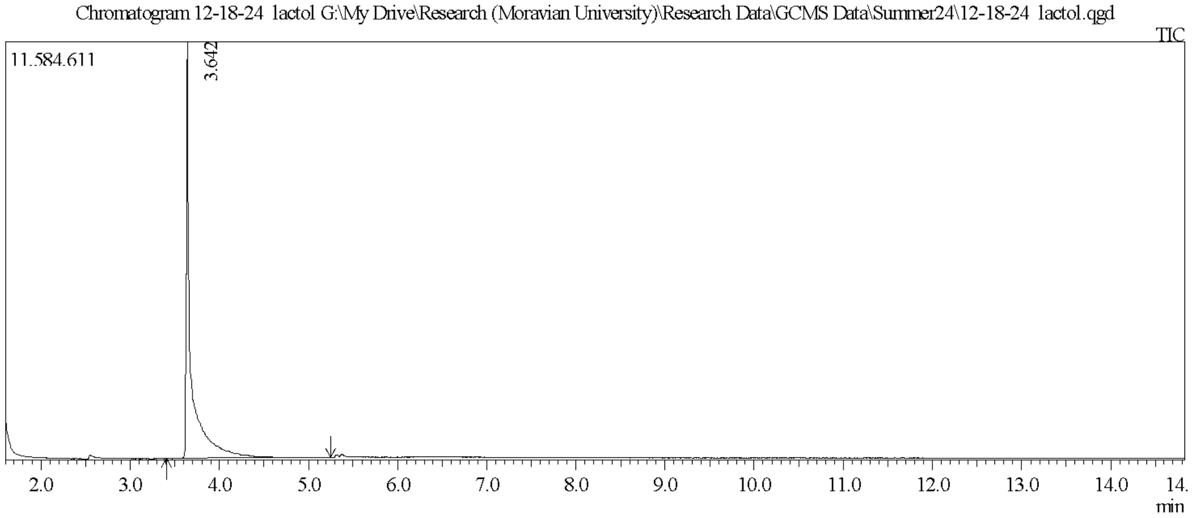

Spectrum

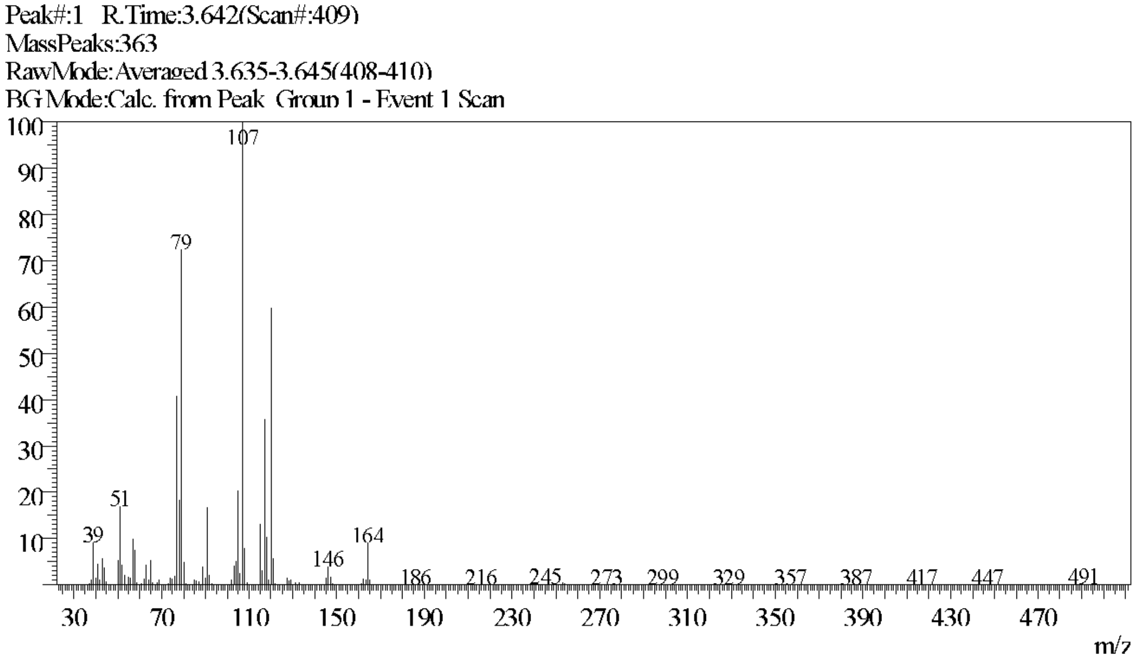

Figure S81. GC-MS of **2h**

Chromatogram 01-06-25 Menthol_2nd G:\My Drive\Research (Moravian University)\Research Data\GCMS Data\Summer24\01-06-25 Menthol_2nd.qgd

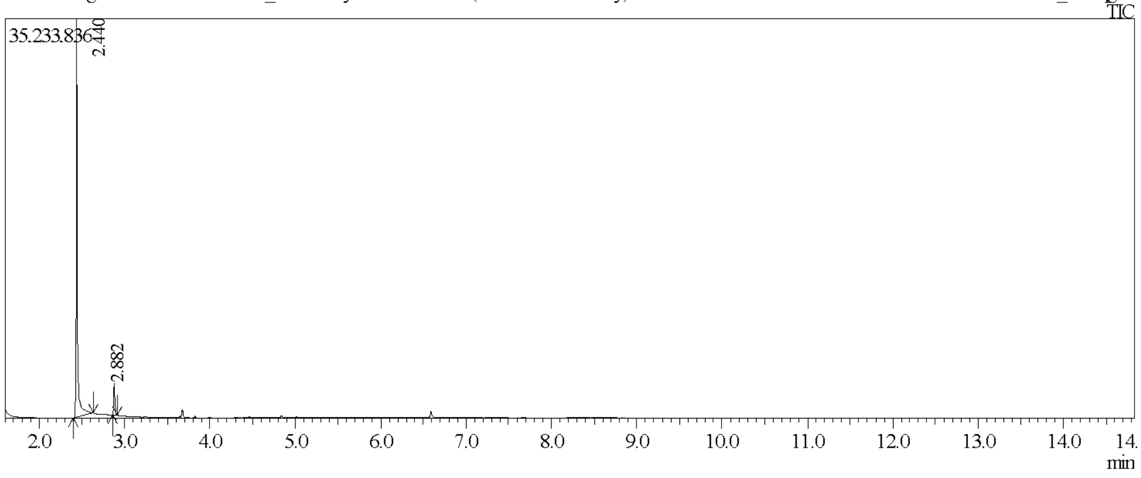

Spectrum

Peak#:1 R.Time:2.440(Scan#:169)
MassPeaks:315
RawMode:Averaged 2.435-2.445(168-170)
BG Mode:Calc. from Peak Group 1 - Event 1 Scan

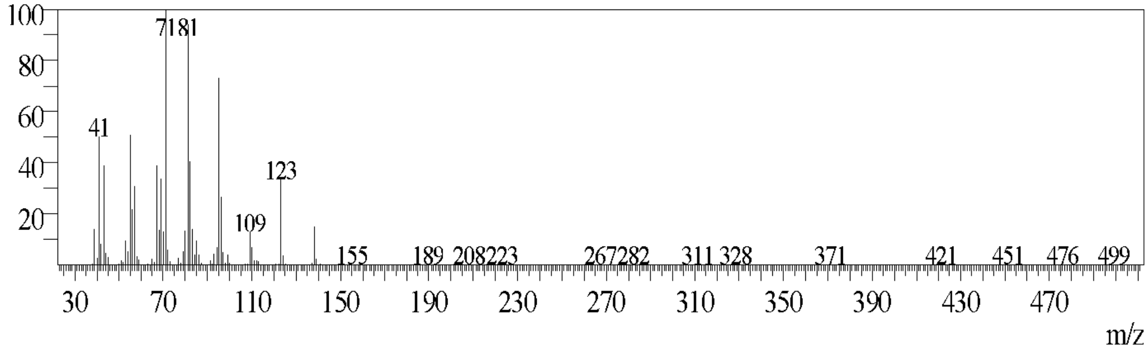

Spectrum

Peak#:2 R.Time:2.882(Scan#:257)
MassPeaks:295
RawMode:Averaged 2.875-2.885(256-258)
BG Mode:Calc. from Peak Group 1 - Event 1 Scan

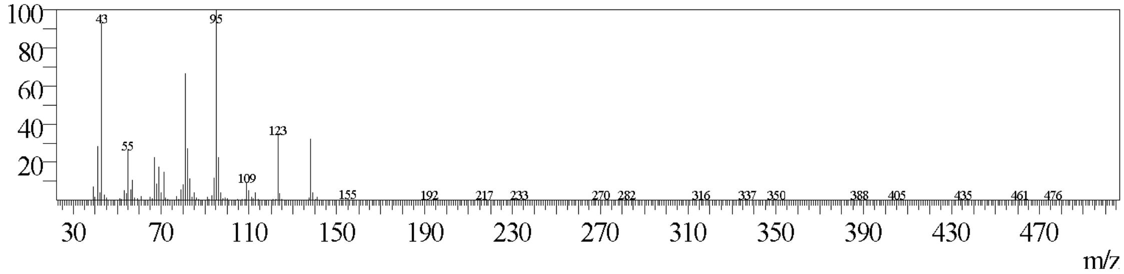

Figure S82. GC-MS of **2j**

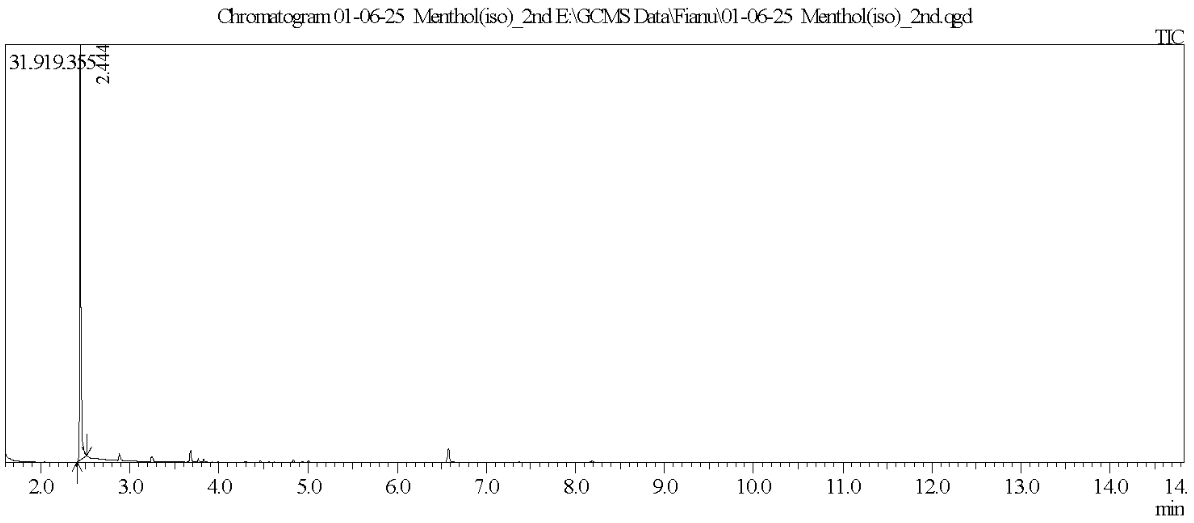

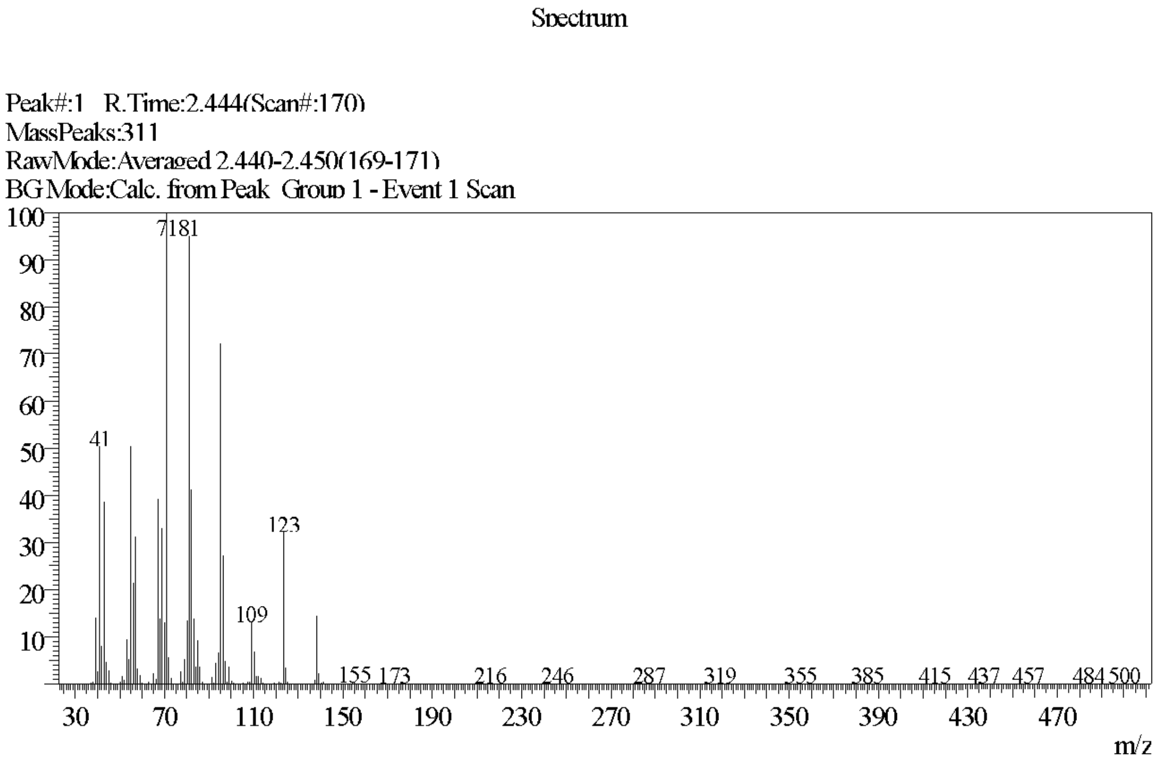

Figure S83. GC-MS of **2j** (isopropanol added)

Chromatogram 6-24-24 BenzOH G:\My Drive\Research (Moravian University)\Research Data\GCMS Data\Summer24\6-24-24 BenzOH.qgd

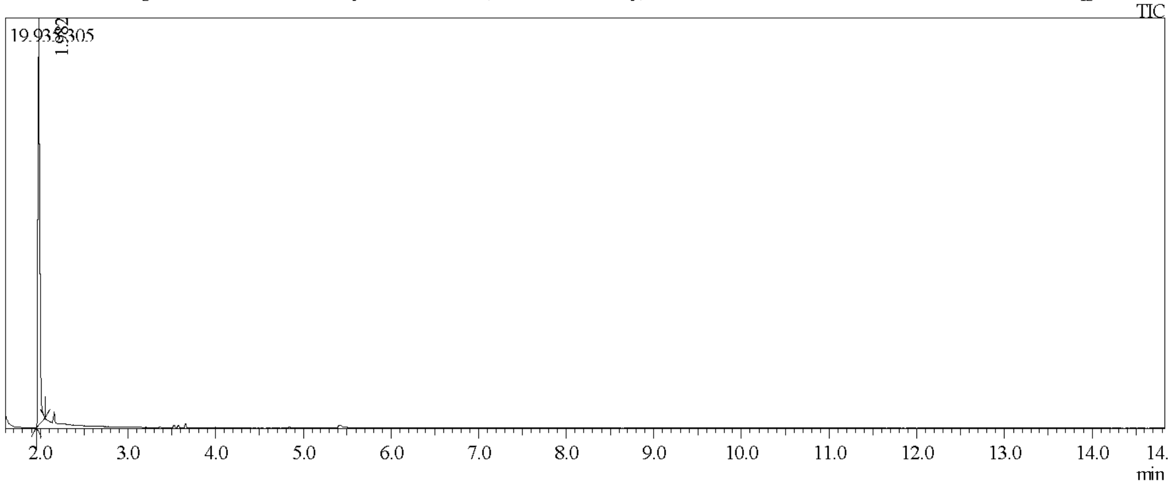

Spectrum

Peak#:1 R.Time:1.982(Scan#:77)
MassPeaks:287
RawMode:Averaged 1.975-1.985(76-78)
BG Mode:Calc. from Peak Group 1 - Event 1 Scan

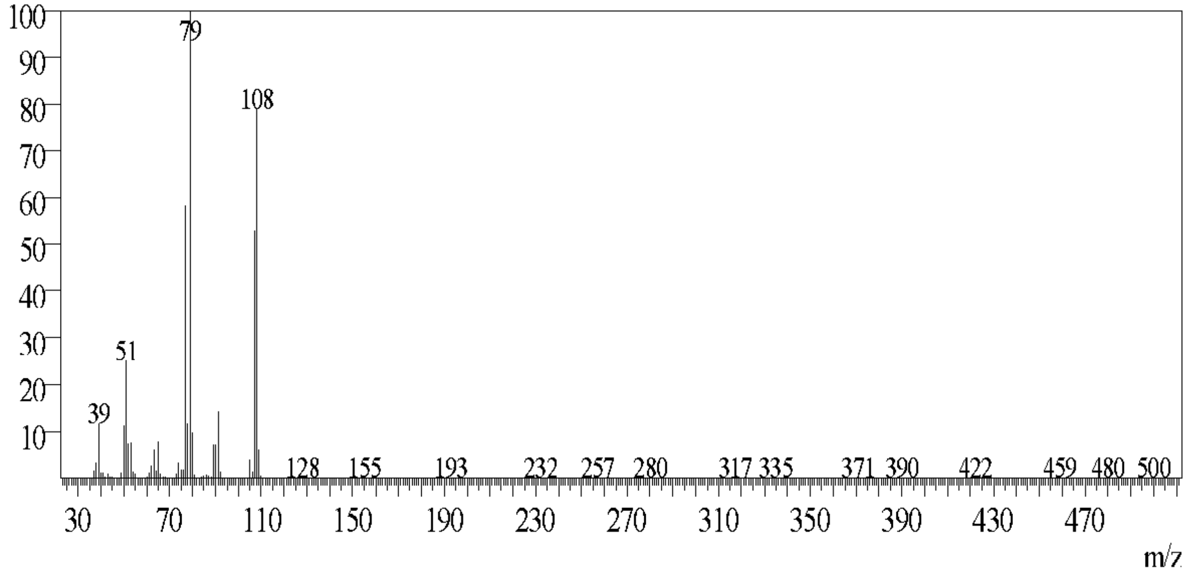

Figure S84. GC-MS of **4a**

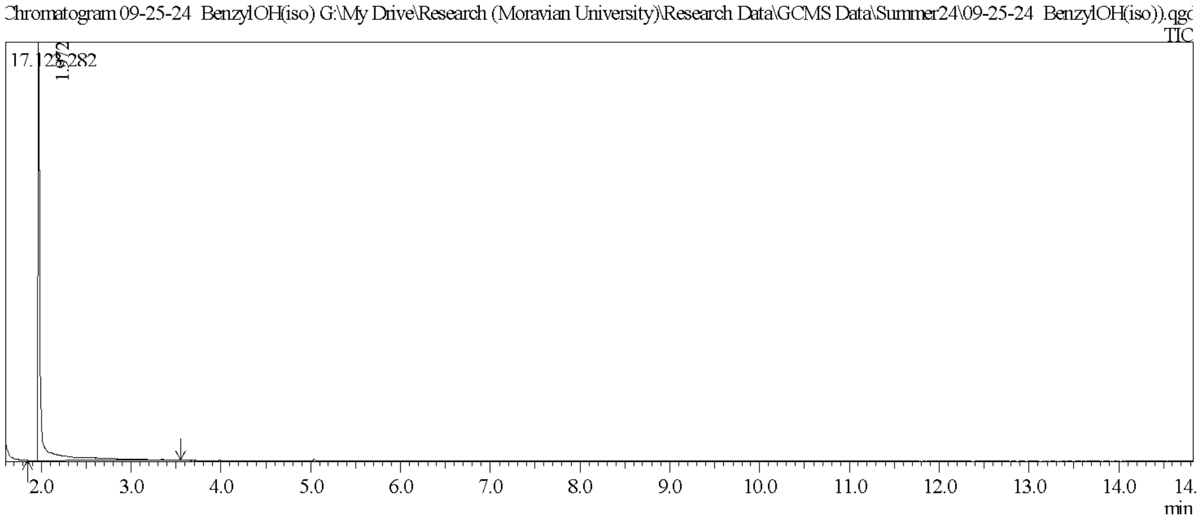

Spectrum

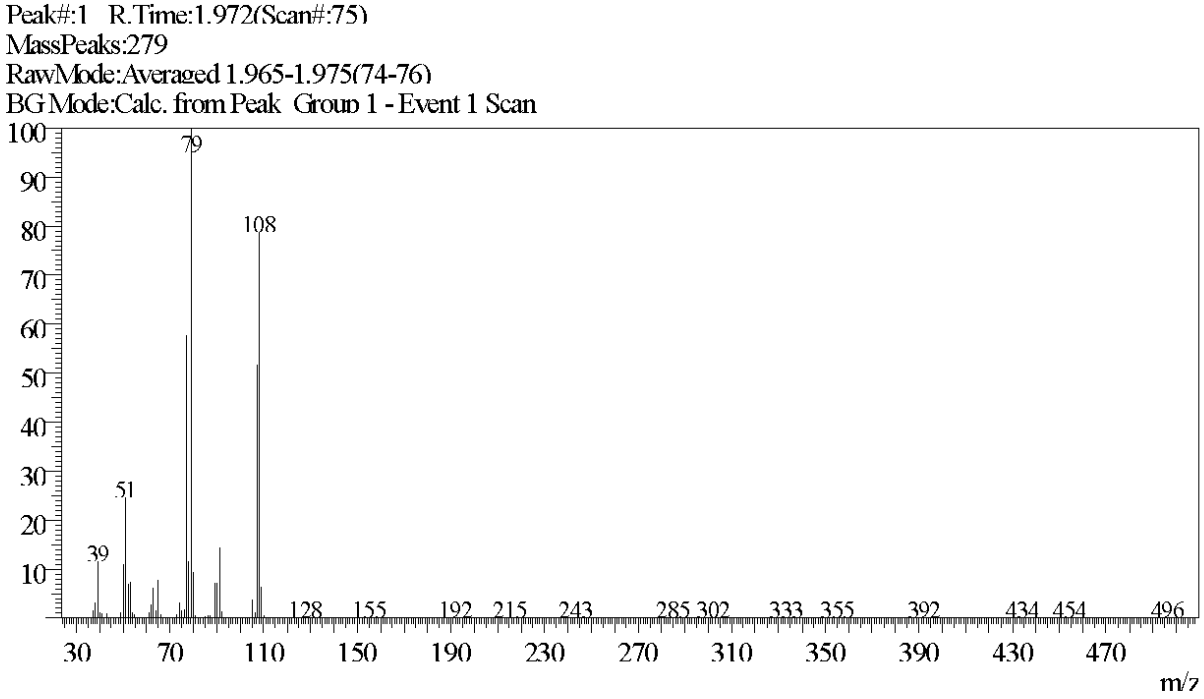

Figure S85. GC-MS of **4b**

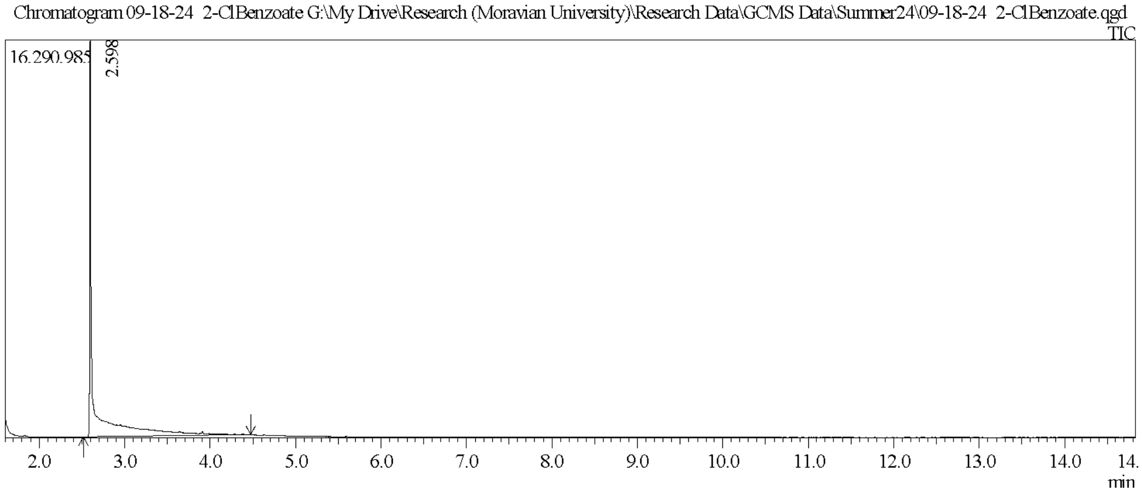

Spectrum

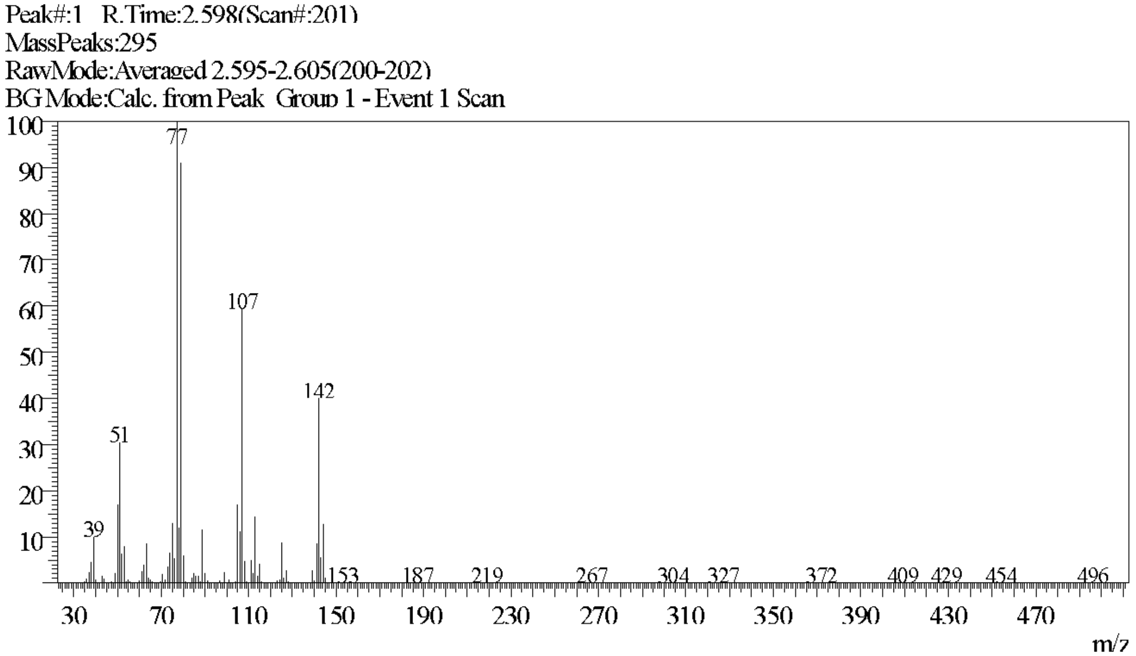

Figure S86. GC-MS of **4c**

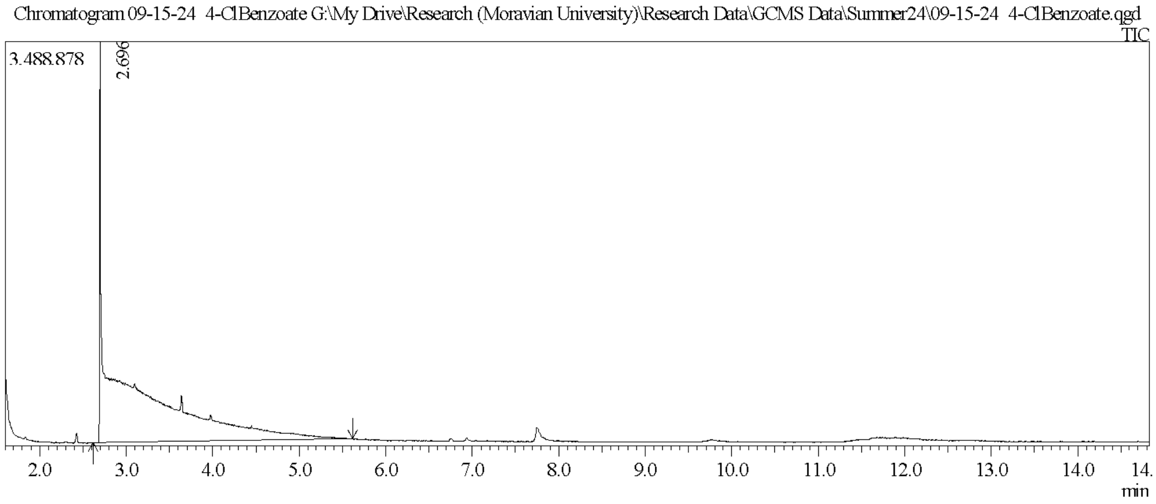

Spectrum

Peak#:1 R.Time:2.696(Scan#:220)
MassPeaks:315
RawMode:Averaged 2.690-2.700(219-221)
BG Mode:Calc. from Peak Group 1 - Event 1 Scan

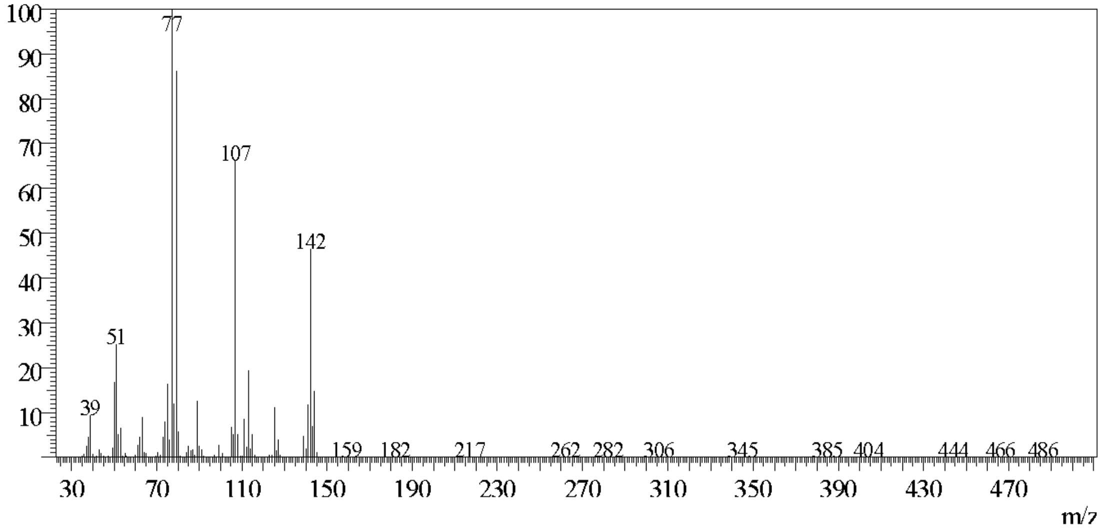

Figure S87. GC-MS of **4d**

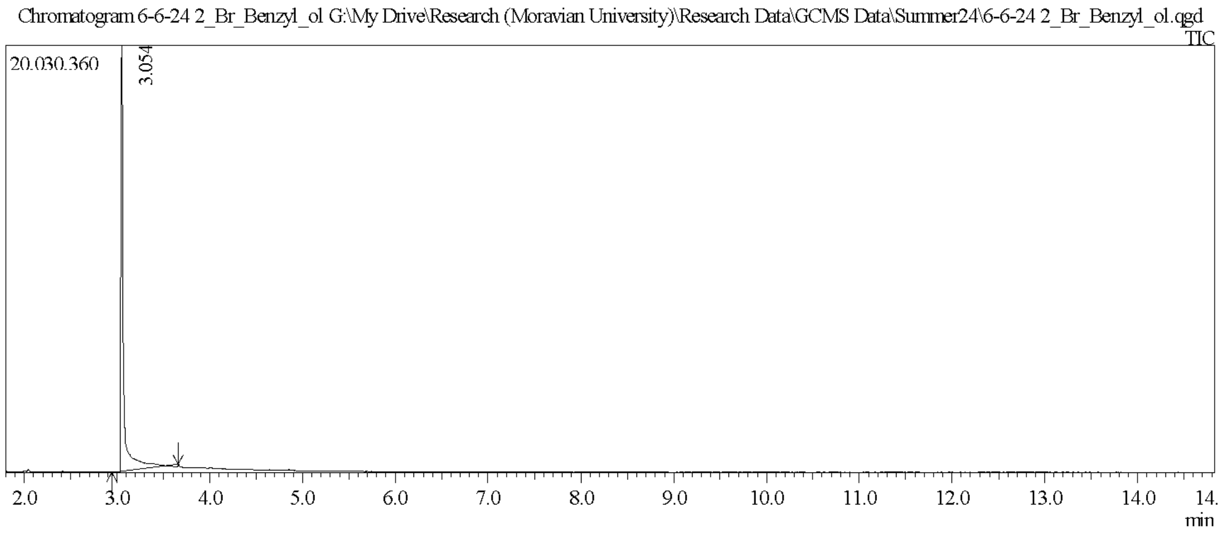

Spectrum

Peak#:1 R.Time:3.054(Scan#:252)
MassPeaks:290
RawMode:Averaged 3.050-3.060(251-253)
BG Mode:Calc. from Peak Group 1 - Event 1 Scan

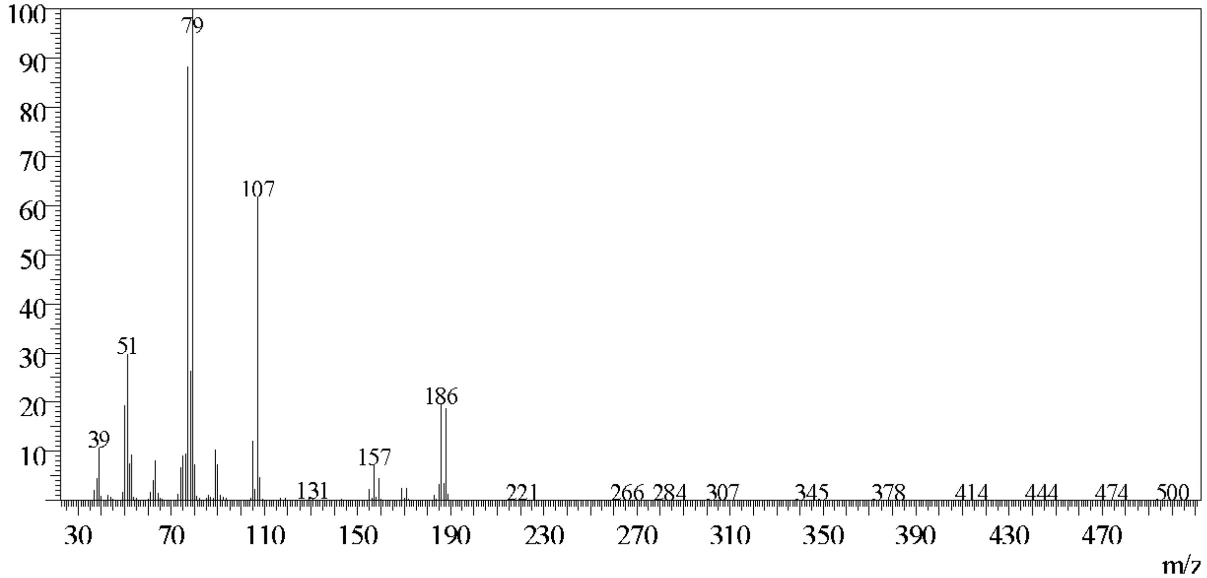

Figure S88. GC-MS of **4e**

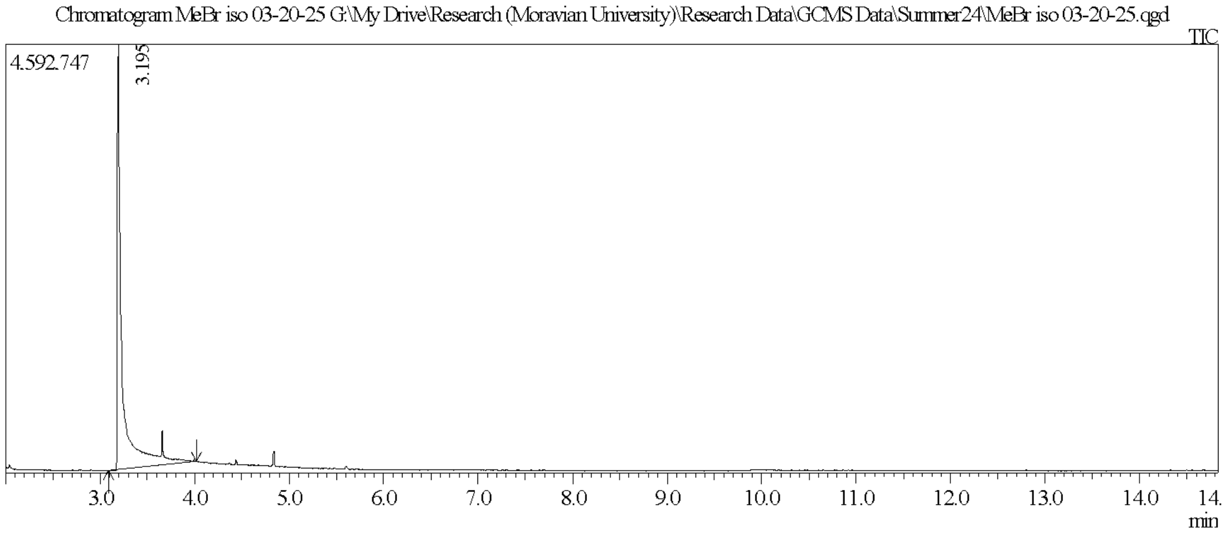

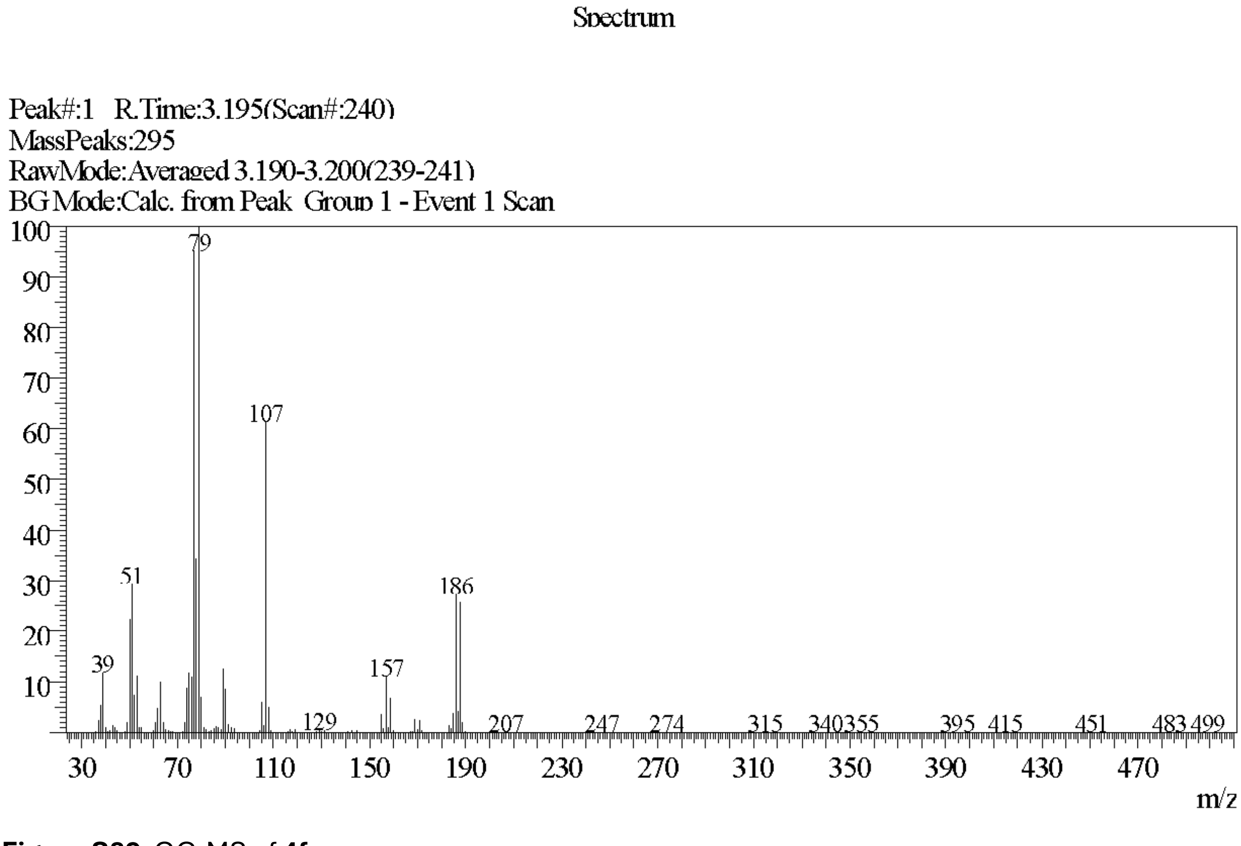

Figure S89. GC-MS of **4f**

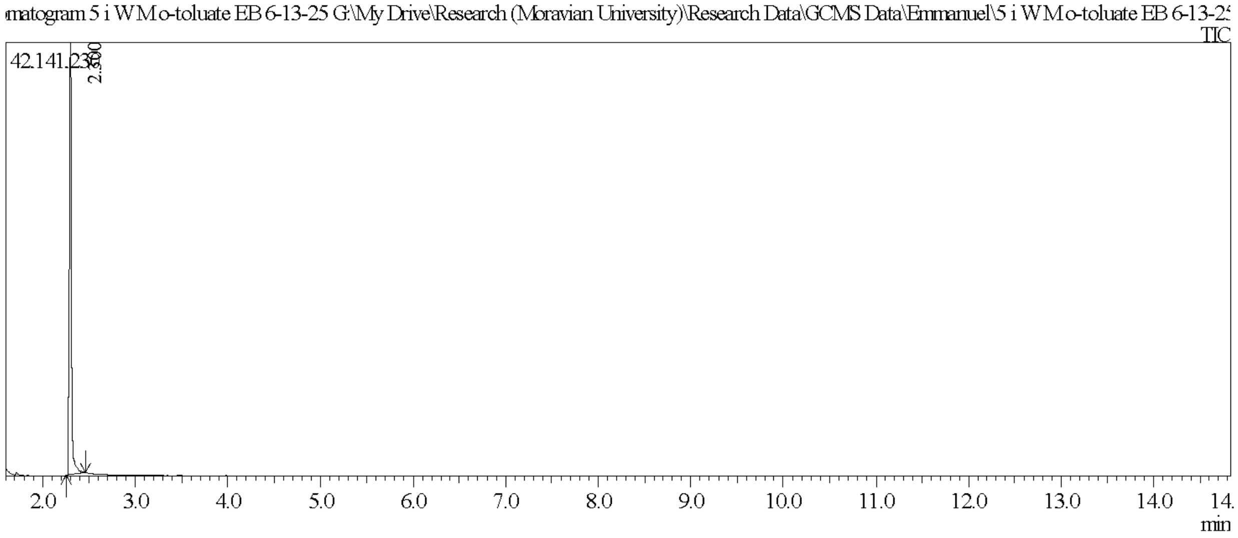

Spectrum

Peak#:1 R.Time:2.300(Scan#:141)
MassPeaks:321
RawMode:Averaged 2.295-2.305(140-142)
BG Mode:Calc. from Peak Group 1 - Event 1 Scan

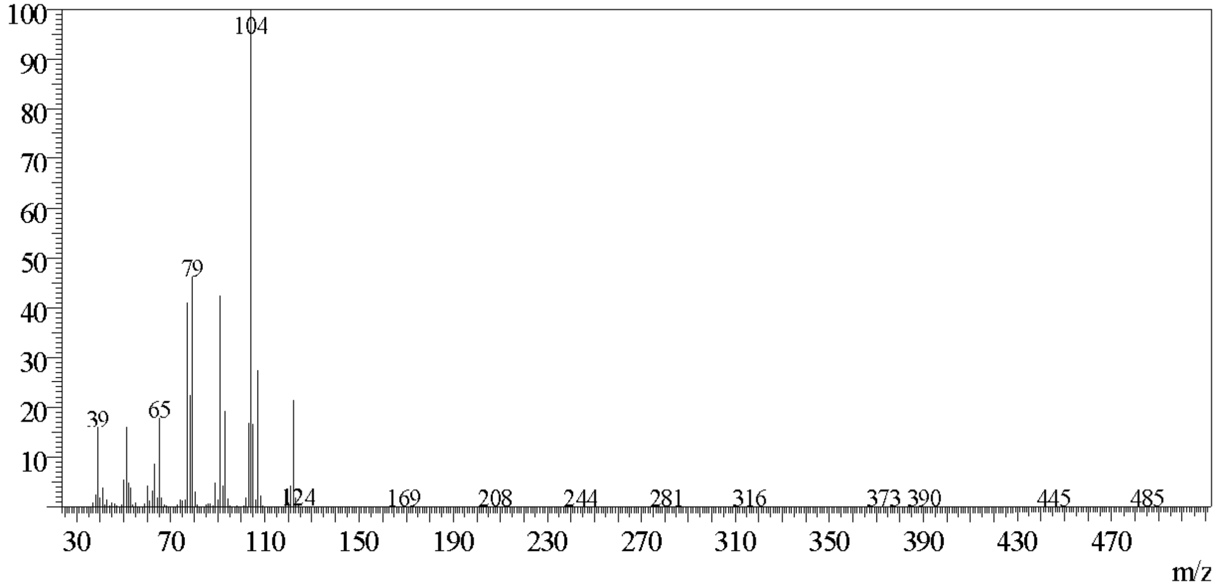

Figure S90. GC-MS of **4h**

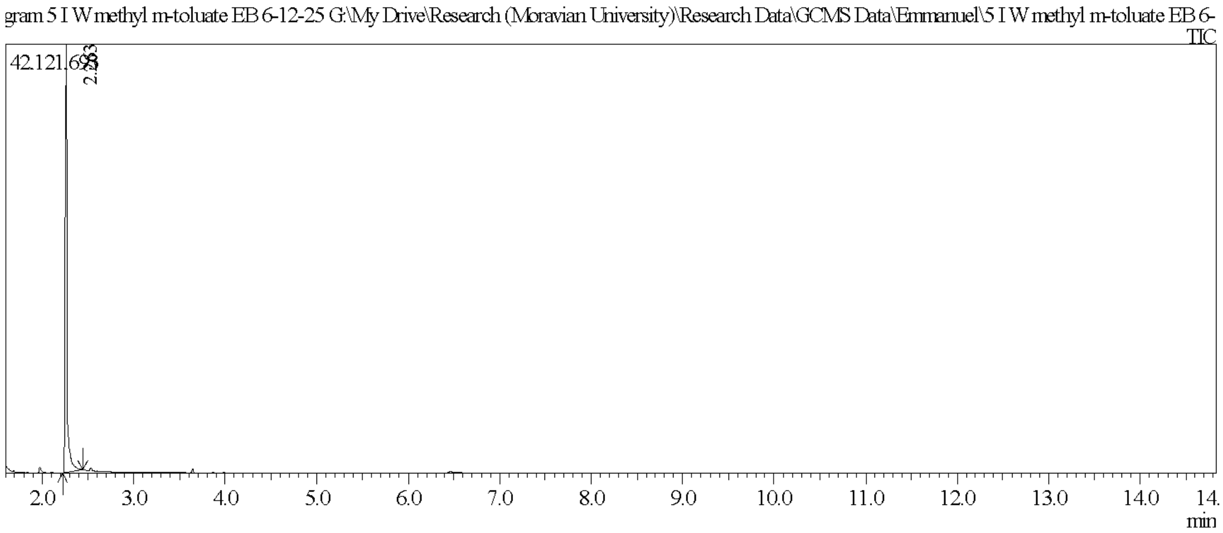

Spectrum

Peak#:1 R.Time:2.263(Scan#:134)
MassPeaks:325
RawMode:Averaged 2.260-2.270(133-135)
BG Mode:Calc. from Peak Group 1 - Event 1 Scan

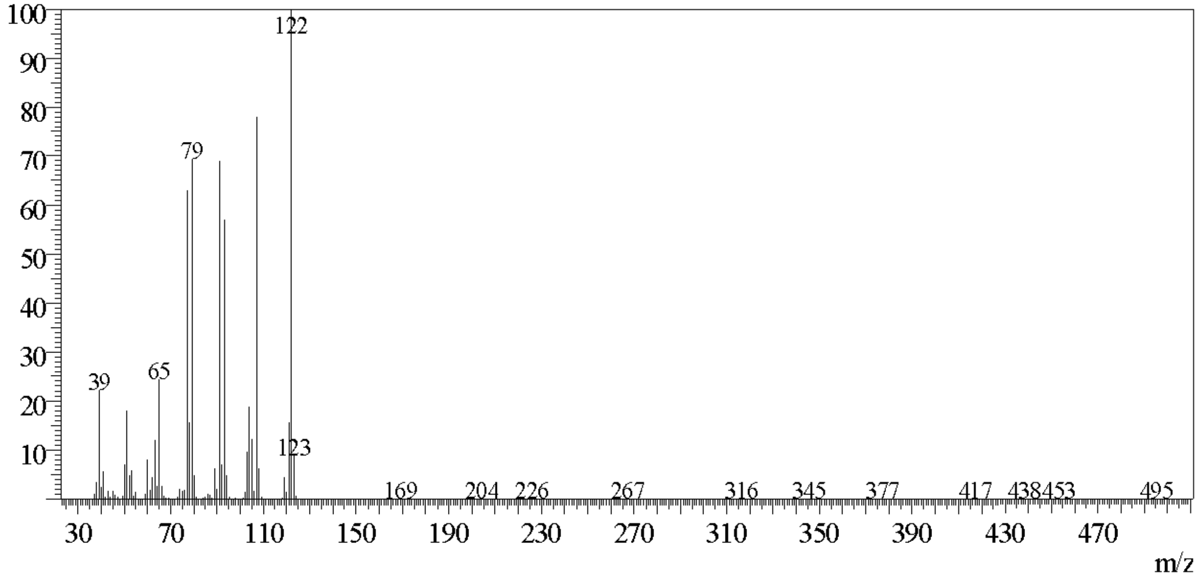

Figure S91. GC-MS of **4i**

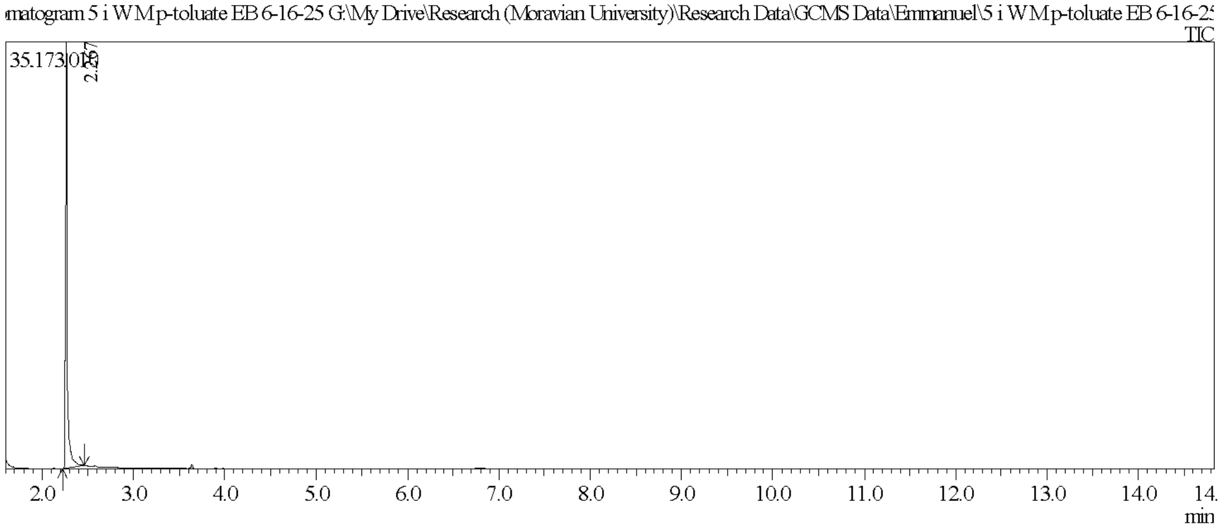

Spectrum

Peak#:1 R.Time:2.267(Scan#:134)
MassPeaks:301
RawMode:Averaged 2.260-2.270(133-135)
BG Mode:Calc. from Peak Group 1 - Event 1 Scan

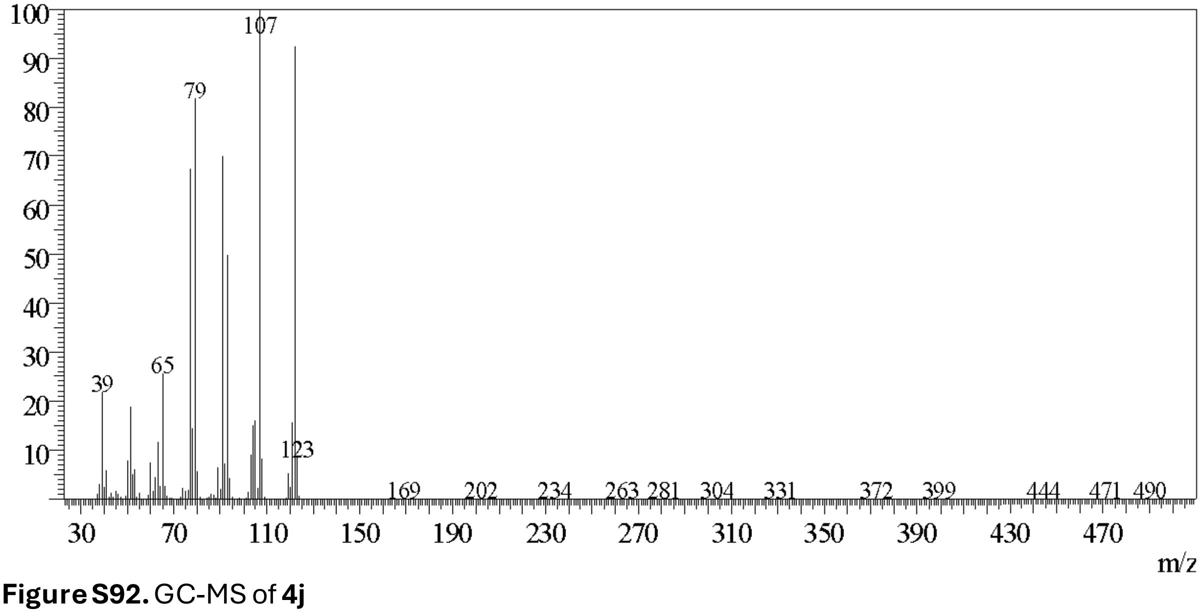

Figure S92. GC-MS of **4j**

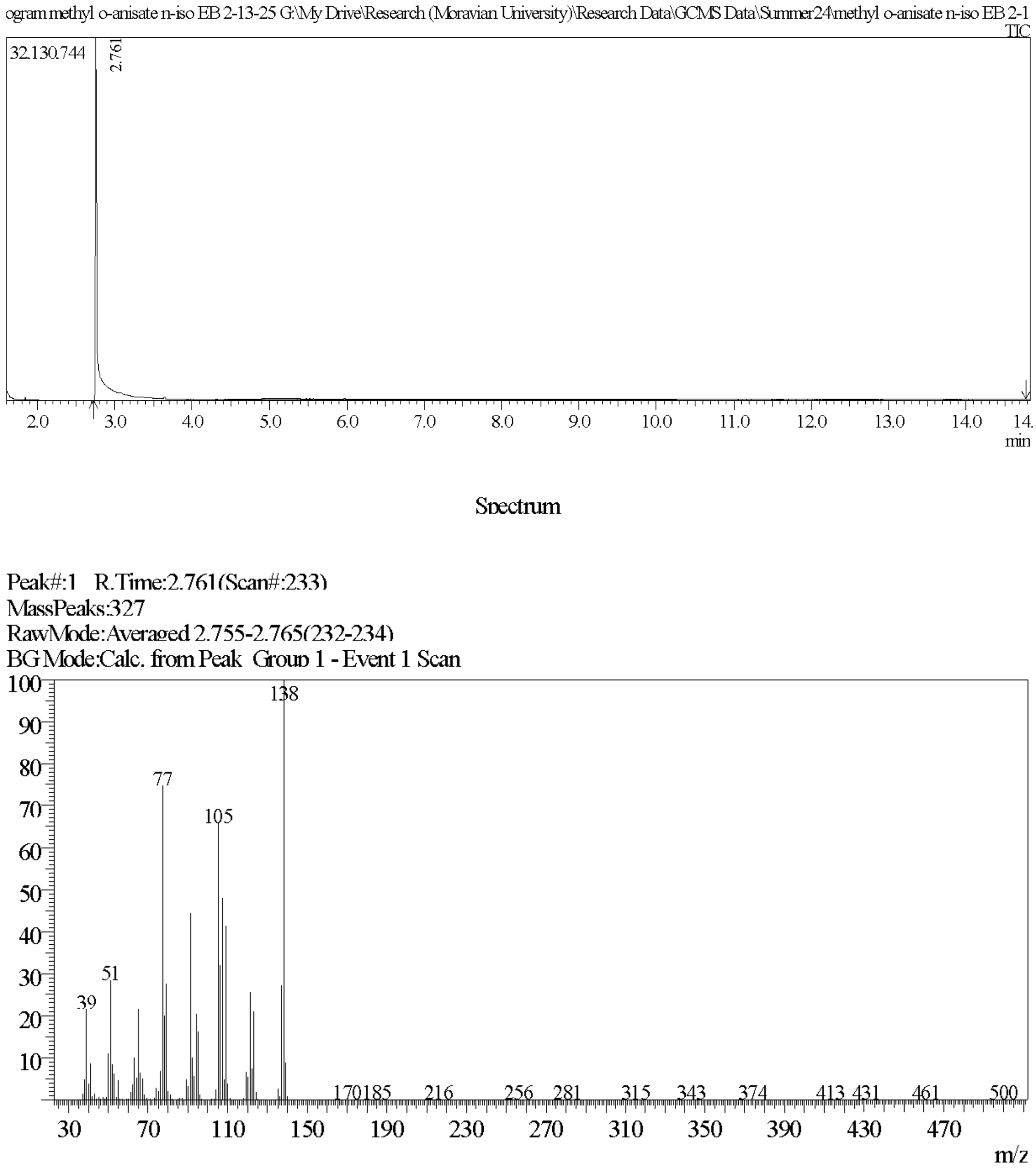

Figure S93. GC-MS of **4k**

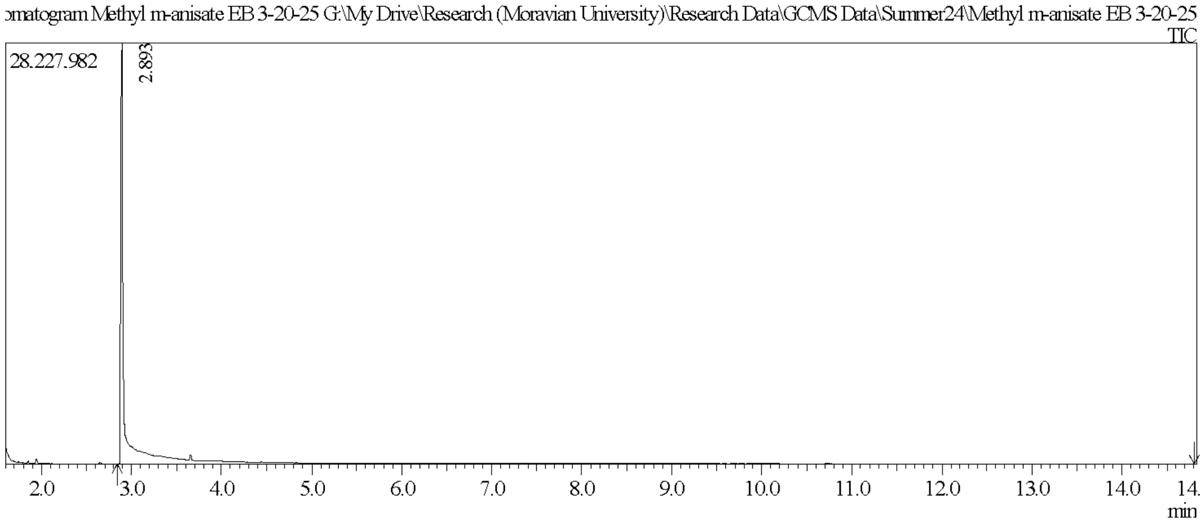

Spectrum

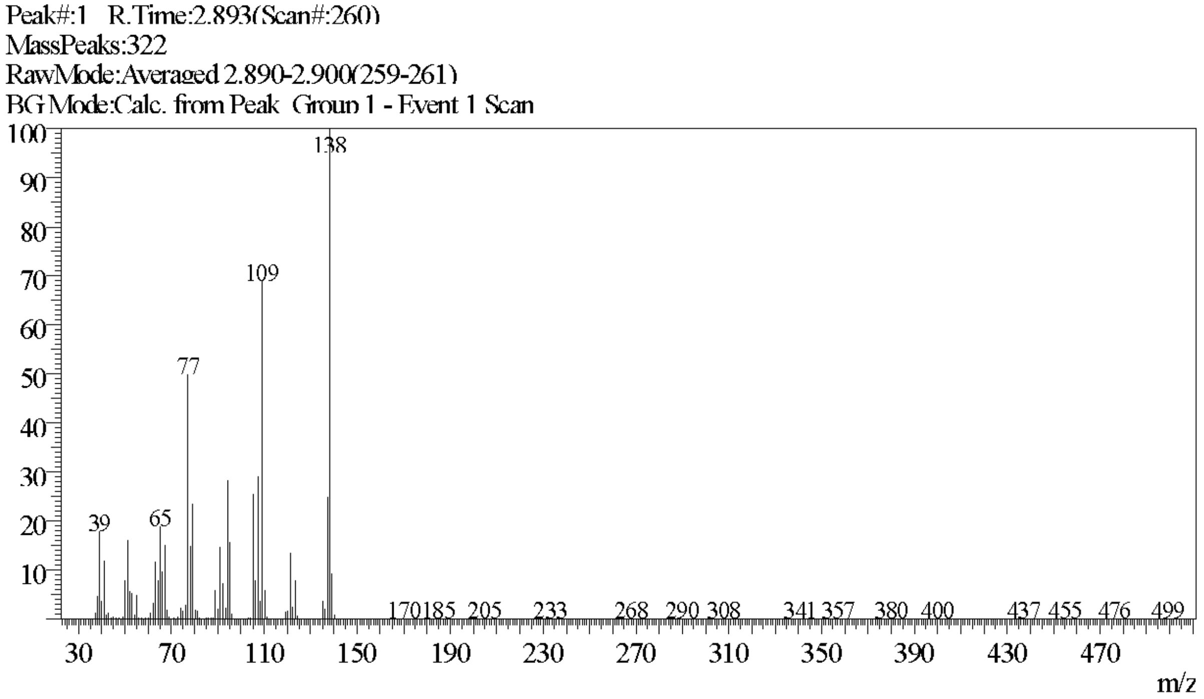

Figure S94. GC-MS of **4l**

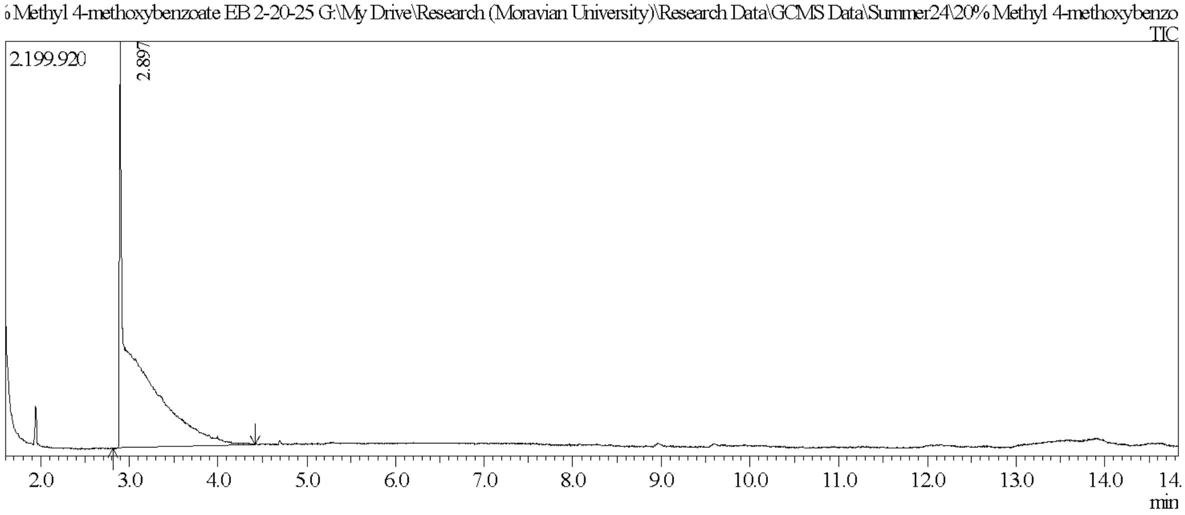

Spectrum

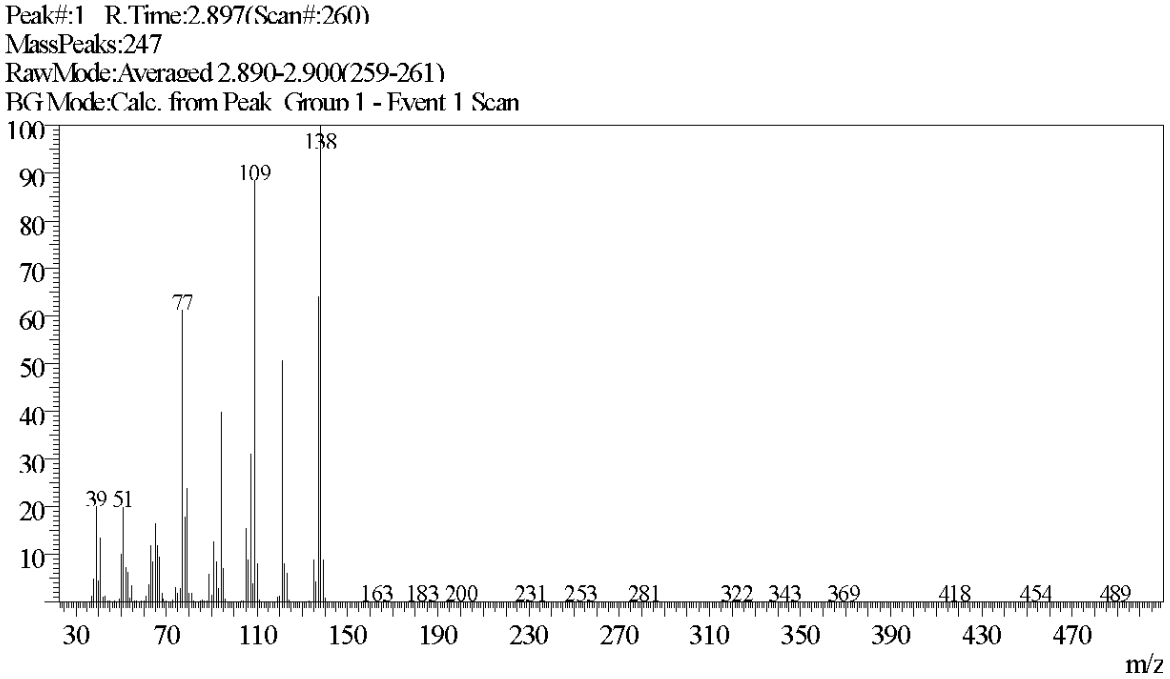

Figure S95. GC-MS of **4m**

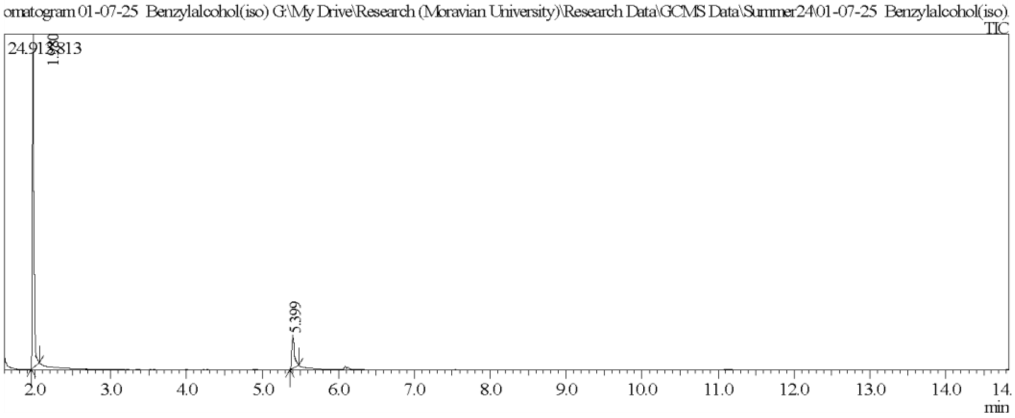

Spectrum

Peak#:1 R.Time:1.980(Scan#:77)
MassPeaks:283
RawMode:Averaged 1.975-1.985(76-78)
BG Mode:Calc. from Peak Group 1 - Event 1 Scan

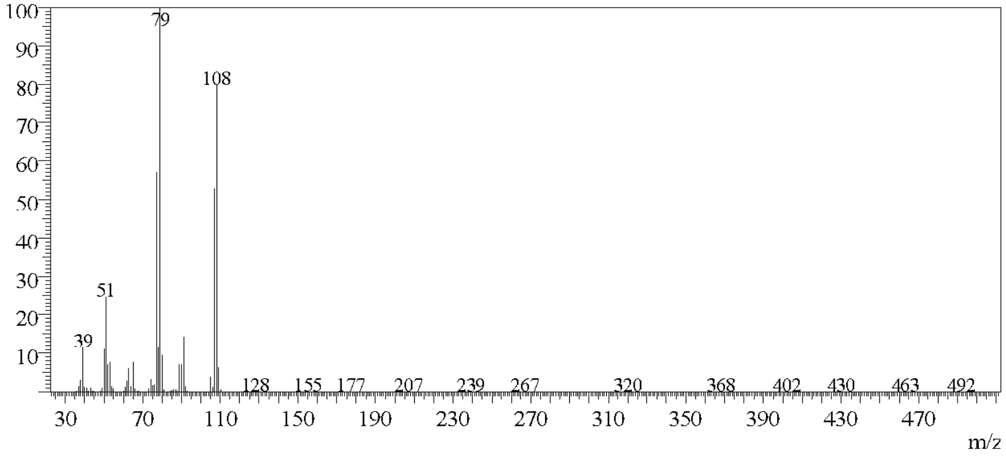

Spectrum

Peak#:2 R.Time:5.399(Scan#:761)
MassPeaks:278
RawMode:Averaged 5.395-5.405(760-762)
BG Mode:Calc. from Peak Group 1 - Event 1 Scan

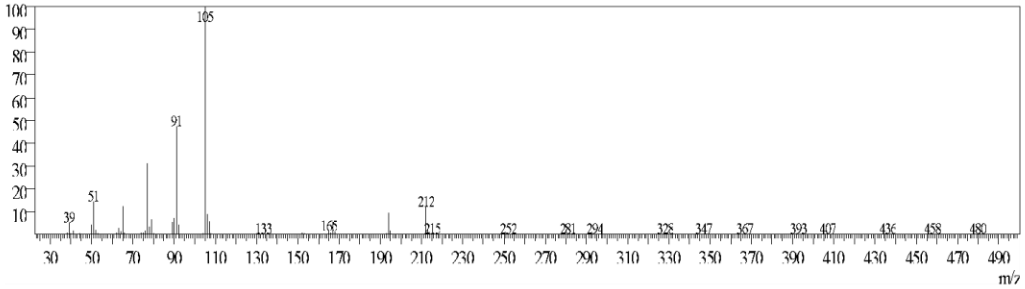

Figure S96. GC-MS of **4n**

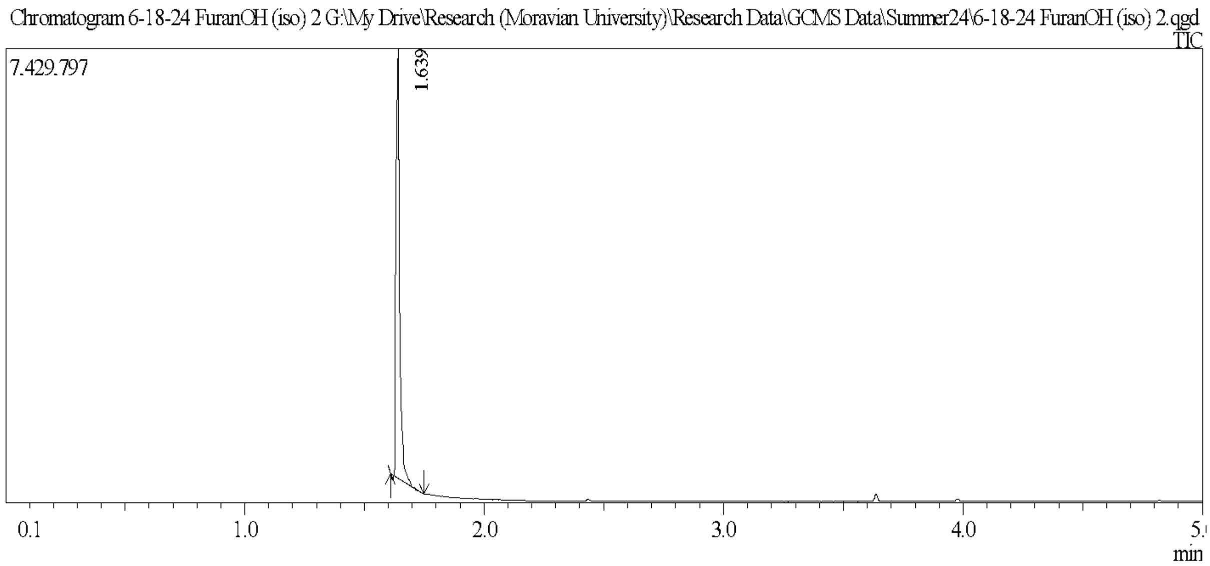

Spectrum

Peak#:1 R.Time:1.639(Scan#:9)
MassPeaks:246
RawMode:Averaged 1.635-1.645(8-10)
BG Mode:Calc. from Peak Group 1 - Event 1 Scan

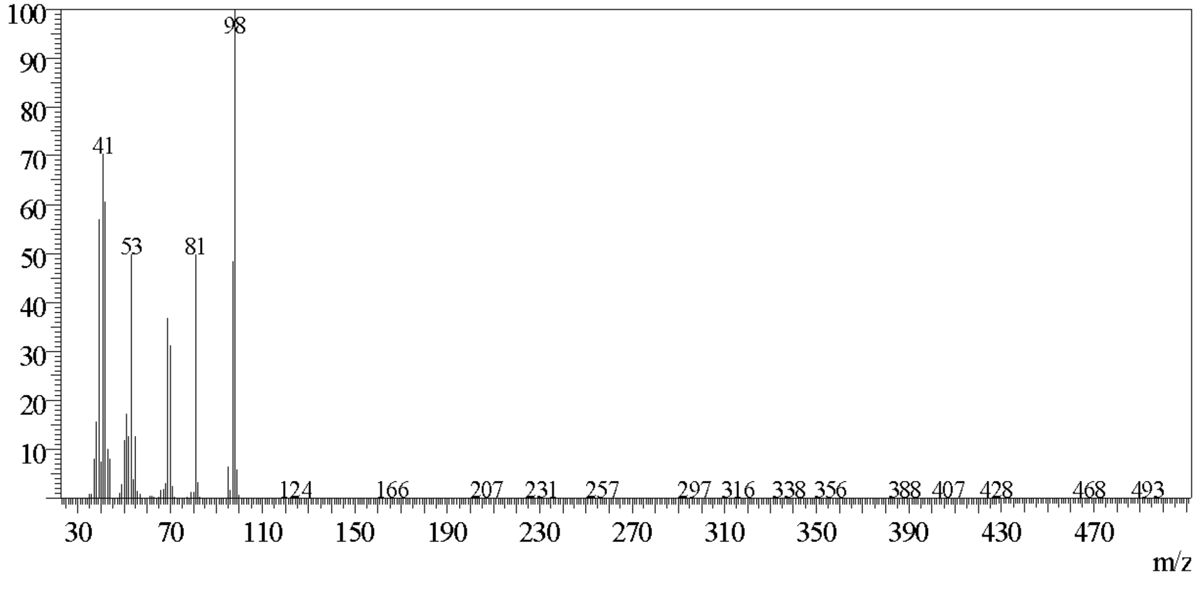

Figure S97. GC-MS of **4o**

Chromatogram 6-24-24 FuranOH F:\GCMS Data\gdf\Fianu\6-24-24 FuranOH.qgd

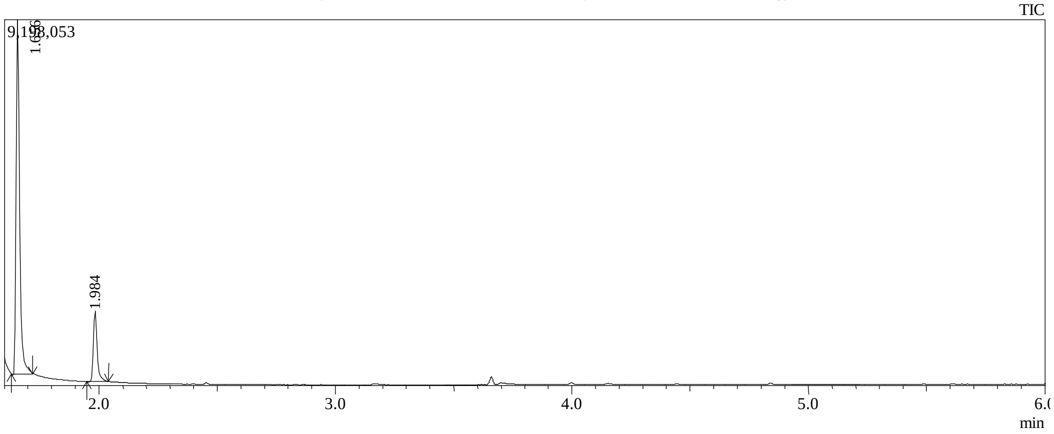

Peak Report TIC

Peak#	R.Time	I.Time	F.Time	Area	Area%	Height	Height%	A/H	Mark	Name
1	1.656	1.630	1.720	9026979	82.27	8898153	83.33	1.01	MI	
2	1.984	1.950	2.040	1945045	17.73	1779888	16.67	1.09	MI	

Spectrum

Peak#:1 R.Time:1.656(Scan#:12)
MassPeaks:230
RawMode:Averaged 1.650-1.660(11-13)
BG Mode:Calc. from Peak Group 1 - Event 1 Scan

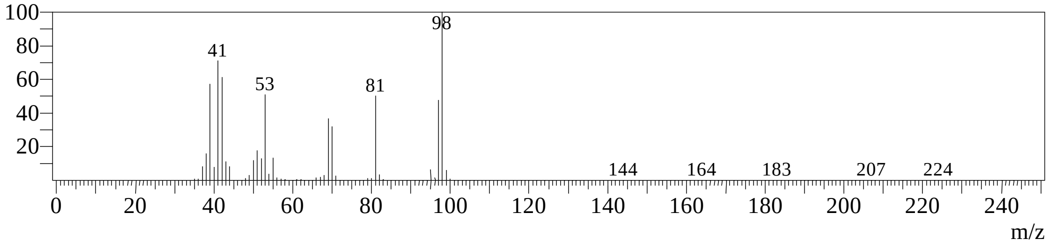

Spectrum

Peak#:2 R.Time:1.984(Scan#:78)
MassPeaks:273
RawMode:Averaged 1.980-1.990(77-79)
BG Mode:Calc. from Peak Group 1 - Event 1 Scan

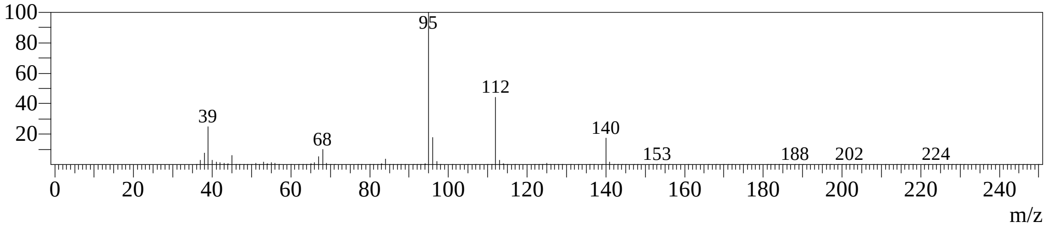

Figure S98. GC-MS of **4o** with 2.5 equivalents of PMHS

10. GC-MS for control experiments

GC-MS for Control Experiment 1

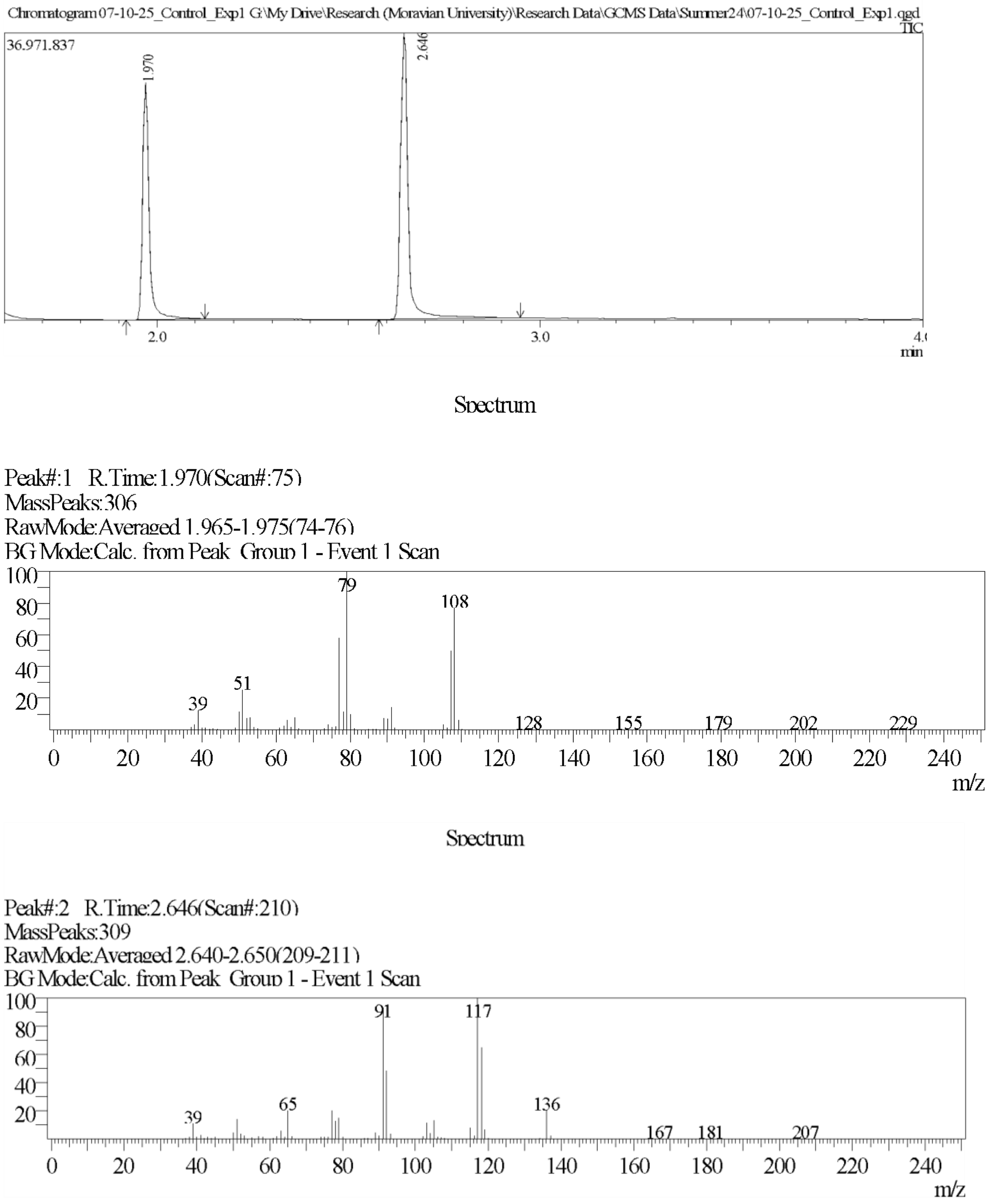

Figure S99. GC-MS of control experiment 1

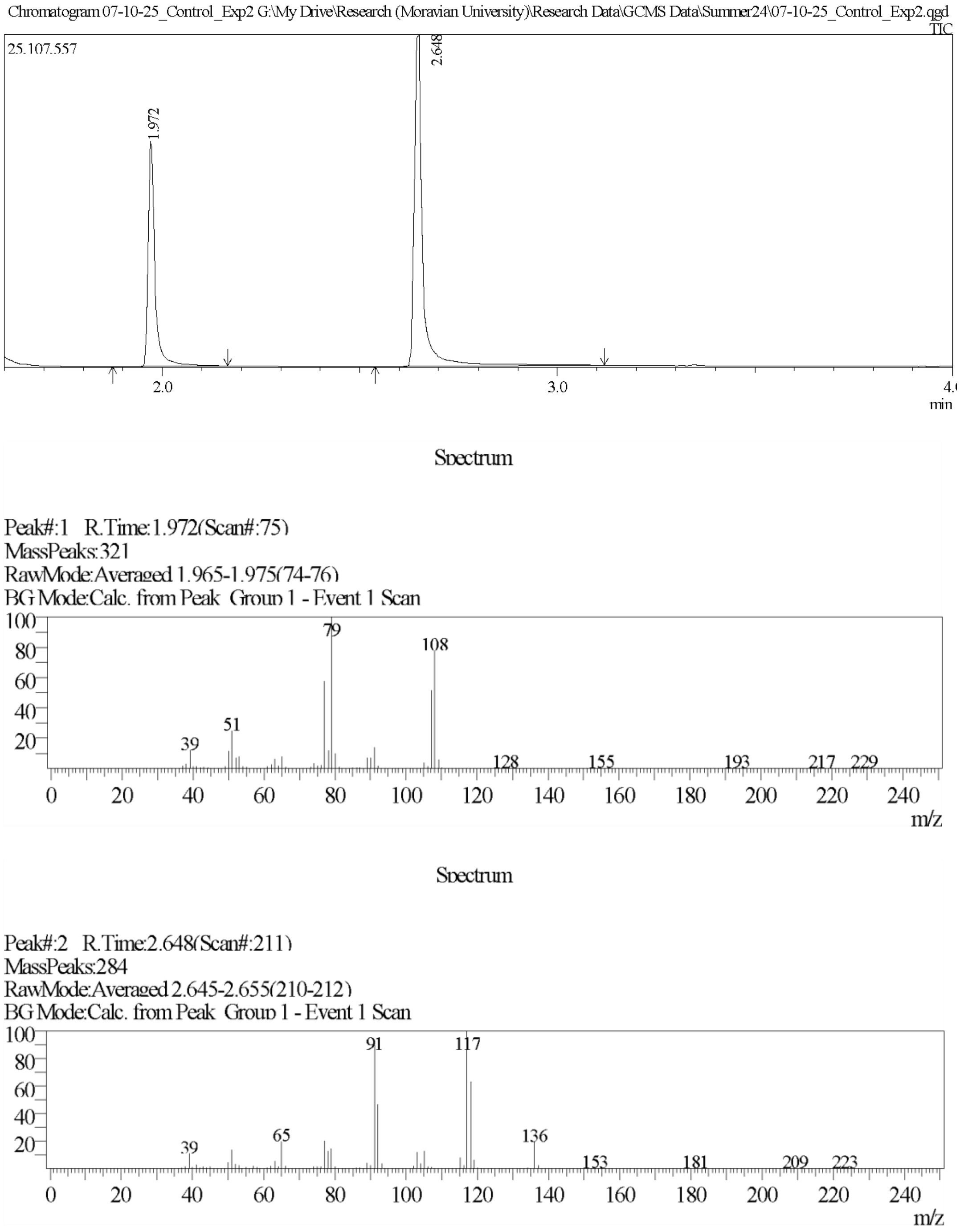

Figure S100. GC-MS of control experiment 2

11. GC-MS data on effects of catalyst loading and heat on the reduction of 1i and 3m

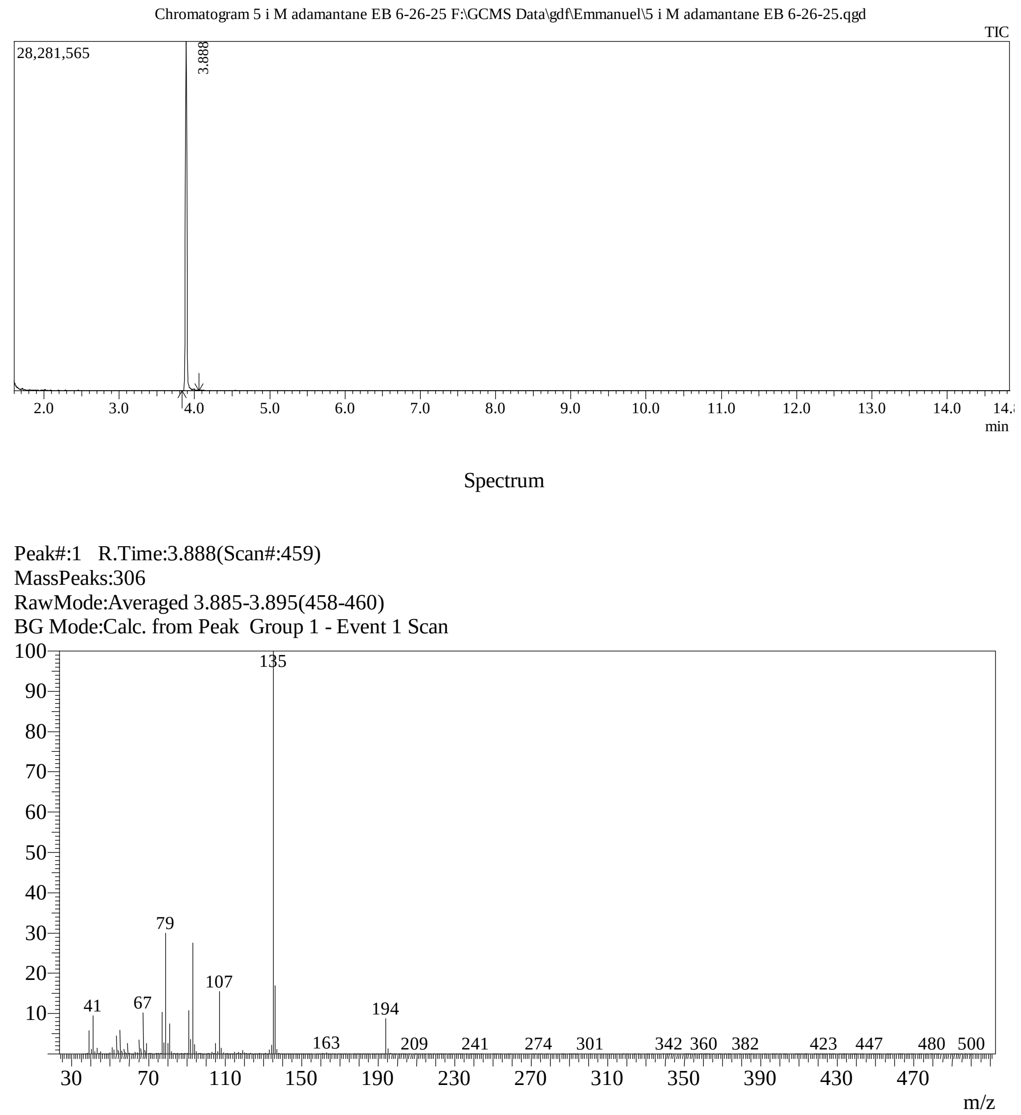

Figure S101. GC-MS for the reduction of **1i** with 5 mol% catalyst

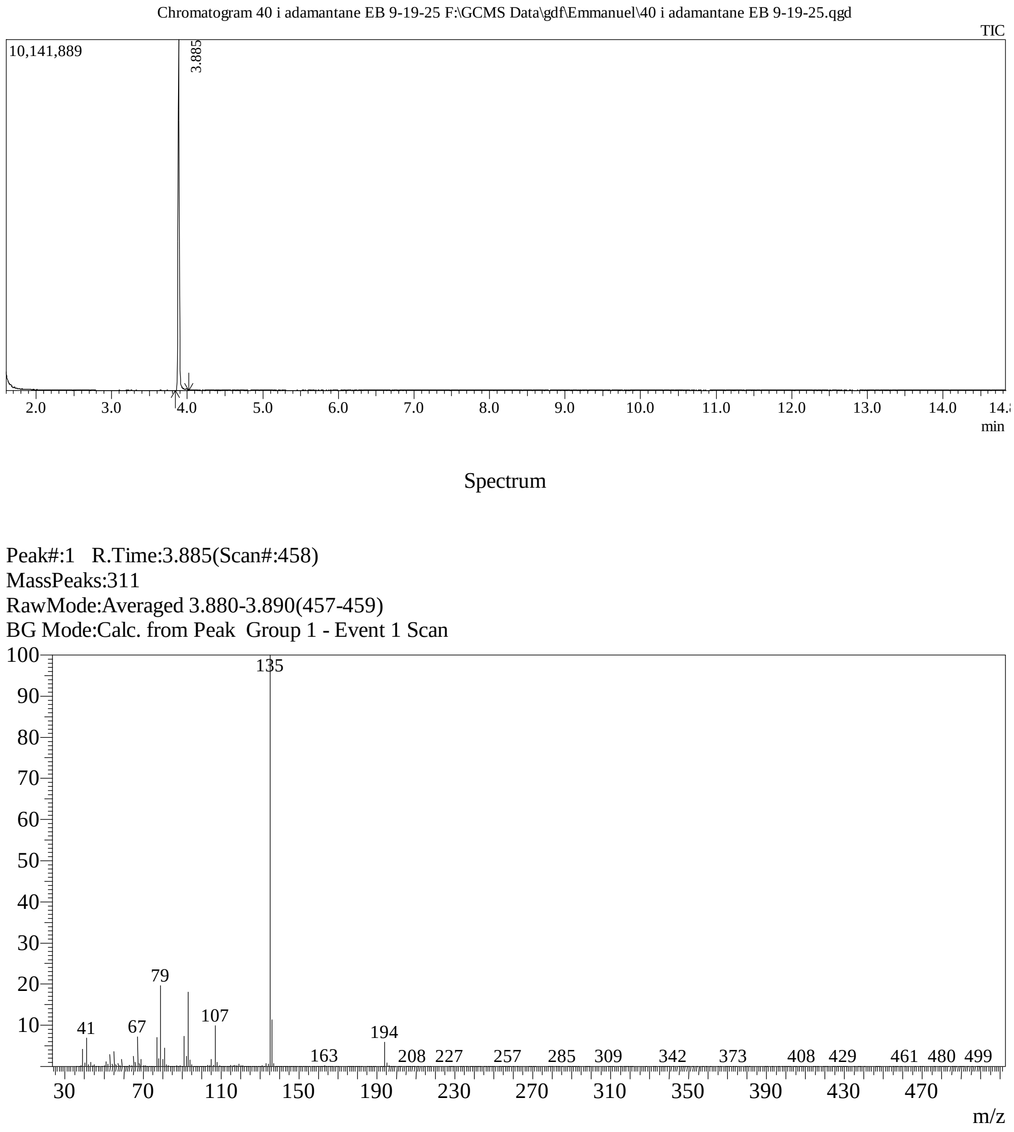

Figure S102. GC-MS for the reduction of **1i** with 40 mol% catalyst

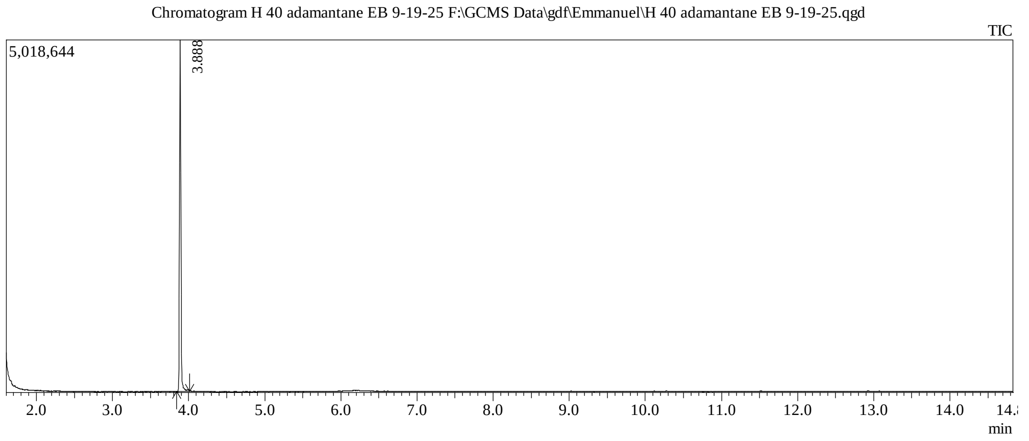

Spectrum

Peak#:1 R.Time:3.888(Scan#:458)
MassPeaks:266
RawMode:Averaged 3.880-3.890(457-459)
BG Mode:Calc. from Peak Group 1 - Event 1 Scan

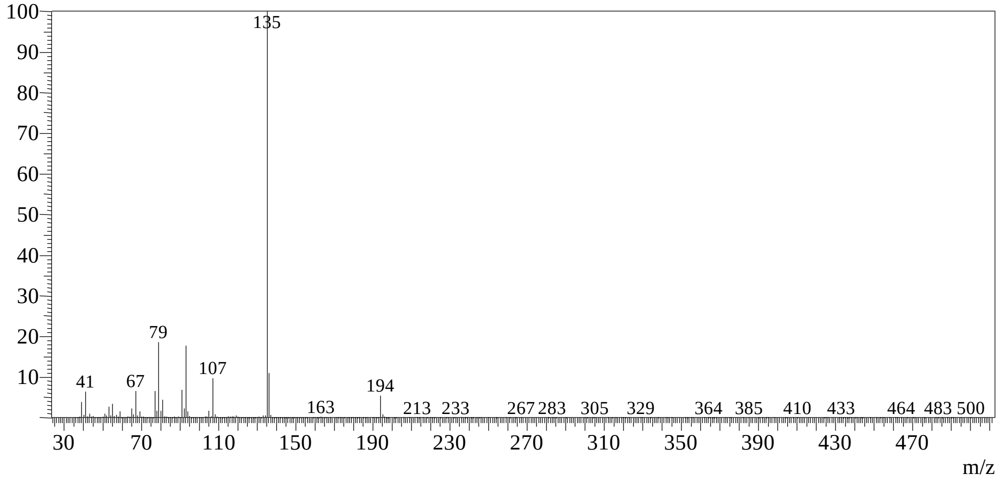

Figure S103. GC-MS for the reduction of **1i** with 40 mol% catalyst and at 85°C

Chromatogram Methyl 4-methoxybenzoate 2 EB 2-13-25 F:\GCMS Data\gdf\Emmanuel\Methyl 4-methoxybenzoate 2 EB 2-13-25.qgd

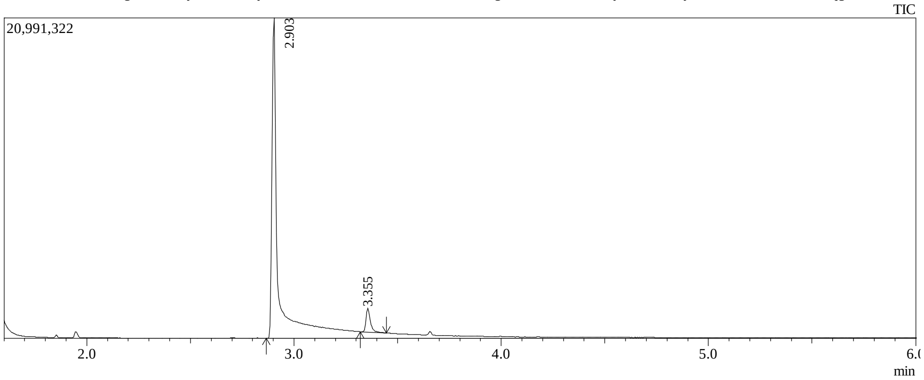

Peak Report TIC

Peak#	R.Time	I.Time	F.Time	Area	Area%	Height	Height%	A/H	Mark	Name
1	2.903	2.865	14.815	62668280	96.48	20967313	93.02	2.99	SV	
2	3.355	3.320	3.445	2287543	3.52	1573916	6.98	1.45	T	
				64955823	100.00	22541229	100.00			

Spectrum

Peak#:1 R.Time:2.903(Scan#:262)
MassPeaks:338
RawMode:Averaged 2.900-2.910(261-263)
BG Mode:Calc. from Peak Group 1 - Event 1 Scan

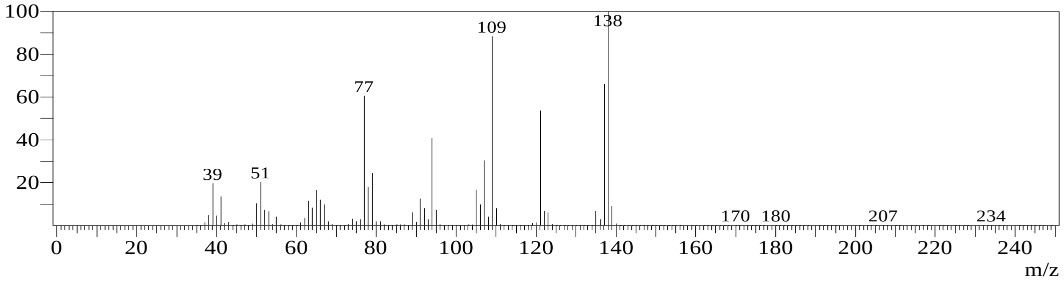

Spectrum

Peak#:2 R.Time:3.355(Scan#:352)
MassPeaks:313
RawMode:Averaged 3.350-3.360(351-353)
BG Mode:Calc. from Peak Group 1 - Event 1 Scan

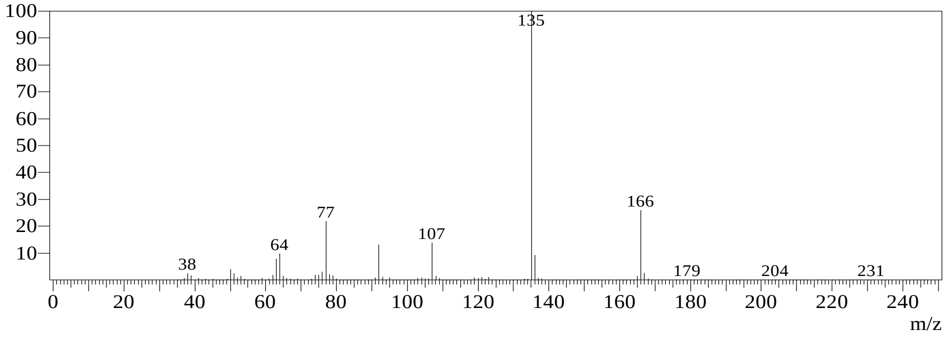

Figure S104. GC-MS for the reduction of **3m** to **4m** with 5 mol% catalyst

Chromatogram H 4-methoxybenzoate 5-1 EB 5-23-25 F:\GCMS Data\gdf\Emmanuel\H 4-methoxybenzoate 5-1 EB 5-23-25.qgd

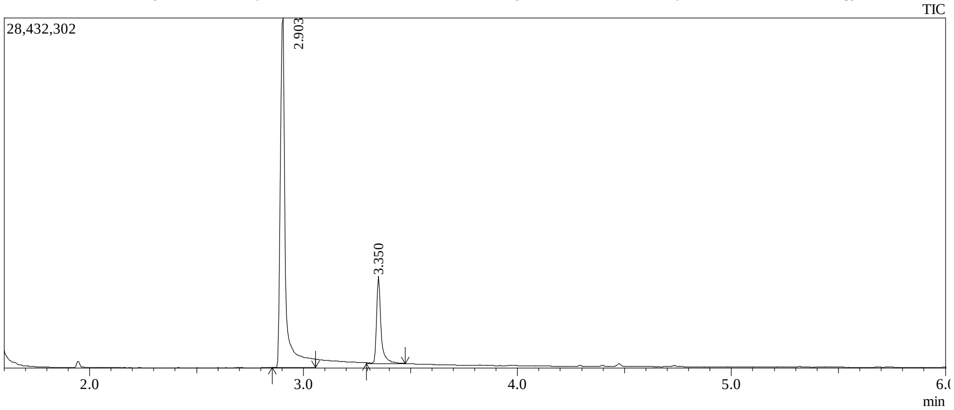

Peak Report TIC

Peak#	R.Time	I.Time	F.Time	Area	Area%	Height	Height%	A/H	Mark	Name
1	2.903	2.855	3.055	47014878	82.34	28384453	79.98	1.66	MI	
2	3.350	3.295	3.475	10082265	17.66	7106027	20.02	1.42	MI	
				57097143	100.00	35490480	100.00			

Spectrum

Peak#:1 R.Time:2.903(Scan#:262)
MassPeaks:282
RawMode:Averaged 2.900-2.910(261-263)
BG Mode:Calc. from Peak Group 1 - Event 1 Scan

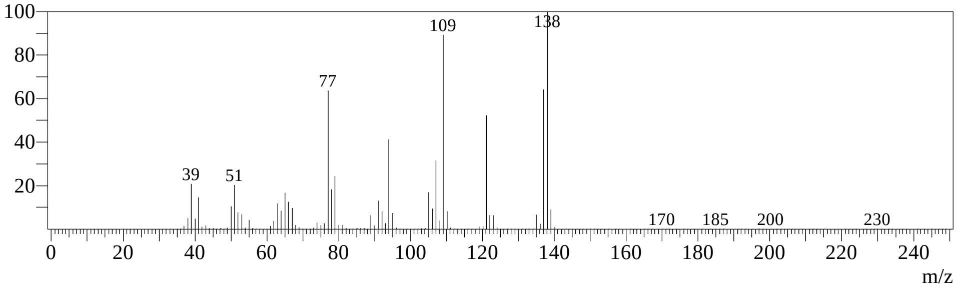

Spectrum

Peak#:2 R.Time:3.350(Scan#:351)
MassPeaks:292
RawMode:Averaged 3.345-3.355(350-352)
BG Mode:Calc. from Peak Group 1 - Event 1 Scan

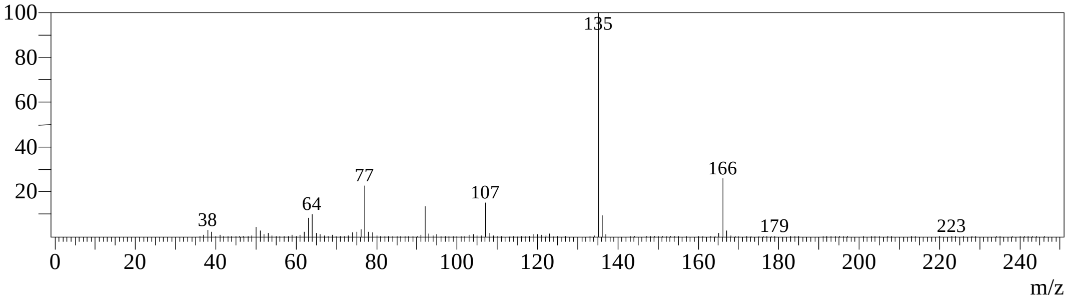

Figure S105. GC-MS for the reduction of **3m** to **4m** with 5 mol% catalyst at 85 °C
